# Identification of ANXA1 as a Novel Upstream Negative Regulator of Notch1 Function in AML

**DOI:** 10.1002/advs.202409726

**Published:** 2024-10-24

**Authors:** Gang Shao, Xi Wang, Yiting Zheng, Junjie Ma, Lei Wang, Zhibin Yan, Zeyu Sun, Shuyuan Zhang, Hongzhang Wu, Yudie Lv, Hemiao Huang, Jianhu Li, Tianyi Zhu, Bing Yang, Nanxi Wang, Tao Chen, Xuancheng Guo, Yuanting Jin, Jian Kang, Huafeng Wang, Yihai Cao, Caiyun Fu

**Affiliations:** ^1^ Zhejiang Provincial Key Laboratory of Silkworm Bioreactor and Biomedicine College of Life Sciences and Medicine Zhejiang Sci‐Tech University Hangzhou 310018 China; ^2^ Department of Oncology No.903 Hospital of PLA Joint Logistic Support Force Hangzhou 310013 China; ^3^ College of Life Sciences China Jiliang University Hangzhou 310018 China; ^4^ State Key Laboratory for Diagnosis and Treatment of Infectious Diseases The First Affiliated Hospital Zhejiang University School of Medicine Hangzhou 310003 China; ^5^ Department of Neurosurgery Zhejiang Cancer Hospital Hangzhou Institute of Medicine (HIM) Chinese Academy of Sciences Hangzhou 310022 China; ^6^ Department of Hematology The First Affiliated Hospital Zhejiang University School of Medicine Hangzhou 310003 China; ^7^ Zhejiang Provincial Key Laboratory for Cancer Molecular Cell Biology Life Sciences Institute Zhejiang University Hangzhou 310058 China; ^8^ State Key Laboratory Cultivation Base for TCM Quality and Efficacy School of Pharmacy Nanjing University of Chinese Medicine Nanjing 210046 China; ^9^ Sartorius (Shanghai) Trading Co., Ltd. Shanghai 200120 China; ^10^ Hangzhou Acnovia Biotech Co., Ltd. Hangzhou 310018 China; ^11^ Oncogenic Signalling and Growth Control Program Peter MacCallum Cancer Centre 305 Grattan street Melbourne Victoria 3000 Australia; ^12^ Sir Peter MacCallum Department of Oncology University of Melbourne Melbourne Victoria 3000 Australia; ^13^ Department of Microbiology Tumor and Cell Biology Karolinska Institute Stockholm 171 77 Sweden; ^14^ Key Laboratory of Preclinical Study for New Drugs of Gansu Province Research Unit of Peptide Science Chinese Academy of Medical Sciences 2019RU066 Lanzhou University Lanzhou 730000 China

**Keywords:** acute myeloid leukemia, annexin a1, anxa1‐notch1‐p15 signal axis, notch1, peptide inhibitors, protein‐protein interaction

## Abstract

Abnormal Notch1 expression has an important role in tumorigenesis. However, upstream control mechanisms for Notch1 are still insufficiently understood. Acute myeloid leukemia (AML) is one of the most common and lethal blood malignancies with limited possibilities for treatment. Thus, new therapeutic targets are urgently needed to improve current ineffective therapies. Herein, high Annexin A1 (ANXA1) expression is found correlated with hyperproliferation of AML cells, and then ANXA1 is identified as a novel negative regulator of Notch1 function in AML. Mechanistically, ANXA1 directly bound to the intracellular domain of Notch1 (NICD) to target this tumor suppressor for degradation. Furthermore, NICD executed its tumor suppressive function through activation of the p15 promoter. Thus, ablation of the Notch1‐p15‐mediated tumor suppression by ANXA1 provided a novel mechanism of AML proliferation. In human AML patients, a mutual exclusive relation is discovered between ANXA1 and Notch1/p15, corroborating mechanistic discovery. On the basis of these results, it is reasonably speculated that targeting ANXA1 would provide an effective approach for treatment of AML. In support of this new therapeutic paradigm, provided proof‐of‐concept data by antagonizing ANXA1 using NICD inhibitory peptides.

## Graphical abstract

High ANXA1 expression correlates with hyperproliferation of AML cells because ANXA1 interacts with NICD to target this tumor suppressor for degradation. Based on the novel mechanism of ablation of the Notch1‐p15‐mediated tumor suppression in AML hyperproliferation, targeting ANXA1 provides an effective approach for AML treatment, in which antagonizing ANXA1 using NICD inhibitory peptides is an effective strategy for AML treatment.

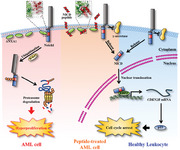

## 1 Introduction

Notch1 signaling is an evolutionarily conserved pathway that regulates cell fate decisions in neuronal, immune, cardiovascular, and endocrine systems.^[^ ^1^ ^]^ Considering the key role of Notch1 in regulating cell processes, such as proliferation, differentiation, and apoptosis. The abnormally expressed Notch1 may be involved in the genesis and development of malignant tumors.^[^ ^2^ ^]^ Notably, the expression level and function of Notch1 vary in different kinds of cancers. Recent studies reported the positive effects of Notch1 in promoting proliferation and metastasis in hepatocellular carcinomas, bladder tumor‐initiating cells, colorectal cancers, and triple‐negative breast cancers, but the opposite results were found in skin and forebrain tumor subtypes.^[^ ^3^ ^]^ Furthermore, mutations and subsequent activation of the *NOTCH1* gene occur in more than 65% of T‐cell acute lymphoblastic leukemia (T‐ALL) patients, but are rare in acute myeloid leukemia (AML) patients.^[^ ^4^ ^]^ Notch1 is a tumor‐suppressive gene in AML.^[^ ^5^ ^]^ Notch1 is a single‐pass transmembrane protein that releases its intracellular domain (NICD) via γ‐secretase activity.^[^ ^6^ ^]^ The NICD then binds to the recombination signal binding protein for immunoglobulin kappa J region (RBPJ, also known as CSL) transcription factor complex after nuclear translocation, to trigger the activation of target genes.^[^ ^7^ ^]^ However, as an important target for disease treatment, the regulatory factors affecting NICD functions remain largely unexplored.

Annexin A1 (ANXA1) is a Ca^2+^‐dependent phospholipid‐binding protein that participates in a variety of important cell processes, such as inflammation, cell survival, proliferation, apoptosis, differentiation, and migration.^[^ ^8^ ^]^ ANXA1 has the ability to inhibit the activity of cytosolic phospholipase A2 and cyclooxygenase 2 to exhibit anti‐inflammatory, antipyretic, and anti‐hyperalgesic activity, as well as it can be induced in human cancer cells and mice by anti‐inflammatory drugs to inhibit the activation of NF‐κB by binding to its p65 subunit.^[^ ^8^, ^9^ ^]^ ANXA1 regulates the extracellular signal‐regulated kinase (ERK) pathway at a proximal location, by Src Homology 2 (SH2) domain‐independent association with the adapter protein Grb‐2, to exert its antiproliferative activity by ERK‐mediated disruption of the actin cytoskeleton and ablation of cyclin D1, but not by ERK‐mediated induction of p21^cip/waf^, as well as by the constitutive activation of the mitogen‐activated protein kinase (MAPK)/ERK pathway linked to its phosphorylation by epidermal growth factor.^[^ ^10^ ^]^ Notably, ANXA1 has been implicated in complex and contradictory functions in cancers, including tumor growth, metastasis, invasion, and drug resistance.^[^ ^11^ ^]^ ANXA1 negatively regulated the phosphorylation of GSK3β, inhibited the epithelial‐mesenchymal transformation and migration and invasion of non‐small cell lung cancer (NSCLC) cells.^[^ ^12^ ^]^ Ribonucleotide reductase subunit M2 stabilizes ANXA1 and activates the AKT pathway, thereby promoting sunitinib resistance.^[^ ^13^ ^]^ ANXA1 acts as an important homeostatic protein by binding to the target protein on the membrane of some cancer cells.^[^ ^14^ ^]^ As a therapeutic and diagnostic target, ANXA1 is a double‐edged sword, depending on the tumor type.^[^ ^15^ ^]^ The current study found that among B‐cell malignancies, ANXA1 was a specific marker for hairy cell leukemia.^[^ ^16^ ^]^ However, the function and mechanisms of ANXA1 in AML are largely unclear.

Herein, we identified ANXA1 as a novel upstream negative regulator of Notch1 through the direct interaction between the 2,050–2,060 amino acid domain of NICD and the C‐terminal 214–228 amino acid domain of ANXA1 on cell membranes. We also identified p15, a cyclin‐dependent kinase inhibitor, as a novel target gene of NICD, which was regulated by binding to the promotor of p15. In human AML patients, we discovered a mutually exclusive relationship between ANXA1 and Notch1/p15, which confirmed our mechanistic finding. In brief, we uncovered an unanticipated ablation of the Notch1‐p15‐mediated tumor suppression by ANXA1 in AML through the ubiquitination‐proteasome degradation of NICD to block the transcriptional activation of p15 in vitro and in vivo. On the basis of these results, we speculated that targeting ANXA1 would provide an effective approach for treatment of AML. In support of this new therapeutic paradigm, we provided proof‐of‐concept data by antagonizing ANXA1 using NICD inhibitory peptides. Thus, these findings reveal a key role of ANXA1 in the negative regulation of Notch1‐p15‐mediated tumor suppression in AML. This mechanistic finding may provide a novel concept for the further development of anti‐AML drugs targeting direct interactions between ANXA1 and Notch1 on cell membranes.

## 2 Results

### 2.1 Negative Regulation of Notch1 Expression by ANXA1 in AML Cells

**Figure 1:**
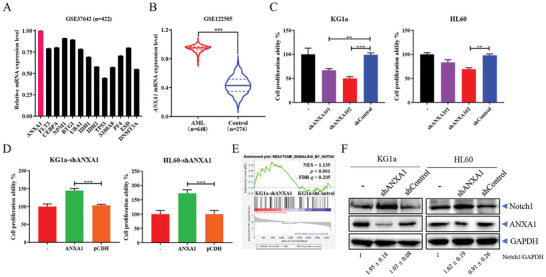
The expression level of ANXA1 is upregulated in AML patients, which promotes the proliferation of AML cells by down‐regulating Notch1 expression. (A) mRNA expression levels of AML biomarkers in bone marrow mononuclear cells from AML patients (*n* = 422). (B) *ANXA1* mRNA expression levels in peripheral blood or bone marrow samples of AML patients (*n* = 648) and non‐leukemia people (*n* = 274) from GSE122505 dataset. Student's *t*‐test, ^***^ *p* < 0.001. (C) The cell proliferation ability detected by cell counting after 96 h of culture of KG1a or HL60 cells transiently transfected with shANXA1 or shControl plasmids. (D) The cell proliferation ability detected by cell counting after 96 h of culture of KG1a‐ or HL60‐ shANXA1 cells transiently transfected with ANXA1 overexpression or pCDH control plasmids. (E) Gene set enrichment analysis of proteomic data from shANXA1 cells and shControl cells of KG1a. *p* < 0.001. (F) Western blotting of Notch1 and ANXA1 expression in KG1a and HL60 cells stably transfected with shANXA1 or shControl plasmids. Data in (C, D and F) are means ± s.e.m. of three independent experiments, *t*‐test, ^**^ *p* < 0.01, ^***^ *p* < 0.001.

To identify novel signaling molecules and therapeutic targets in AML, we performed a study of Gene Expression Profile (GEP) analyses from Gene Expression Omnibus (GEO) and The Cancer Genome Atlas (TCGA) datasets, according to the review of molecular biomarkers in AML.^[^ ^17^ ^]^ ANXA1 was identified as one of the most highly expressed biomarkers in AML (**Figures** **1**A,B and S1, Supporting Information). Therefore, we focused on defining functional roles of ANXA1 in AML using a loss‐of‐function approach by knocking‐down ANXA1 using shRNAs. Two shRNAs with different stem structures targeting ANXA1 were generated, in which shANXA1#2 was more potent in mediating ANXA1 knockdown (Figure S2A, Supporting Information). The KG1a and HL60 cell lines were derived from patients with the poorest prognosis AML subtype (FAB M0) and the relatively better prognosis subtype (FAB M2), respectively (Figure S2B, Supporting Information). Depletion of ANXA1 using knockdown shRNA technology significantly reduced cell proliferation in both KG1a and HL60 cells (Figure 1C). To verify the functional specificity of ANXA1‐knockdown by shRNA, overexpression of ANXA1 in ANXA1‐knockdown KG1a and HL60 cells was shown to promote cell proliferation (Figures 1D and S2C, Supporting Information).

To investigate potential molecular mechanisms underlying ANXA1‐induced hyperproliferation in AML cells, quantitative proteomics was performed. The results of gene set enrichment analysis showed that the Notch signal pathway was enriched in cells with stable knockdown of ANXA1 (Figure 1E). Further, we confirmed that knockdown of ANXA1 significantly increased the expression levels of Notch1 in KG1a and HL60 cells (Figure 1F).

### 2.2 Notch1 Interacts Directly with ANXA1 on the Cell Membrane

**Figure 2:**
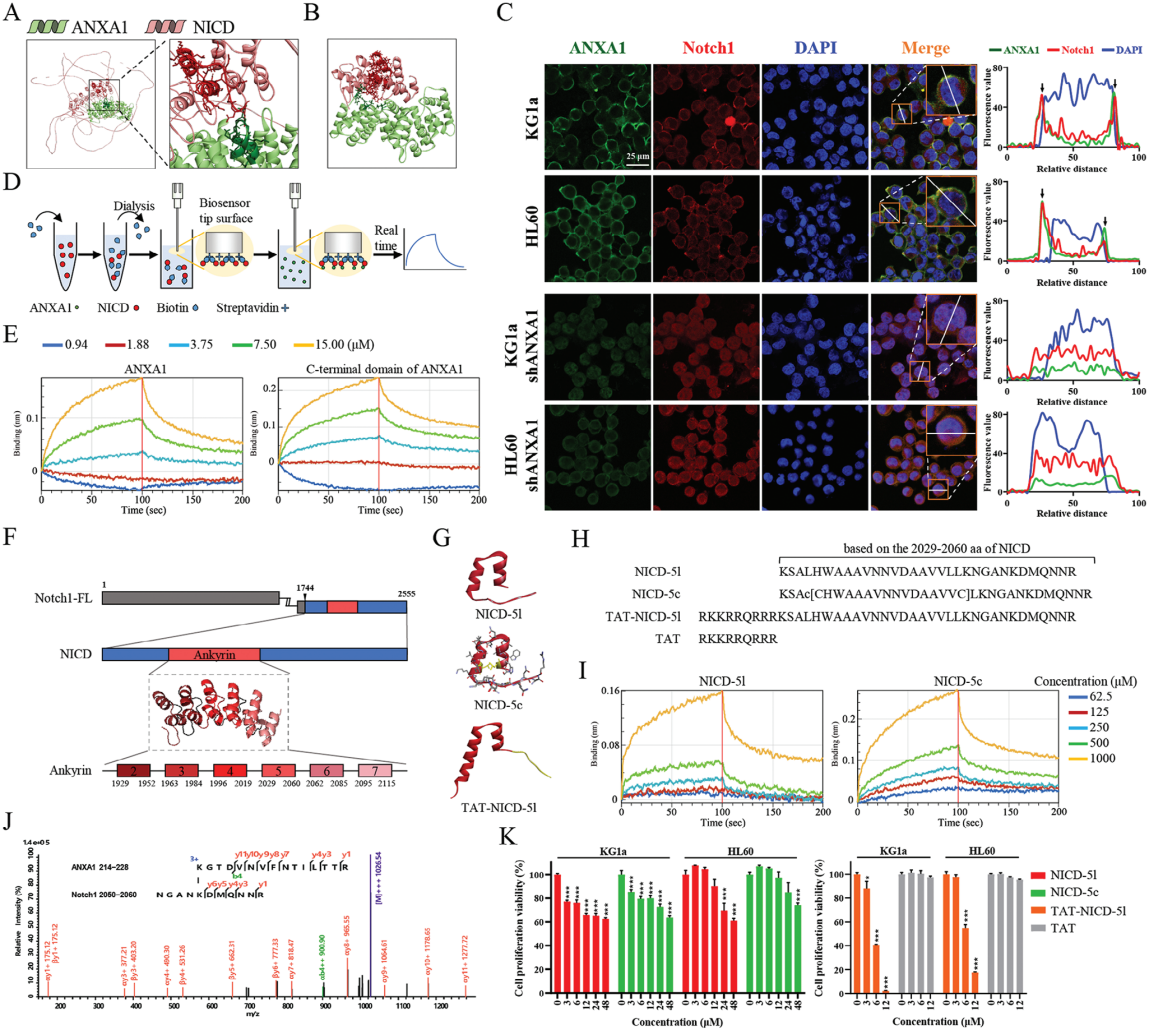
The 2,050–2,060 amino acids domain of NICD mediates the direct interaction with the C‐terminal 214–228 amino acid domain of ANXA1 on the cell membrane. (A) Model diagram of the interaction between NICD and ANXA1 predicted by AlphaFold2. (B) Model diagram of the docking prediction of NICD ankyrin domain (PDB: 1YYH) with ANXA1 (PDB: 1MCX) by ClusPro. (C) Representative images and profile analyses of Notch1 and ANXA1 colocalization in KG1a and HL60 cells or shANXA1 cells taken by laser scanning confocal microscope. Orange squares indicate regions of interest for Manders overlap coefficient analyses and white solid lines indicate regions of interest for profile analyses. The arrows indicate the colocalization and scale bar represent 25 µm. (D) Schematic diagram of BLI analysis. (E) BLI analysis of the interactions between NICD and indicated ANXA1 or C‐terminal of ANXA1. (F) Schematic diagram of the corresponding positions of the designed peptides, including the model diagram of the ankyrin domain of NICD (PDB: 1YYH). (G) 3D structural model of the NICD‐5l peptide and NICD‐5c peptide designed according to 2029–2060 amino acids of ankyrin domain of NICD (PDB: 1YYH). 3D structural model of the TAT‐NICD‐5l peptide was predicted by AlphaFold2. “l” for linear peptides and “c” for cyclic peptides. (H) Peptide sequences of NICD‐5l, NICD‐5c, TAT‐NICD‐5l and TAT. (I) BLI analysis of the interactions between ANXA1 and indicated NICD‐5l peptide or NICD‐5c peptide. (J) Mass spectrometry results of the interaction region between ANXA1 and NICD identified by chemical cross‐linking coupled with mass spectrometry. (K) The cell proliferation viability of KG1a and HL60 cells was detected by CCK8 assay after treatment with NICD‐5l peptide and NICD‐5c peptide for 72 h, or TAT‐NICD‐5l peptide and TAT peptide for 24 h, at indicated concentration. Data are means ± s.e.m. of three independent experiments, one‐way ANOVA with Dunnett's test, compared with 0 µM group, ^*^ *p* < 0.05, ^***^ *p* < 0.001.

Little is known about the regulators involved in upstream control of Notch1 function. We hypothesized that inhibition of Notch1 by ANXA1 might entail direct physical interaction between these two proteins on the cell membrane. Prediction by AlphaFold2 (**Figure** **2**A) and ClusPro (Figure 2B) showed the possibility of the interaction between ANXA1 and Notch1, especially in the C‐terminal domain of ANXA1 and the ankyrin domain of NICD. Then, we performed laser confocal microscope analyses to evaluate this interaction, and we found that ANXA1 was co‐localized with Notch1 on the cell membrane, but co‐localization was lost after knocking‐down ANXA1, which was accompanied by an increased nuclear translocation of NICD in KG1a and HL60 cells (Figures 2C and S3 and Table S1, Supporting Information).

To further localize the domain of ANXA1 in mediating this interaction, we expressed and purified a GST‐tagged fusion protein of full‐length ANXA1, N‐terminal, and C‐terminal domains of ANXA1, by using domain mapping (Figure S4A,B, Supporting Information). The protein binding experiments in vitro showed that Notch1 in the cell lysate could be pulled down by GST‐tagged ANXA1 (Figure S4C, Supporting Information). In addition, more Notch1 was pulled down in sgANXA1 cell line than control cell line (Figure S4C, Supporting Information), which was consistent with the results shown in Figure 1F. The results using the bio‐layer interferometry (BLI) analyses (Figure 2D) finally also showed that both full‐length and the C‐terminal domain of ANXA1 dose‐dependently increased the binding affinity with NICD (Figure 2E). Analyses of the binding kinetics yielded a K_D_ of 1.84 × 10^−5^ M for full‐length ANXA1 and 5.32 × 10^−6^ M for the C‐terminal domain of ANXA1 (Table S2, Supporting Information). These results provide further evidence for the direct interaction between NICD and the C‐terminal domain of ANXA1.

The ankyrin domain of NICD contains seven ankyrin repeats, in which the first ankyrin repeat is not involved in protein folding.^[^ ^18^ ^]^ To identify which region of the ankyrin domain‐mediated the interaction, we designed and synthesized peptides of 2–7 ankyrin repeats by solid‐phase peptide synthesis approach (Figures S15–S31, Supporting Information), including six linear peptides and six cyclic peptides, which were designed according to their crystal conformations (Figures 2F–H and S5A and Table S3, Supporting Information). The BLI analyses were used to further characterize the interactions between ANXA1 and synthesized peptides of the ankyrin domain of NICD. Only NICD‐5l peptide and NICD‐5c peptide increased the binding affinity with GST‐ANXA1 in a dose‐dependent manner (Figures 2I and S5B and Table S2, Supporting Information), suggesting that the 2029–2060 amino acid domain of NICD was the region mediating the interaction with ANXA1. Furthermore, 2050–2060 amino acid domain of NICD identified by chemical cross‐linking coupled with mass spectrometry may be more critical for interaction with the 214–228 amino acid domain of ANXA1 (Figure 2J). Consistent with this, ANXA1 (Δ214–228) failed to restore proliferative activity in sgANXA1 cells, further the interaction between NICD and ANXA1 was also lost due to the deletion of 2050–2060 amino acid domain of NICD (Figure S6, Supporting Information). Interestingly, both NICD‐5l peptide and NICD‐5c peptide significantly inhibited the proliferation of KG1a and HL60 cells in a dose‐dependent manner (Figure 2K). In contrast, other peptides for 2–7 ankyrin repeats of NICD appeared no obvious role in the proliferation of AML cells (Figure S7, Supporting Information). Surprisingly, conjugation of the cell‐penetrating peptide TAT (RKKRRQRRR, derived from HIV‐1) to NICD‐5l (named as TAT‐NICD‐5l) peptide greatly enhanced its osmotic capacity and cruelly destroyed the proliferation and colony forming ability of KG1a and HL60 cells, although TAT itself barely caused cell damage (Figures 2K and S8, Supporting Information). Overall, it is unexpected and surprising that ANXA1 was identified as a novel upstream regulator of Notch1 through direct interaction on cell membrane, and that the interaction between 2050 and 2060 amino acid domain of NICD and the C‐terminal 214–228 amino acid domain of ANXA1 inhibited the nuclear translocation of NICD.

### 2.3 Modulation of Notch1 Function by ANXA1 Through the Promoting of NICD Degradation via the Ubiquitin‐Proteasome System

**Figure 3:**
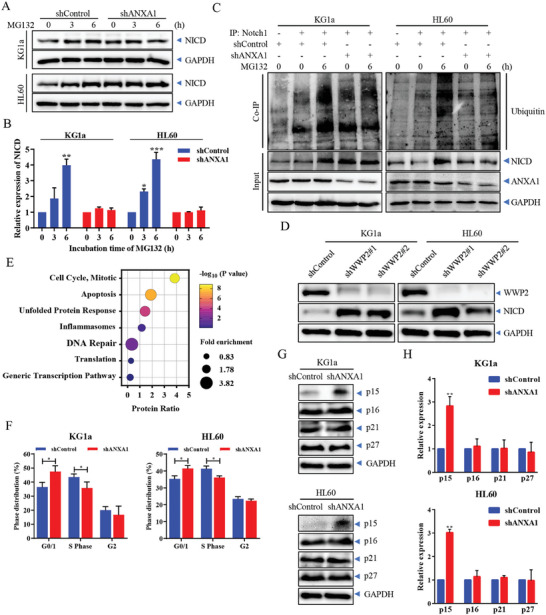
ANXA1 promotes the proteasome degradation of NICD and down‐regulates the expression level of p15. (A) Western blotting of NICD expression in KG1a and HL60 cells stably transfected with shANXA1 or shControl plasmids at 0, 3 and 6 h after MG132 treatment. (B) Statistical graph of NICD expression after MG132 treatment. Data are means ± s.e.m. of three independent experiments, one‐way ANOVA with Dunnett's test, compared with 0 h group, ^*^ *p* < 0.05, ^**^ *p* < 0.01, ^***^ *p* < 0.001. (C) Western blotting of NICD‐bound ubiquitin in immunoprecipitation complexes of KG1a‐ and HL60‐ shANXA1 or shControl cells treated with MG132 for 0 or 6 h. (D) Western blotting of WWP2 and NICD expression in KG1a and HL60 cells stably transfected with shWWP2 or shControl plasmids. (E) Biological pathway enrichment analysis of proteomic data from shANXA1 and shControl cells of KG1a. (F) Cell cycle analysis of KG1a and HL60 cells stably transfected with shANXA1 or shControl plasmids. Data are means ± s.e.m. of three independent experiments, *t*‐test, ^*^ *p* < 0.05. (G) Western blotting of cyclin‐dependent kinase inhibitors in KG1a and HL60 cells stably transfected with shANXA1 or shControl plasmids. (H) Statistical graph of cyclin‐dependent kinase inhibitors expression. Data are means ± s.e.m. of three independent experiments, *t*‐test, compared with shControl group, ^**^ *p* < 0.01.

To determine how ANXA1 inhibited the expression level of NICD, the proteasome inhibitor MG132 was used to block the proteasome degradation system. We found that pretreating AML cells with MG132 significantly reversed the ANXA1‐induced NICD degradation after 3 h of treatment in the control group, while MG132 minimally affected the expression level of NICD in the ANXA1 knockdown group (**Figure** **3**A,B). We further immunoprecipitated NICD and found that ubiquitin was bound to NICD significantly in the shControl cells after MG132 treatment rather than ANXA1 knockdown cells (Figure 3C). WWP2 is a recently discovered E3 ligase of NICD.^[^ ^19^ ^]^ Knocking down WWP2 can significantly restore the expression level of NICD in KG1a and HL60 cells (Figure 3D). The results showed that ANXA1 promoted the proteasome degradation of NICD by inducing ubiquitination, thereby inhibiting the activation of Notch1 signaling.

### 2.4 Ablation of the Notch1‐p15‐Mediated Tumor Suppression by ANXA1 Mediates the Proliferation of AML Cells

To identify the downstream molecular mechanisms involved in the direct interaction between ANXA1 and Notch1 to promote the proliferation of AML cells, we analyzed proteomics data as shown in Figure 1E. Biological pathway enrichment analysis of the proteomics data indicated that knockdown of ANXA1 in AML cells induced a significant enrichment in the progression of the cell cycle (Figure 3E). Consistent with the proteomics results, the results of flow cytometry validated the significant arrest of G0/G1 phase distribution in ANXA1 knockdown cells (Figures 3F and S9, Supporting Information), accompanied by a significant increase of the cyclin‐dependent kinase (CDK) inhibitor, p15, instead of p16, p21, and p27 (Figure 3G,H).

**Figure 4:**
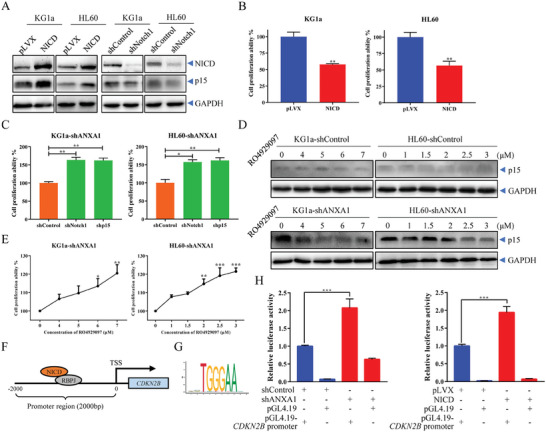
p15 is activated by NICD to directly inhibit cell proliferation. (A) Western blotting of Notch1 and p15 in KG1a and HL60 cells stably transfected with NICD over‐expression or shNotch1 plasmids. (B) The cell proliferation ability detected by cell counting after 96 h of culture of KG1a or HL60 cells stably transfected with NICD over‐expression or pLVX control plasmid. Data are means ± s.e.m. of three independent experiments, *t*‐test, ^*^ *p* < 0.05, ^**^ *p* < 0.01. (C) The cell proliferation ability detected by cell counting for KG1a‐shANXA1 and HL60‐shANXA1 cells transfected with shNotch1, shp15 or shControl plasmids. Data are means ± s.e.m. of three independent experiments, one‐way ANOVA with Dunnett's test, compared with shControl group, ^*^ *p* < 0.05, ^**^ *p* < 0.01. (D) Western blotting of p15 in KG1a and HL60 cells stably transfected with shANXA1 or shControl plasmids after RO4929097 treatment for 24 h. (E) The cell proliferation ability of KG1a‐shANXA1 and HL60‐shANXA1 cells was detected by cell counting after RO4929097 treatment for 24 h. Data are means ± s.e.m. of three independent experiments, one‐way ANOVA with Dunnett's test, compared with 0 µm group, ^*^ *p* < 0.05, ^**^ *p* < 0.01, ^***^ *p* < 0.001. (F) Schematic diagram of a predicted binding model of NICD/RBPJ complex on the promoter of *CDKN2B*. (G) The site with the highest sequence frequency among the transcription factor binding sites predicted by JASPAR. (H) Relative luciferase activities of *CDKN2B* promoter‐reporter constructs in KG1a cells stably transfected with shANXA1 or NICD over‐expression plasmids. Data are means ± s.e.m. of three independent experiments, *t*‐test, ^***^ *p* < 0.001.

To determine whether the increased expression of p15 induced by ANXA1 knockdown was mediated by NICD signals, we constructed stable NICD over‐expression and Notch1 knockdown cell lines by lentivirus infection. We found that p15 was up‐regulated or down‐regulated synchronously with the expression level of NICD (**Figure** **4**A), while over‐expression of NICD significantly decreased the cell proliferation of AML cells (Figure 4B). Furthermore, we knocked down the expression levels of Notch1 and p15 in the shANXA1 cell line (Figure S10, Supporting Information), which significantly recovered cell proliferation inhibited by knockdown of ANXA1 expression (Figure 4C). As the release of NICD depends on the cleavage of Notch1 by γ‐secretase.^[^ ^20^ ^]^ We blocked the cleavage by RO4929097,^[^ ^21^ ^]^ an inhibitor of γ‐secretase which inhibited the expression of p15 in KG1a and HL60 cells with ANXA1 knockdown (Figure 4D), thereby promoting the proliferation of AML cells (Figure 4E), showing that the transcription activation of p15 depended on the nuclear translocation of NICD.

NICD as an interacting protein of the transcription factor RBPJ, which indirectly regulates the activation promoter of target genes.^[^ ^7^ ^]^ To identify the potential binding site of the NICD/RBPJ complex interacting with the promoter region of *CDKN2B* (the gene encoding p15), the JASPAR database was used to predict transcription factor binding sites of RBPJ on the *CDKN2B* promoter, in which the TGGGAA site possessed the highest sequence frequency (Figure 4F,G). The luciferase reporter assay was subsequently used to determine the promotor gene activity of *CDKN2B*. We found that both knockdown of ANXA1 or overexpression of NICD significantly enhanced the activity of the *CDKN2B* promoter (Figure 4H). Together, the results identified *CDKN2B* as a novel downstream target gene regulated by NICD binding on the promotor of p15, which modulated cell proliferation.

### 2.5 A Mutual Exclusive Relation Exists Between ANXA1 and Notch1/p15 in Human Patients

**Figure 5:**
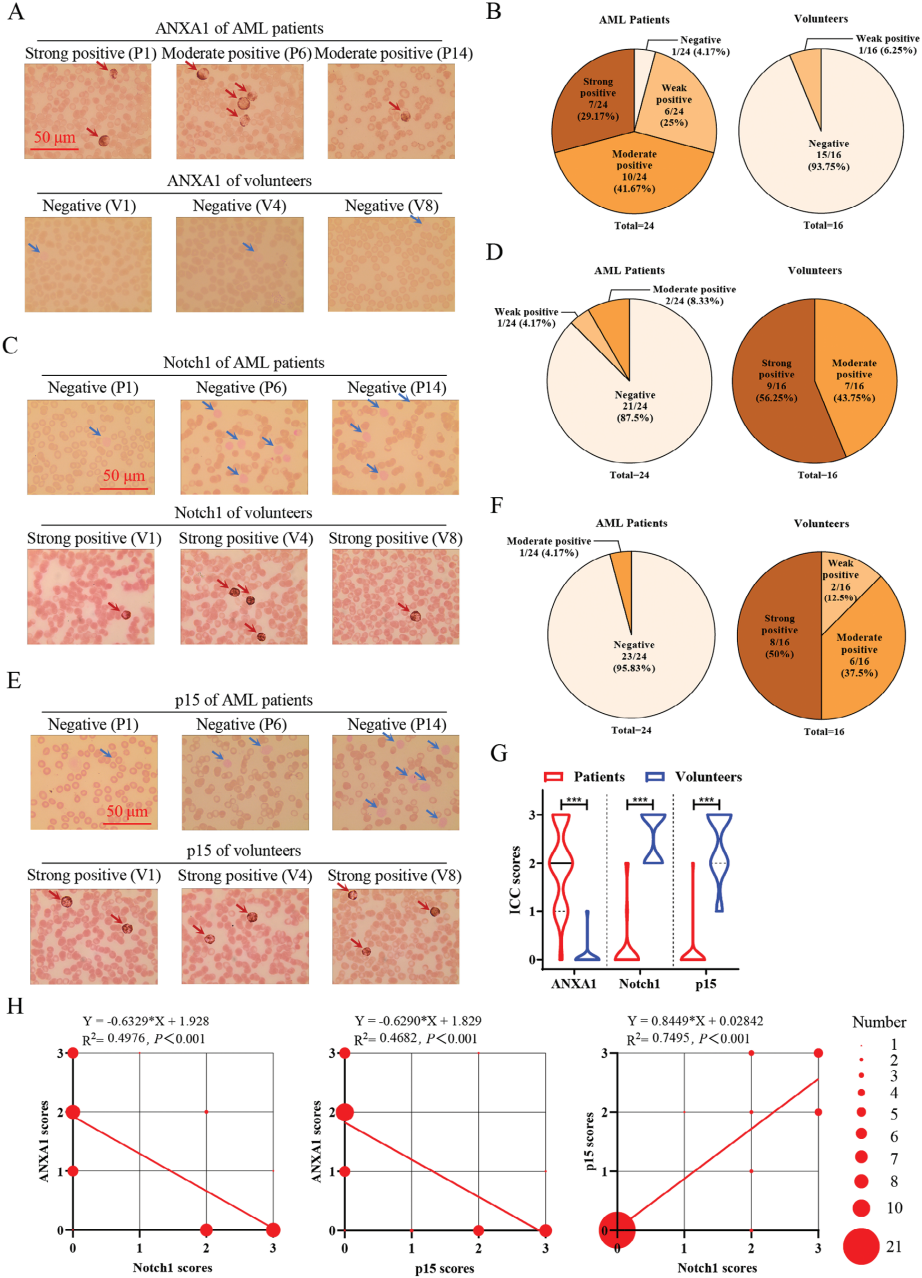
The expression level of ANXA1 was up‐regulated in peripheral blood leukocytes of AML patients, which was negatively correlated with the expression levels of Notch1 and p15. (A, C and E) Representative immunocytochemical images of ANXA1 (A), Notch1 (C) and p15 (E) ‐stained peripheral blood from AML patients and volunteers. The red and blue arrows indicate positive and negative stained white blood cells, respectively. Scale bar, 50 µm. (B, D, and F) Pie chart overview of ANXA1 (B), Notch1 (D) and p15 (F) immunostaining scores in AML samples (*n* = 24) and volunteers’ samples (*n* = 16). (G) Allred intensity score statistics for the immunocytochemical images of Figure S11 (Supporting Information). Student's *t*‐test, ^***^ *p* < 0.001. (H) Linear regression analyses among immunostaining intensity scores of ANXA1, Notch1 and p15, *p* < 0.001.

In order to further corroborate our mechanistic discovery in clinical AML patients, 24 AML patients and 16 volunteers (Table S4, Supporting Information) were enrolled to detect the expression levels of ANXA1 using immunocytochemical staining (**Figures** **5**A and S11A, Supporting Information). Positive immunostaining for ANXA1 protein was detected in 95.83% of AML patients, including 25% AML patients with weak expression, 41.67% with moderate expression, and 29.17% with strong expression, while there were just only 6.25% of volunteers with weak expression of ANXA1 (Figure 5B). Using the same patients’ samples, our results showed that Notch1 and p15 were significantly overexpressed in volunteers, but rarely expressed in AML patients (Figures 5C–F and S11B,C, Supporting Information). A combined autocorrelation method also demonstrated that ANXA1 was significantly overexpressed in AML patients, while Notch1 and p15 were significantly overexpressed in volunteers (Fisher's exact test, *p* < 0.0001, Table S5, Supporting Information). Combining the immunocytochemistry scores and the correlation indexes using Fisher's exact test, we found that the expression level of ANXA1 was significantly and negatively correlated with the expression levels of Notch1 and p15, respectively. While the expression level between Notch1 and p15 was positively correlated (Figure 5G,H; Table S5, Supporting Information).

### 2.6 Targeting the ANXA1‐Notch1 Signaling Provides an Effective Approach for Treatment of AML In Vivo and Ex Vivo

**Figure 6:**
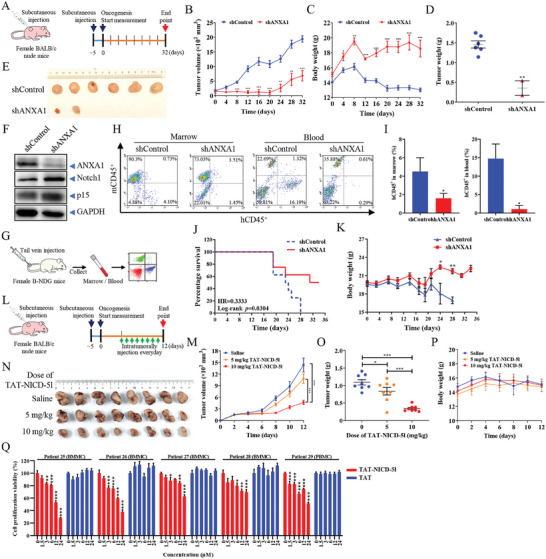
Targeting ANXA1 inhibits the proliferation of AML cells in vivo and ex vivo. (A) Schematic diagram of the course of the subcutaneous xenograft tumors experiment. (B) Statistical chart of tumor volume in the subcutaneous xenograft mice (*n* = 6). (C) Statistical chart of body weight of the subcutaneous xenograft mice (*n* = 6). (D) Statistical chart of the tumor weight of the subcutaneous xenograft mice. (E) Images of the tumors isolated from shControl and shANXA1 groups of the subcutaneous xenograft mice. (F) Western blotting of ANXA1, Notch1 and p15 in tumors isolated from subcutaneous xenograft mice. (G) Schematic diagram of the course of AML mouse model experiment. (H) Representative images of flow cytometry analysis of the transplantation levels in bone marrow and peripheral blood. (I) The proportion of hCD45^+^ cells in bone marrow and peripheral blood of AML mice. (J) Survival analysis of AML mice. Log‐rank test (*n* = 8), *p* = 0.0304. (K) Body weight analysis of AML mice (*n* = 8). (L) Schematic diagram of the course of the peptide therapy experiment in vivo. (M) Statistical chart of tumor volume in the subcutaneous xenograft mice (*n* = 8). (N) Images of the tumors isolated from the subcutaneous xenograft mice. (O) Statistical chart of the tumor weight of the subcutaneous xenograft mice. (P) Statistical chart of body weight of the subcutaneous xenograft mice (*n* = 8). (Q) The cell proliferation viability of bone marrow mononuclear cells and peripheral blood mononuclear cells from AML patients detected by luminescent assay after treatment with TAT‐ NICD‐5l peptide or TAT peptide for 24 h, at indicated concentration. Data in (B, C, D, I, K, M and O) are means ± s.e.m., *t*‐test, compared with control group, ^*^ *p* < 0.05, ^**^ *p* < 0.01, ^***^ *p* < 0.001. Data in (Q) are means ± s.e.m., one‐way ANOVA with Dunnett's test, compared with 0 µm group, ^*^ *p* < 0.05, ^**^ *p* < 0.01, ^***^ *p* < 0.001.

We further assessed the efficacy of ANXA1 in vivo. We have established an in vivo model of KG1a‐shControl and KG1a‐shANXA1 cells in BALB/c nude mice upon subcutaneous injection to form solid tumors that allow quantitative tumor volume measurements for the study of tumor growth in vivo. Tumors were clearly observed after 5 days of subcutaneous inoculation (defined as day 0) of 9 × 10^6^ cells in 5‐week‐old female BALB/c mice, then the tumor volumes were measured every 4 days for another 32 days (**Figure** **6**A). On day 4, all 6 mice in the shControl group developed tumors with the volume of 290.21 ± 39.25 mm^3^, while only 3 mice in the shANXA1 group developed tumors with the volume of 126.67 ± 31.98 mm^3^ (Figure 6B), and the body weight of mice in the two groups started to exhibit significant variances (Figure 6C). Subsequently, the tumors in the shControl group continued to grow and the body weight began to lose after day 8, while the tumors in the shANXA1 group grew significantly slower than those in the shControl group and were still present in only two mice after day 24, and the body weight of mice in the shANXA1 group was significantly increased (Figure 6B–E). On day 32, the tumor volume of shControl group was 1945.31 ± 110.11 mm^3^, and the tumor volume of shANXA1 group was 684.70 ± 179.60 mm^3^, we immediately executed all mice humanely (Figure 6B). Also, we found the protein abundance of ANXA1 in the tumors of shANXA1 group was significantly decreased, followed by an obvious increase of the expression levels of Notch1 and p15 protein (Figure 6F). Moreover, we also established an in vivo model in B‐NDG mice by tail vein injection of KG1a‐shControl and KG1a‐shANXA1 cells, respectively, in order to simulate human AML (Figure 6G). Compared with the shControl group, the transplantation levels of AML cells represented by hCD45^+^ cells in the bone marrow and peripheral blood were significantly reduced in shANXA1 cells (Figure 6H,I), and the survival of the mice was significantly prolonged (Figure 6J), along with the significantly increase of body weight in shANXA1 group after day 24 (Figure 6K). We analyzed the organs of the mice in each group and found that there was no significant difference in the size and weight of the heart, liver, spleen, lung, kidney, and brain (Figure S12A,B, Supporting Information), and the percentage of hCD45^+^ in the spleen also showed no significant difference (Figure S12C, Supporting Information). Therefore, these results showed that ANXA1 promoted the progression of human AML in mice in vivo.

Furthermore, we evaluated the drug sensitivity of TAT‐NICD‐5l peptide in vivo and ex vivo. We established the subcutaneous solid tumor model using KG1a cells to evaluate the therapeutic effect of the TAT‐NICD‐5l peptide in vivo (Figure 6L). After 8 days of continuous injections, the tumor volumes in TAT‐NICD‐5l peptide treatment groups with 5 mg kg^−1^ and 10 mg kg^−1^ were 1081.13 ± 120.58 mm^3^ and 465.86 ± 54.41 mm^3^, respectively, which was significantly reduced compared to control mice with 1434.54 ± 177.90 mm^3^ (Figure 6M). The isolated tumors also showed significant dose‐dependent efficacy after TAT‐NICD‐5l peptide treatment (Figure 6N,O). There was no significant difference in body weight among the different groups (Figure 6P), indicating that TAT‐NICD‐5l peptide has no potent toxicity at the indicated dosages in vivo. Ex vivo experiment also showed that TAT‐NICD‐5l peptide had the ability to significantly inhibit the cell proliferation of bone marrow mononuclear cells and peripheral blood mononuclear cells isolated from AML patients (Figure 6Q).

**Figure 7:**
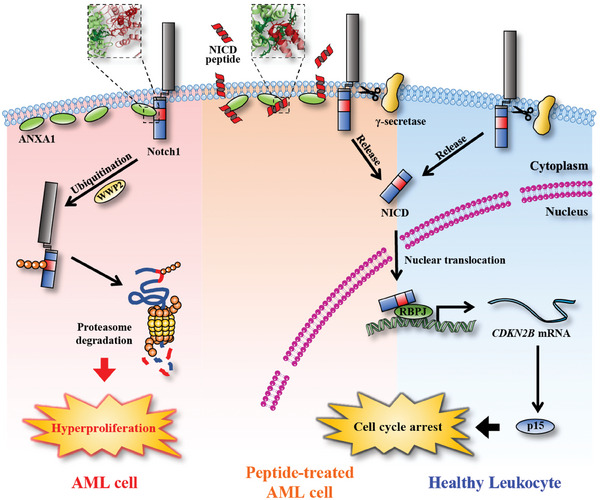
Schematic diagram of ANXA1‐Notch1‐p15 signal axis regulating hyperproliferation in human AML cells. In healthy leukocytes, NICD is cleaved by γ‐secretase and translocated to the nucleus, to activate the transcription of the *CDKN2B* gene, which expresses p15 protein to arrest the cell cycle in G_0_/G_1_ phase and then inhibits cell proliferation. In AML cells with abnormally high expression of ANXA1, 2050–2060 amino acids of NICD directly interacts with the C‐terminal 214–228 amino acid domain of ANXA1 on cell membrane. This interaction promotes ubiquitination of NICD by WWP2 and subsequent degradation of NICD by the proteasome, resultant of loss of p15 and hyperproliferation of AML cells. However, NICD inhibitory peptides competitively bind ANXA1 and restore Notch1 function.

Based on the above results in vitro, in vivo, and ex vivo, we proposed a novel molecular mechanism for Notch1 in regulating cell proliferation and a new therapeutic paradigm targeting the ANXA1‐Notch1 signaling of human AML as shown in **Figure** **7**.

## 3 Discussion

AML is one of the most common hematological malignancies resulting from genetic and epigenetic alterations that perturb key processes like self‐renewal, proliferation, and differentiation.^[^ ^22^ ^]^ In the United States, an estimated 20 800 cases of AML are expected in 2024, and 11 600 deaths due to AML.^[^ ^23^ ^]^ Chemotherapy and hematopoietic stem cell transplantation are still the main treatment methods for AML. Although, the targeted therapy and immunotherapy have improved the efficacy and prognosis of AML, but the mortality of patients is still high and the 5‐year survival rate is still less than 30%.^[^ ^24^ ^]^ Given the poor prognosis of AML, it is essential to improve our mechanistic understanding involved in the proliferation and development of AML.

Notch signaling is evolutionarily conserved from *Drosophila* to *Homo sapiens*, which has been extensively investigated as a regulator of cell fate decisions in a variety of organisms and tissues. Dysregulation of the Notch signaling is involved in many hematological and solid malignancies.^[^ ^5^, ^25^ ^]^ Notch1 is a well‐characterized oncoprotein in T‐ALL, while Notch1 is believed to be a tumor suppressor in AML.^[^ ^5^, ^26^ ^]^ In T‐ALL, there are several reports proposing different molecular mechanisms involved in the leukemogenesis of Notch1. The DEP‐domain containing mTOR‐interacting protein is a direct Notch1 target, which promotes cell proliferation and survival in T‐cell leukemia.^[^ ^27^ ^]^ A Notch1‐driven MYC enhancer promotes oncogenesis and highlights the importance of the Notch1‐MYC regulatory axis as a therapeutic target for T‐ALL patients.^[^ ^28^ ^]^ Notch1 regulates the expression of PTEN and the activity of the PI3K‐AKT signaling pathway of the leukemogenic program in T‐ALL.^[^ ^29^ ^]^ Similarly, NF‐κB is one of the major mediators of Notch1‐induced transformation, and the NF‐κB pathway is a potential target of future therapies for T‐ALL patients.^[^ ^30^ ^]^ In contrast with the comprehensive research of Notch1 in T‐ALL, the molecular mechanisms of Notch1 in inhibiting cell proliferation in myeloid leukemia remain incompletely understood. Moreover, little is known of the regulatory mechanisms involved in the upstream control of Notch1 function and activity.

The expression level and function of ANXA1 vary according to tumor type. Previous reports showed the contrasting roles of ANXA1 as a tumor suppressor protein or an oncogenic protein in different tumors/cancers, or even in the same cancer types.^[^ ^15^ ^]^ In breast cancer cells, ANXA1 promotes metastasis and facilitates an epithelial‐mesenchymal transition‐like switch by enhancing TGF‐β/Smad signaling.^[^ ^11c^ ^]^ In head and neck cancers, downregulation of ANXA1 results in phosphorylation of the epidermal growth factor receptor (EGFR) and activation of PI3K‐AKT signaling, in addition to reduced exosome production and release of phosphorylated EGFR, thereby inhibiting tumor progression.^[^ ^31^ ^]^ However, the expression level, function, and mechanisms of ANXA1 in myeloid leukemia are still unclear. At the beginning of the present study, we aimed to identify the role and mechanisms of ANXA1 during AML progression. We found that ANXA1 was overexpressed in AML patients, and that ANXA1 promoted the proliferation of AML cells. However, we excluded the classical mechanisms of ANXA1 involved in cancer progression reported in previous studies, such as the signal pathways of MAPK, NF‐κB, Hippo, and TGF‐β (Figure S13, Supporting Information). Unexpectedly and surprisingly, we first identified ANXA1 as a novel upstream regulator of Notch1 by its direct protein‐protein interaction on cell membranes, to promote the degradation of Notch1 through the ubiquitin‐proteasome pathway. The inactivation of Notch1 has been considered to be associated with p53 mutation.^[^ ^32^ ^]^ However, in our present research, KG1a cells and HL60 cells are TP53 mutant and TP53 deficient, respectively, thus our study identified a novel mechanism of inactivation of Notch1 through the direct regulation by ANXA1. Phospholipids are present in high levels in p53 mutant cells, which may also influence the effect of ANXA1 through their interaction on the membrane.^[^ ^33^ ^]^ These raise the question of whether ANXA1 is a biomarker of p53 mutant malignancies.

The N‐terminal domain of ANXA1 serves as a ligand of formyl peptide receptor (FPR) and triggers different signaling pathways in a dose‐dependent manner in human neutrophils, and the activity of N‐terminal ANXA1 peptides (Ac2‐26 and Ac2‐12) can be blocked by the FPR antagonists, Boc1 and Boc2.^[^ ^34^ ^]^ However, our present findings showed that neither the FPR antagonist nor the N‐terminal ANXA1 peptide Ac2‐26 affected the proliferation of AML cells (Figure S14, Supporting Information), indicating that endogenous ANXA1 exerted the proliferative effect through the full‐length form or C‐terminal domain (from Pro^44^ to Gly^344^) in AML cells, mediated by somehow mechanisms instead of a ligand of FPR.^[^ ^11b^ ^]^ Furthermore, our results showed that ANXA1‐Notch1 interaction contributed to the hyperproliferative effect of AML cells. The ANXA1‐Notch1 interaction sites were located at the C‐terminal domain of ANXA1, inhibiting Notch1 activation by γ‐secretase. According to the crystalline structure, we designed and synthesized 12 functional peptides of the NICD ankyrin domain to characterize the peptide‐ANXA1 interactions using the BLI analyses, which was further reconfirmed by cross‐linking mass spectrometry experiments. Our mass spectrometry results showed that the 2,050–2,060 amino acid domain of NICD directly interacted with the C‐terminal 214–228 amino acid domain of ANXA1. Furthermore, peptides designed on the basis of protein‐protein interface inhibited the proliferation of AML cell lines, bone marrow mononuclear cells and peripheral blood mononuclear cells of AML patients in a dose‐dependent manner.

Many target gene systems can be activated by Notch1, such as transcriptional repressors (the *HES1* and *HEY1* families), oncogenic pathways (*NF‐κB* and *MYC*), cell cycle progression pathways (*CCND1/3*), inhibition of apoptosis (*BCL2*), and cell migration/metastasis (*CCR7*), depending on the cell type and ligand‐receptor interactions at the cell surface.^[^ ^5^, ^25^ ^]^ In the present study, cyclin‐dependent kinase inhibitor‐2B (*CDKN2B*, also known as p15) was identified as a novel target of Notch1 by binding to the promotor of p15, to arrest cell cycle progression and inducing the suppression of proliferation when ANXA1 was knocked‐down in AML cells. We also identified a mutually exclusive relation that existed between ANXA1 and Notch1/p15 in AML patient samples, which further confirmed that ANXA1 promoted the proliferation of AML cells through functional suppression of Notch1 and p15.

In conclusion, we discovered a mutually exclusive relationship between ANXA1 and Notch1/p15 in the same AML patients. Furthermore, endogenous ANXA1 promoted the proliferation of AML cells in vitro and in vivo through the ablation of Notch1‐p15‐mediated tumor suppression. In detail, we revealed that the C‐terminal 214–228 amino acid domain of ANXA1 interacted directly with the amino acid residues 2050–2060 of NICD to inhibit the expression of p15. On the basis of these novel mechanistic findings, we reasonably speculate that targeting ANXA1 would provide an effective approach for the treatment of AML. In support of this new therapeutic paradigm, we provided proof‐of‐concept data by antagonizing ANXA1 using NICD inhibitory peptides in vitro, in vivo and ex vivo. Thus, our present results provide new insights into the development of novel AML drugs, such as stapled peptides, peptidomimetics, small molecules, and antibodies, to treat AML based on the direct interaction of ANXA1 and Notch1 on the cell membrane.

## 4 Experimental Section

### Cell Lines and Peptides

Human acute myeloid leukemia KG1a and HL60 cell lines were obtained from the First Affiliated Hospital, Zhejiang University School of Medicine. The peptides were synthesized on Wang resin (Sunresin, Xi'an, China) by the solid phase technique. Details of these procedures are described in the Materials and Methods (Supporting Information).

### Plasmid Construction and Protein Expression

The insertion sequences, vectors, and restriction sites of all plasmids are provided in the Materials and Methods (Supporting Information). 293T cells were used to package lentivirus. BL21 chemical‐competent *E. coli* cells were used to induce the expression of targeted proteins.

### Protein‐Protein Interaction

AlphaFold2 and ClusPro servers were used to dock NICD and ANXA1.^[^ ^35^ ^]^ The predictions were tested by GST‐pull down assay, bio‐layer interferometry (BLI) analysis, and chemical cross‐linking coupled with mass spectrometry. Details of these procedures are described in Materials and Methods (Supporting Information).

### Mouse Models

The animal experiment protocol was performed following the guide for the Care and Use of Laboratory Animals. Five‐week‐old female BALB/c nude mice with similar body weight were equally divided into two groups and subcutaneously inoculated with KG1a‐shControl or KG1a‐shANXA1 cells (9 × 10^6^ cells per mouse) into the posterior back regions. The tumor volumes and body weights in each group were recorded at regular intervals. Six‐week‐old female B‐NDG mice were inoculated with KG1a‐shControl or KG1a‐shANXA1 cells (3 × 10^6^ cells per mouse) via tail‐vein injection. Peripheral blood white cells, blast cells, and spleen cells were collected and stained with FITC‐hCD45 and PE‐mCD45 antibodies to identify the proportion of tumor cells. For peptide therapy experiment in vivo, TAT‐NICD‐5l peptide was intratumorally injected once a day after tumors reached 150 mm^3^. Details of these procedures are described in the Materials and Methods (Supporting Information).

### Statistical Analysis

All statistical data are presented as the means ± standard error of the mean (s.e.m.). Student's *t*‐test, one‐way ANOVA, log‐rank test, simple linear regression analysis, and Fisher's exact test were used for statistical analyses. *p* values of ^*^ < 0.05, ^**^ < 0.01, and ^***^ < 0.001 were considered significant. Details of these procedures are described in the Materials and Methods (Supporting Information).

### Study Approval

Clinical sample experiments were approved by the Ethics Committee of The First Affiliated Hospital of Zhejiang University School of Medicine (No. 2021‐601). BALB/c nude and B‐NDG mice were used for animal work, which was approved by the ethics committee of animal experiments at Zhejiang Sci‐Tech University (No. 20210908‐1).

## Conflict of Interest

The authors declare no conflict of interest.

## Author Contributions

G.S., X.W., Y.Z., and J.M. contributed equally to this work. C.F. served as the lead contact for conceptualization, methodology, supervision, project administration, funding acquisition, and writing—review & editing. Y.C. and J.K. contributed to writing—review & editing. G.S. handled investigation, validation, data curation, funding acquisition, and wrote the original draft. H.W. provided resources, investigation, and validation. X.W., Y.Z., J.M., L.W., Z.Y., Z.S., S.Z., H.W., Y.L., H.H., J.L., T.Z., B.Y., N.W., T.C., X.G., and Y.J. were involved in investigation and validation.

## Supporting information



Supporting Information

## Data Availability

The data that support the findings of this study are available from the corresponding author upon reasonable request. The raw mass spectrometry proteomics data have been deposited to the ProteomeXchange Consortium (PXD ID: PXD056593).
