# Supplementary material for: Identification of ANXA1 as a Novel Upstream Negative Regulator of Notch1 Function in AML
Source: Adv Sci (Weinh). 2024 Oct 24;11(48):2409726. doi: 10.1002/advs.202409726 (PMC11672316; doi:10.1002/advs.202409726)
Supplement: Supplementary file 1 — Supporting Information [file ADVS-11-2409726-s001.docx]

Supporting Information

**Identification of ANXA1 as A Novel Upstream Negative Regulator of Notch1 Function in AML**

Gang Shao, Xi Wang, Yiting Zheng, Junjie Ma, Lei Wang, Zhibin Yan, Zeyu Sun, Shuyuan Zhang, Hongzhang Wu, Yudie Lv, Hemiao Huang, Jianhu Li, Tianyi Zhu, Bing Yang, Nanxi Wang, Tao Chen, Xuancheng Guo, Yuanting Jin, Jian Kang, Huafeng Wang, Yihai Cao*, Caiyun Fu*

**This file includes:**

Materials and Methods

Figures S1 to S31

Tables S1 to S6

Supporting references

**Contents**

**1. Materials and Methods 1**

1.1. Data mining 1

1.2. Proteomics and gene enrichment analyses 1

1.3. Clinical samples and mouse models 1

1.4. Immunofluorescence and immunocytochemistry 3

1.5. Cell culture and drugs 4

1.6. Plasmid construction and transfection 4

1.7. Construction of stable cell lines 5

1.8. Protein expression and purification 5

1.9. Peptide design and synthesis 6

1.10. Peptide uptake assay 7

1.11. Cell proliferation viability assay 7

1.12. Colony formation assay 8

1.13. Flow cytometric analysis 8

1.14. Western blotting 8

1.15. Protein-protein docking prediction 9

1.16. Protein-protein interaction assays 9

1.17. Chemical cross-linking coupled with mass spectrometry assay 10

1.18. Luciferase assay 11

1.19. Statistical analysis 12

**2. Supplementary Figures 13**

Figure S1. mRNA expression levels of AML biomarkers in AML patients 13

Figure S2. Verification of the construction effect of ANXA1 knockdown and overexpression cells 14

Figure S3. Profile analyses of the regions of interest in colocalization images 15

Figure S4. GST pull down analysis of the interaction between ANXA1 and Notch1 16

Figure S5. 3D structural models of the designed peptides mimicking the ankyrin domain of NICD and BLI analyses between ANXA1 and indicated peptides 17

Figure S6. ANXA1 (214−228) and NICD (2,050−2,060) mediate the protein-protein interaction 18

Figure S7. Effect of NICD peptides on AML cell proliferation 19

Figure S8. Effects of TAT-NICD-5l peptide on cell penetration and colony forming ability 20

Figure S9. Representative cycle distribution images of KG1a and HL60 cells stably transfected with shANXA1 or shControl plasmids 21

Figure S10. Verification of the effect of Notch1 and p15 knockdown on the expression of p15 in KG1a- and HL60- shANXA1 cells 22

Figure S11. The expression level of ANXA1, Notch1 and p15 in peripheral blood leukocytes of AML patients 23

Figure S12. Effects of AML mouse models constructed by KG1a-shControl and KG1a-shANXA1 cells on viscera and brain 25

Figure S13. Protein expression levels in several classical signal pathways after knockdown of ANXA1 in KG1a cells 26

Figure S14. Effects of blocking or activation of formyl peptide receptor (FPR) on the proliferation of AML cells 27

Figure S15. Synthesis process of NICD-2l peptide 28

Figure S16. Synthesis process of NICD-2c peptide 29

Figure S17. Synthesis process of NICD-3l peptide 30

Figure S18. Synthesis process of NICD-3c peptide 31

Figure S19. Synthesis process of NICD-4l peptide 32

Figure S20. Synthesis process of NICD-4c peptide 33

Figure S21. Synthesis process of NICD-5l peptide 34

Figure S22. Synthesis process of NICD-5c peptide 35

Figure S23. Synthesis process of NICD-6l peptide 36

Figure S24. Synthesis process of NICD-6c peptide 37

Figure S25. Synthesis process of NICD-7l peptide 38

Figure S26. Synthesis process of NICD-7c peptide 39

Figure S27. Synthesis process of TAT peptide 40

Figure S28. Synthesis process of TAT-NICD-5l peptide 41

Figure S29. Synthesis process of FITC-NICD-5l peptide 42

Figure S30. Synthesis process of FITC-NICD-5c peptide 43

Figure S31. Synthesis process of FITC-TAT-NICD-5l peptide 44

**3. Supplementary Tables** **45**

Table S1. Manders overlap coefficient of the regions of interest in colocalization images 45

Table S2. Kinetic parameters for BLI analyses 46

Table S3. The information on the location and sequences of designed NICD peptides 47

Table S4. The information of acute myeloid leukemia patients 48

Table S5. Contingency table and Fisher's exact test of protein expression and sample source 49

Table S6. The information of antibodies 50

**Supporting references 51**

**1. Materials and Methods**

**1.1. Data mining**

Gene expression profiling (GEP) data for AML and non-leukemia patients were partially obtained from the GEO database (www.ncbi.nlm.nih.gov/geo). The probe ids were converted to gene symbols by GPL96 and GPL7473. GEP of TCGA dataset (*NEJM* 2013) was obtained from cBioportal database (www.cbioportal.org). Data on FAB classification and overall survival for AML patients were downloaded from cBioportal (www.cbioportal.org).

The gene coding sequences of ANXA1 (Gene ID: 301), Notch1 (Gene ID: 4851), p15 (*CDKN2B*, Gene ID: 1030) and the promoter sequence of p15 (-2000 to -1 nt) were obtained from NCBI. The binding sites of the transcription factor RBPJ (Matrix ID: MA1116.1) in *CDKN2B* promoter were predicted by JASPAR (jaspar.genereg.net) with a relative profile score threshold of 80%.

**1.2. Proteomics and gene enrichment analyses**

ANXA1 knockdown and control cells in the logarithmic growth phase were collected and lysed in RIPA lysis buffer (1% Triton X-100, 1% sodium deoxycholate and 0.1% SDS). Protein samples are digested into peptides after denaturation, reduction, and alkylation. The peptides of different samples were labeled with TMT10plex^TM^ Isobaric Label kit (Thermo Fisher Scientific), and then combined into one sample. The combined sample was fractionated using HPRP liquid chromatography, and then subjected to desalting treatment. The samples were detected by LC-MS/MS (nanoACQUITY UPLC, Waters and Q-Exactive HF-X, Thermo Fisher Scientific).

The gene enrichment was based on the proteomics data and analyzed by MaxQuant (version 1.6.5.0), UniProtKB database (version 2018.11) and GSEA (version 4.1).

**1.3. Clinical samples and mouse models**

Blood samples of 16 volunteers and 25 AML patients and bone marrow samples of 4 AML patients were obtained from the First Affiliated Hospital, Zhejiang University School of Medicine. All AML patients provided informed written consent for specimen collection. All the experiments were approved by the Ethics Committee of The First Affiliated Hospital of Zhejiang University School of Medicine (No. 2021-601).

One blood sample and four bone marrow samples were centrifuged to remove the serum and carefully layered using the Ficoll gradient solution. The mononuclear cell layer was transferred and cultured in IMDM medium (Gibco) with 20% fetal bovine serum (Gibco) and 1% penicillin-streptomycin (Gibco) at 37℃ in a humidified 5% CO_2_ incubator for the next luminescent cell viability assay. The remaining blood samples of 16 volunteers and 24 AML patients were smeared on glass slides and examined by immunocytochemistry.

Animal work trials were approved by the ethics committee of animal experiments at Zhejiang Sci-Tech University (No. 20210908-1). For *in vivo* assay, BALB/c nude and NOD-*Prkdc^scid^* *IL2rg^tm1^*/Bcgen (B-NDG) mice were purchased from Shanghai SLAC Laboratory Animal Company and Biocytogen, respectively. 5-week-old female BALB/c nude mice were equally divided into two groups according to similar body weight and subcutaneously inoculated with KG1a-shControl or KG1a-shANXA1 cells (9×10^6^ cells per mouse) into the posterior back regions. Once the tumor was formed, tumor volume was calculated using the formula: volume = length × (width)^2^ / 2. When tumors reached 100–150 mm^3^ (day 0), the tumor volumes and body weights in each group were recorded once every 4 days for another 32 days. Then the tumors were isolated, weighed, and examined by western blotting. For peptide therapy experiment *in vivo*, subcutaneous solid tumor of KG1a cells was formed with the same method as above. When tumors reached about 150 mm^3^, mice were randomly grouped and started TAT-NICD-5l peptide therapy with intratumoral injection once a day. The tumor volumes and body weights in each group were recorded once every 2 days. Moreover, KG1a-shControl or KG1a-shANXA1 cells (3×10^6^ cells per mouse) were transplanted into 6-week-old female B-NDG mice via tail-vein injection. Mice were sacrificed when terminally ill, and the visceral organs, brains and blast cells from bone marrow were collected. For hCD45^+^ staining, mouse peripheral blood was collected periodically by submandibular bleeding. Blood was stained with FITC-conjugated human CD45 and PE-conjugated mouse CD45 antibodies **(Table S6, Supporting Information)**. The red blood cells (RBC) were lysed by using RBC lysis buffer (Solarbio), the collected blast cells and spleen cells were treated as described above. The stained cells were measured by flow cytometry using Cytomics FC 500 (Beckman Coulter).

**1.4. Immunofluorescence and immunocytochemistry**

Cells were cultured on glass slides precoated with polylysine (Sigma-Aldrich) and fixed with 4% paraformaldehyde (Biosharp) for 20 min. The slides were then permeabilized with 0.25% Triton X-100 (Huadong Medicine) and blocked with 5% BSA (Solarbio).

For immunofluorescence assay, the samples were incubated with the ANXA1 and Notch1 primary antibodies overnight followed by the FITC labeled secondary antibody and rhodmine labeled secondary antibody **(Table S6, Supporting Information)** for 1 hour. The slides were dyed with DAPI solution (Biosharp) and mounted with a drop of anti-fluorescence quenching agent (Beyotime) before detecting with a laser scanning confocal microscope (Nikon) in a dark room. Coloc 2 and Plot Profile of ImageJ 1.53 were used for quantitative analyses.

For immunocytochemistry assay, the samples were incubated with the ANXA1, Notch1 or p15 primary antibody overnight and the secondary antibody **(Table S6, Supporting Information)** for 1 hour. The slides were dyed with DAB (Servicebio) and nuclear fast red (Cas# 6409-77-4) solution, then detected with a 100× oil-immersion microscope objective lens (Olympus). Expression levels were evaluated semi-quantitatively using the Allred intensity score, the average intensity of positive-staining tumor cells was divided into 4 levels (0: negative, 1: weak positive, 2: moderate positive and 3: strong positive).

**1.5. Cell culture and drugs**

Human acute myeloid leukemia KG1a and HL60 cell lines were obtained from the First Affiliated Hospital, Zhejiang University School of Medicine. Cells were regularly screened for mycoplasma contamination using mycoplasma stain assay kit (Beyotime), and authenticated by short tandem repeat (STR) analysis before these studies. Cells were cultured in RPMI 1640 medium (Gibco) with 10% fetal bovine serum (MeisenCTCC) at 37°C in a humidified 5% CO_2_ incubator.

Puromycin (Solarbio) was dissolved in the medium at a store concentration of 1 mg/mL for cell screening. MG-132 (Solarbio) was dissolved in dimethyl sulfoxide (DMSO, Sigma-Aldrich) at 2 mmol/L for inhibiting proteasome. RO4929097 (MedChemExpress) was dissolved in DMSO at 5 mmol L^-1^ for inhibiting γ-secretase. For *in vivo* studies, cyclophosphamide (Huamaike) was dissolved in saline at 10 mg/mL for inhibiting immune responses.

**1.6. Plasmid construction and transfection**

All plasmids used in this study were assembled by standard cloning methods and confirmed by DNA sequencing. For knocking down gene expression, the ANXA1 siRNA sequences (siRNA-1: 5'-GCCTTGTATGAAGCAGGAGAA-3', siRNA-2: 5'-GCATTCTATCAGAAGATGTAT-3') and WWP2 siRNA sequences (siRNA-1: 5'-CAGGATGGGAGATGAAATACA-3', siRNA-2: 5'-AGCACAGAGTCATTTAGATTT-3') were cloned into the pGLV3 vector using *BamH I* and *EcoR I* sites. Notch1 siRNA sequence (5'-GGAGCATGTGTAACATCAACA-3') and p15 siRNA sequence (5'-ACTAGTGGAGAAGGTGCGACA-3') were cloned into the pLKO.1 vector using *EcoR I* and *Age I* sites, respectively. ANXA1 sgRNA sequences (sgRNA-1: 5'-CAAACTGTGAAGTCATCCAA-3', sgRNA-2: 5'-GACTGTGAAGTCATCCAAAGG-3', sgRNA-3: 5'-GATGCAAGGCAGCGACATCCG-3') were cloned into lentiCRISPR-v2-puro (addgene Cat# 98290) linearized with *BsmB I* site. For eukaryotic overexpression of the genes, ANXA1 coding sequence was assembled into pCDH vector using *BamH I* and *Not I* sites and codon-optimized NICD coding sequence was assembled into pLVX vector using the *Xho I* and *Xba I* sites. For prokaryotic expression of the genes, coding sequences of GST-tagged ANXA1 full-length, C-terminal and N-terminal domains were assembled into pGEX4T1 vector and the coding sequence of His-Tagged NICD was assembled into pET24a vector, all using *BamH I* and *Xho I* sites. For detecting the transcriptional activity of p15, the promoter sequence of *CDKN2B* (-2,000 to -1 nt) was cloned into the pGL4.19 vector using *Kpn I* and *Xho I* sites.

Lipofectamine 2000 (Invitrogen) liposomes were used to encapsulate the constructed plasmids and then transfect KG1a and HL60 cells to perform transient gene knockdown and overexpression.

**1.7. Construction of stable cell lines**

For lentivirus packaging, HEK-293T cells were seeded in a 100 mm dish and transfected with pMD2.G, psPAX2 and constructed plasmids mixed in a ratio of 1:3:4 using Polyethylenimine Linear MW40000 (PEI 40000, Yeasen). After 8 hours, the medium was removed and complete medium was added for another 48 hours. The lentivirus in the supernatant was precipitated after incubation with PEG8000/NaCl solution and centrifugation, then redissolved in the RPMI 1640 medium.

For lentiviral infection, KG1a and HL60 cells were seeded in a 48-well plate, incubated in concentrated lentivirus and 10 μg mL^-1^ polybrene (Yeasen) for 45 min, then centrifuged in a horizontal centrifuge at 750 ×g for 2 hours, incubated in an incubator for 1 hour, and finally replaced with fresh RPMI 1640 medium. Stable cell lines were obtained by treatment of 1 μg mL^-1^ puromycin.

**1.8. Protein expression and purification**

BL21 chemical competent *E.coli* cells (New England Biolabs) transformed with pGEX4T1-GST-ANXA1, pGEX4T1-GST-ANXA1-C, pGEX4T1-GST-ANXA1-N, or pET24a-His Tag-NICD, respectively, and cultured overnight at 37°C containing 100 μg/ml ampicillin for pGEX4T1 and 50 μg mL^-1^ kanamycin for pET24a. A single colony was picked up and grown in 4 mL LB overnight at 37°C. The culture was diluted into 500 mL LB and incubated at 37°C until OD_600nm_ reached 0.4, and then the IPTG at 0.5 mM was added to induce the expression of targeted proteins overnight. The cell pellet was resuspended in lysis buffer and sonicated with a Sonic Dismembrator (Thermo Fisher Scientific), and then centrifuged at 12000 rpm for 20 min at 4°C. The supernatant was collected and purified with GST resin (Solarbio) or Ni-IDA resin (GE Healthcare) according to the instruction.

**1.9. Peptide** **design and synthesis**

The ankyrin domain of NICD contains seven ankyrin repeats. The linear peptides and cyclic peptides were designed from the 6 repeated structures according to the sequences (NCBI Gene ID: 4851) and high-resolution crystal structure (PDB: 1YYH) of 2-7 ankyrin repeats of NICD. Two cysteines were introduced into the linear peptides to substitute two amino acids with side chains next to one other, resulting in cyclic peptides that match each repeated structure of NICD. A TAT peptide (RKKRRQRRR) derived from HIV-1 was conjugated to NICD-5l peptide to elevate the transmembrane ability. FITC was conjugated to the peptides in order to allow the location of the peptides to be visualized under fluorescence microscopy. The designed peptide sequences are listed in the **Table S3 (Supporting Information)** and **Figure 2H**.

NICD peptides were synthesized on Wang resin (Sunresin) by the solid phase technique according to the designed sequences. The atomic accumulation of the peptides was verified by using electrospray ionization mass spectrometry (ESI-MS, LCMS-2020, Shimadzu) and the purity was detected by reversed-phase high-performance liquid chromatography (RP-HPLC, Prominence LC-20AT, Shimadzu). Detailed flow charts are shown in the **Table S15–31 (Supporting Information)**.

**1.10. Peptide uptake assay**

The FITC-NICD-5c peptide or FITC-TAT-NICD-5l peptide was added to KG1a and HL60 cells for 24 hours, and then the cells were stained with Hoechst33342 for 20 min. The fluorescence photographs were taken using a fluorescence microscope (EVOS M7000, Thermo Fisher Scientific) in a dark room.

**1.11. Cell proliferation viability assay**

For the cell counting assay, transfected cell lines or parental cells were seeded in a cell culture plate at the same density and cultured with RPMI-1640 medium, which sometimes added drugs. The number of cells was counted by a cell counter (Countstar BioTech IC 1000, Countstar) after indicated time.

For Methylthiazolyldiphenyl-tetrazolium bromide (MTT) assay, cells were seeded in a 96-well plate and incubated with drugs or peptides. At the indicated time point, each well was added with 10 μL MTT solution (5 mg mL^-1^, Sigma-Aldrich) and then incubated at 37°C in 5% CO_2_ for 4 hours. The medium was replaced by DMSO with 150 μL to dissolve the formazan crystals. The absorbance was measured at 490 nm using a microplate reader (Varioskan Flash, Thermo Fisher Scientific).

For Cell Counting Kit-8 (CCK8) assay, cells were seeded in a cell culture plate and incubated with peptides. At the indicated time point, CCK8 solution (Beyotime) was added to wells for a final concentration of 10% and then incubated at 37°C in 5% CO_2_. The absorbance was measured once an hour at 450 nm using a microplate reader (Varioskan Flash, Thermo Fisher Scientific).

For luminescent cell viability assay, isolated bone marrow mononuclear cells and peripheral blood mononuclear cells were seeded on an opaque-walled 96-well plate and incubated with peptides for 24 hours. CellTiter-Lumi detection reagent (Beyotime) was added into each well and measured using a microplate reader (Varioskan Flash, Thermo Fisher Scientific).

**1.12. Colony formation assay**

To evaluate the self-renewal ability of tumor stem cells, cells were seeded in 0.36% low-melting-point agarose on top of a base layer containing 0.8% agarose in the 6-well plate. Liquid media with TAT-NICD-5l peptide was added on top of the cell layer and replaced twice a week. The plate was incubated at 37C in 5% CO_2_ for 14 days. At the end of the experiment, colonies were stained with 1 mg/mL nitro blue tetrazolium chloride and counted.

**1.13. Flow cytometric analysis**

Cells were collected and fixed in 75% ice-cold ethanol for 24 hours and then stained with PI/RNase for 30 min in the dark according to the instruction of the cell cycle kit (KeyGen Biotech) before analysis by flow cytometry (CyFlow Cube 6, Sysmex). The results were analyzed by NovoExpress software (Agilent).

**1.14. Western blotting**

Cells were lysed in a lysis buffer containing 0.5 mM EDTA, 20 mM HEPES and 2% SDS. Samples mixed with SDS-gel loading buffer were loaded onto SDS-PAGE gel and transferred onto polyvinylidene fluoride (PVDF) microporous membranes (Millipore). Membranes were blocked with 5% nonfat milk in TBST (20 mM Tris-HCl, 150 mM NaCl, 0.1% Tween-20 with pH 7.5) for 2 hours and incubated with primary antibodies **(Table S6, Supporting Information)** at 4°C overnight. The membranes were incubated with a secondary antibody **(Table S6, Supporting Information)** followed by detection in an imaging system (ChampChemi 910, SageCreation) using the ECL reagent solution (SageCreation).

**1.15. Protein-protein docking prediction**

For rigid body docking prediction, ClusPro server (cluspro.bu.edu) was used to dock NICD ankyrin domain (PDB: 1YYH) and ANXA1 (PDB: 1MCX), and these 3D structure models were obtained from RCSB Protein Data Bank (www.rcsb.org).^[1]^ The prediction results were visualized using PyMOL 2.4.0.

For highly accurate docking prediction, an online version of AlphaFold2 (colab.research.google.com/github/sokrypton/ColabFold/blob/main/AlphaFold2.ipynb) was used to dock NICD and ANXA1.^[2]^ The amino acid sequences of the two proteins were input and separated by a colon to predict docking models within 48 cycles. The prediction results were visualized using PyMOL 2.4.0.

**1.16. Protein-protein interaction assays**

For co-immunoprecipitation (Co-IP) assay, the cells were lysed with NP-40 lysis buffer (Beyotime) containing 1 mM PMSF for 30 min at 4°C. The total protein concentrations were measured using BCA protein assay kit (Solarbio) according to the instructions. The lysates were divided into several tubes and precleared by adding protein A/G-agarose (Santa Cruz) for 30 min at 4°C. The samples were centrifuged to remove the precipitation and incubated with Notch1 antibody or normal rabbit IgG **(Table S6, Supporting Information)** for 2 hours, and then incubated with protein A/G-agarose overnight. The complexes were washed with NP-40 lysis buffer four times before being eluted in SDS-gel loading buffer and detected by western blotting.

For GST-pull down assay, the purified GST fusion proteins were incubated with precleared GST resin (Solarbio) for 5 hours at 4°C. The precipitation was washed four times with PBS and captured the Notch1 protein from prepared cell lysates at 4°C overnight. The complexes were washed four times with PBS before being eluted in SDS-gel loading buffer and detected by western blotting.

For bio‐layer interferometry (BLI) analyses performed by Octet Red96 system (Sartorius), purified NICD protein was immobilized on SA sensor (Sartorius) after labeling with Sulfo-NHS-Biotin (EZ-Link) and dialysis. To determine binding kinetics, five concentrations of purified ANXA1 protein were prepared with PBST (PBS containing 0.02% Tween-20) and measured from low to high concentrations by NICD-immobilized biosensors to obtain the association and dissociation sensorgram data. For detection of the interaction between protein and small molecular peptides, purified ANXA1 protein was immobilized on SSA sensor (Sartorius) to detect the binding kinetics of NICD peptides, which were diluted in SD buffer (PBST plus 0.1% w v^-1^ BSA). The kinetic parameters K_on_, K_off_ and K_D_ were determined using Octct data analysis software.

**1.17. Chemical cross-linking coupled with mass spectrometry assay**

For chemical cross-linking coupled with mass spectrometry assay, purified GST-ANXA1 and His Tag-NICD were cross-linked *in vitro* at room temperature for 1 hour with 1 mM BS3 (Thermo Fisher Scientific) in a 1:5 protein/cross-linker molar ratio, followed by incubating with 400 mM ammonium bicarbonate for 20 min to terminate the reaction. Proteins were precipitated with 6 times the volume of 100% acetone for 2 hours at −20°C. Protein pellets were collected using a centrifuge, supernatants were removed and air-dried. Proteins pellet was resuspended in 50 mM ammonium bicarbonate and samples were used for digestion using trypsin (1:50) digestion overnight at 37°C. Next day, peptides were reduced with 5 mM tris-carboxyethylphosphine (TCEP) and alkylated with 10 mM iodoacetamide (IAA) in the dark for 30 min at room temperature. Then, the reaction was stopped by acidification with formic acid (FA) to a final concentration of 5% (v:v). Peptides were desalted using Empore C18 (3M) following the manufacturer's instructions and dried.^[3]^

Peptides were redissolved in 0.1% FA and separated on C18 (75 × 15 cm, 1.9 µm C18 and 1 µm tip) with Easy-nLC 1200 system (Waters). Using the following mobile phases: 2% ACN incorporating 0.1% FA (Solvent A) and 80% ACN incorporating 0.1% FA (Solvent B). Samples were analyzed with a 60 min gradient at a flow rate of 450 nL min^-1^ as follows: 6-7% solvent B for 1 min, 7-40% solvent B for 52 min, 40-90% solvent B for 4 min, 90-100% solvent B for 2 min, and 100% solvent B for 1 min. Peptides were ionized by electrospray ionization at +2.1 kV. Tandem mass spectrometry analysis was carried out on a Q‐Exactive HF-X mass spectrometer (Thermo Fisher Scientific) under the control of Xcalibur software in a data-dependent mode. The mass spectrometer operated in data-dependent acquisition mode, and survey scans were obtained in a mass range of 400 to 1400 m z^-1^ with lock mass activated. In each scan cycle, fragmentation spectra of the 40 most intense peptide precursors in the survey scan were acquired in the higher-energy collisional dissociation (HCD) mode. Full MS scan at resolution = 120,000, followed by MS/MS scans at resolution = 15,000, NCE = 27, with an isolation width of 1.6 m z^-1^. The AGC targets for MS1 and MS2 scans were 3 × 10^6^ and 5 × 10^4^, respectively, and the maximum injection time for MS1 and MS2 were 20 and 30 ms, respectively. Precursors of +1, +2, +8 or above, or unassigned charge states were rejected; exclusion of isotopes was disabled; dynamic exclusion was set to 20 s.

Cross-linked peptides were identified using pLink2 software.^[4]^ NICD and ANXA1 sequences were downloaded from Uniprot. The minimal required peptide length was set to 6 amino acids and both protein and peptide identifications were accepted at a false discovery rate of 1%. 20 ppm for both precursor mass tolerance and fragment mass tolerance. Carbamidomethyl (C) was allowed as a variable modification. Trypsin was used, allowing 3 missed cleavages. All other parameters were left at default.

**1.18. Luciferase assay**

Cells were transfected with pGL4.19 plasmid containing the promoter sequence of *CDKN2B* to express firefly luciferase, and co-transfected with pRL-TK plasmid to express *Renilla* luciferase as a reference. The contents of the two luciferases were detected by a luminescence reader (SpectraMax L, Molecular Devices) according to the instructions of a duo-luciferase assay kit (GeneCopoeia).

**1.19. Statistical analysis**

All statistical data are presented as the means ± standard error of the mean (s.e.m.). The significance of the difference in the means was tested using GraphPad Prism version 8. Student’s t-test was used to compare the differences between the two groups. One-way ANOVA followed by Dunnett’s test was used to compare the differences between multiple groups with one control. Log-rank (Mantel-Cox) test was used to compare the differences between survival curves. Simple linear regression was used in co-expression analysis to reflect the correlation of protein expression. Fisher's exact test was used for the association between protein expression and sample source. *P* values of * < 0.05, ** < 0.01, and *** < 0.001 were considered significant.

**2. Supplementary Figures**


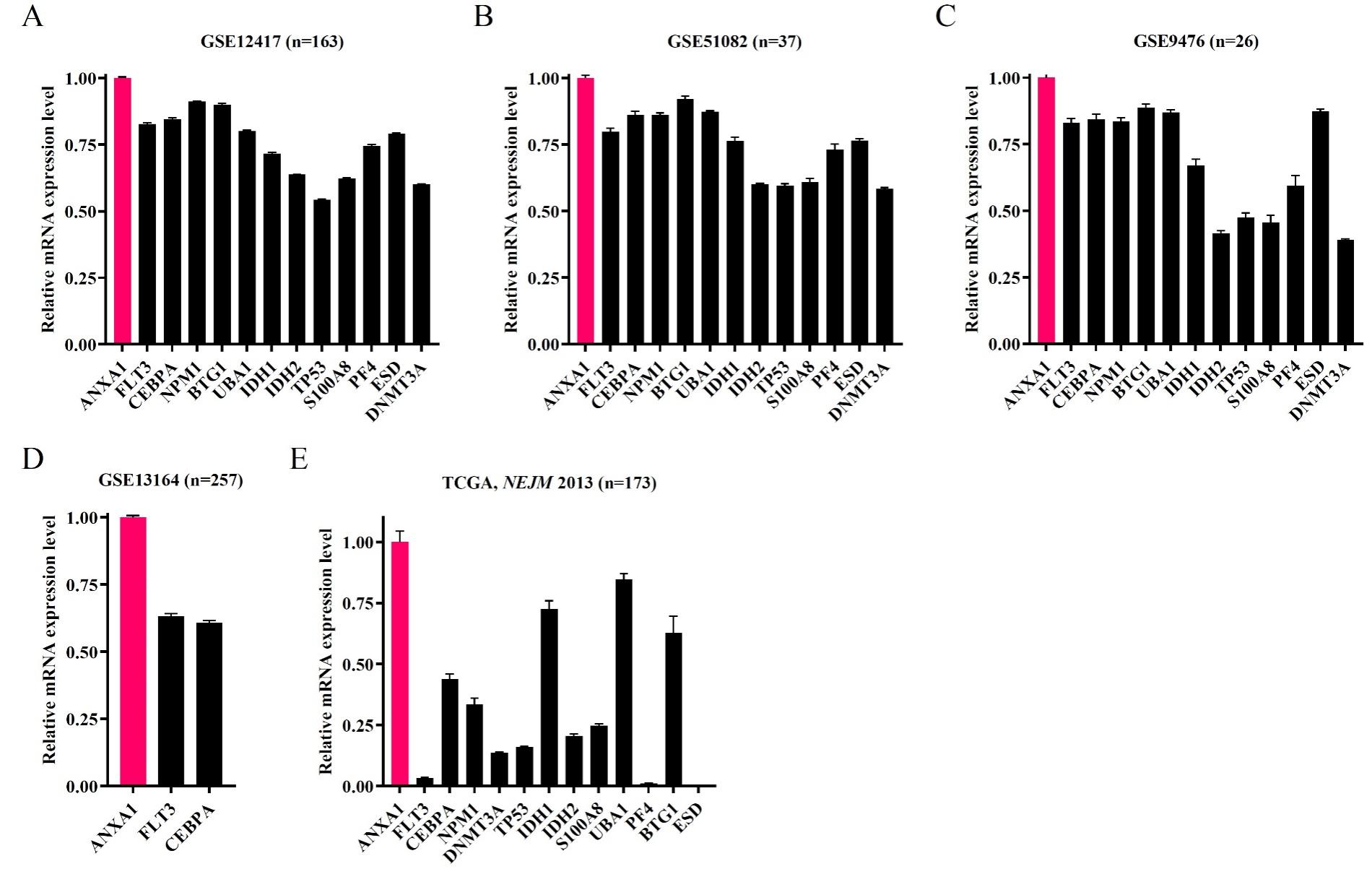


**Figure S1.** mRNA expression levels of AML biomarkers in AML patients, related to Figure 1A. Data are the means ± s.e.m..

**
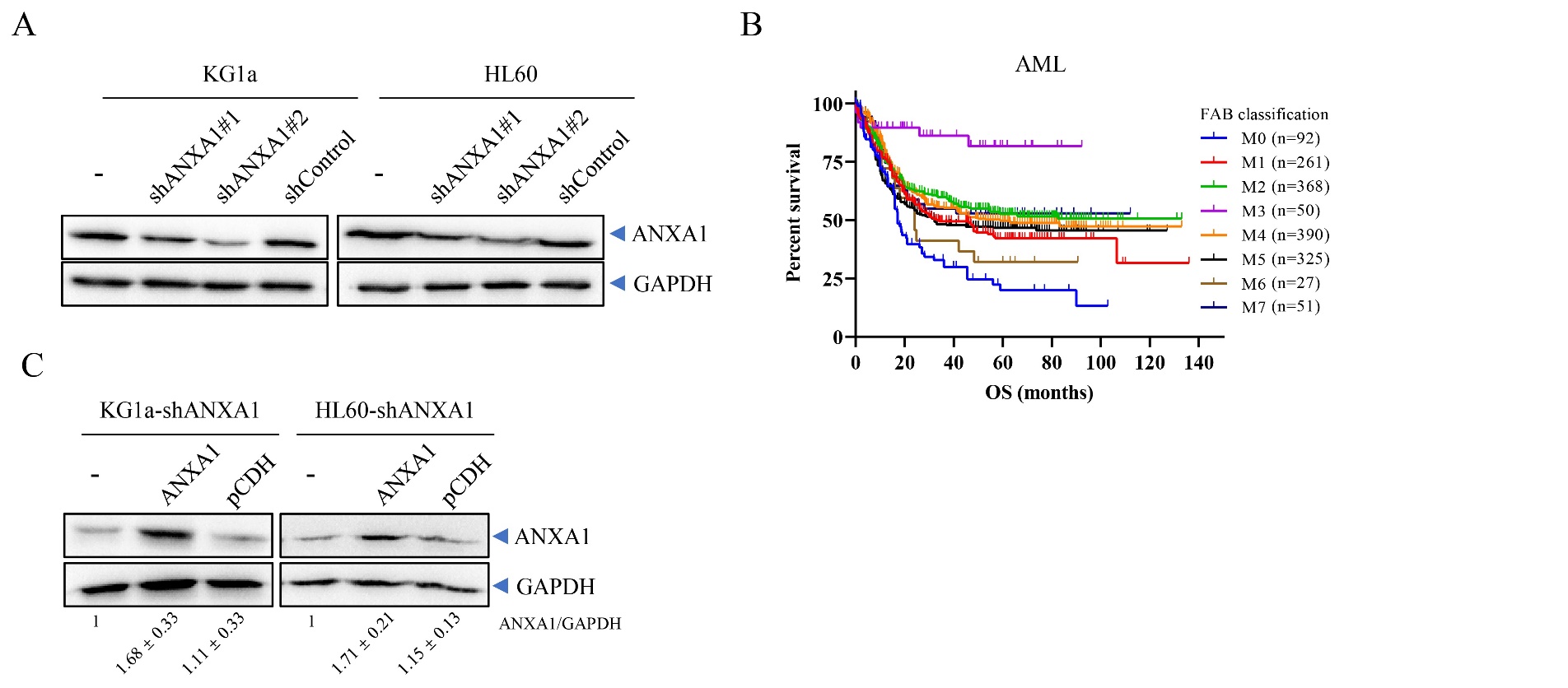
**

**Figure S2.** Verification of the construction effect of ANXA1 knockdown and overexpression cells, related to Figure 1. (A) Western blotting of ANXA1 expression in KG1a and HL60 cells transiently transfected with two shRNAs against ANXA1 or control shRNA. (B) Survival analysis of 1,564 AML patients with M0−M7 FAB classifications from cBioportal database. (C) Western blotting of ANXA1 expression in KG1a-shANXA1 and HL60-shANXA1 cells transiently transfected with ANXA1 overexpression or pCDH control plasmid.


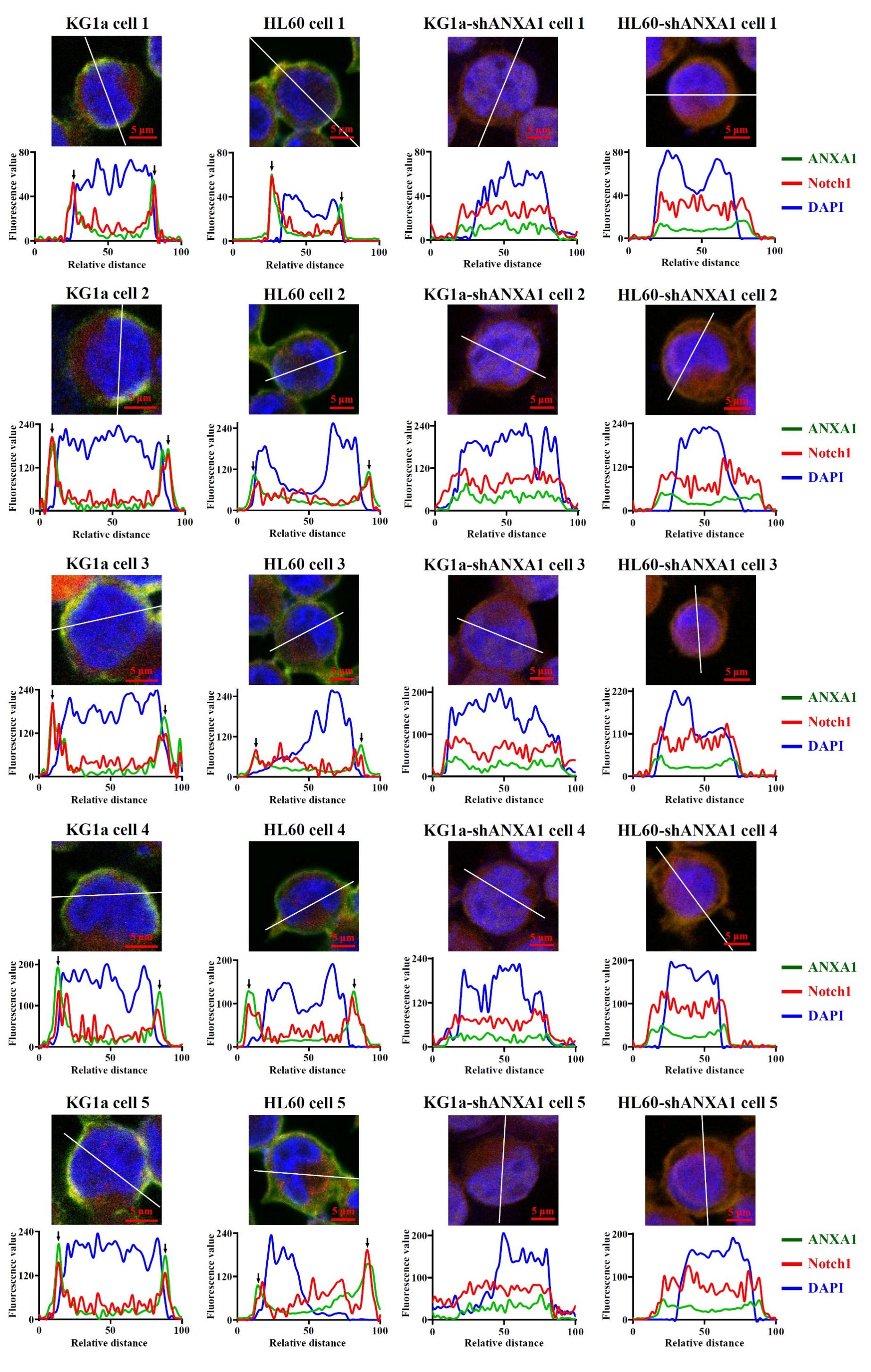


**Figure S3.** Profile analyses of the regions of interest in colocalization images, related to Figure 2C. White lines indicate the analysis regions. The arrows indicate the colocalization. The scale bar is 5 μm.

**
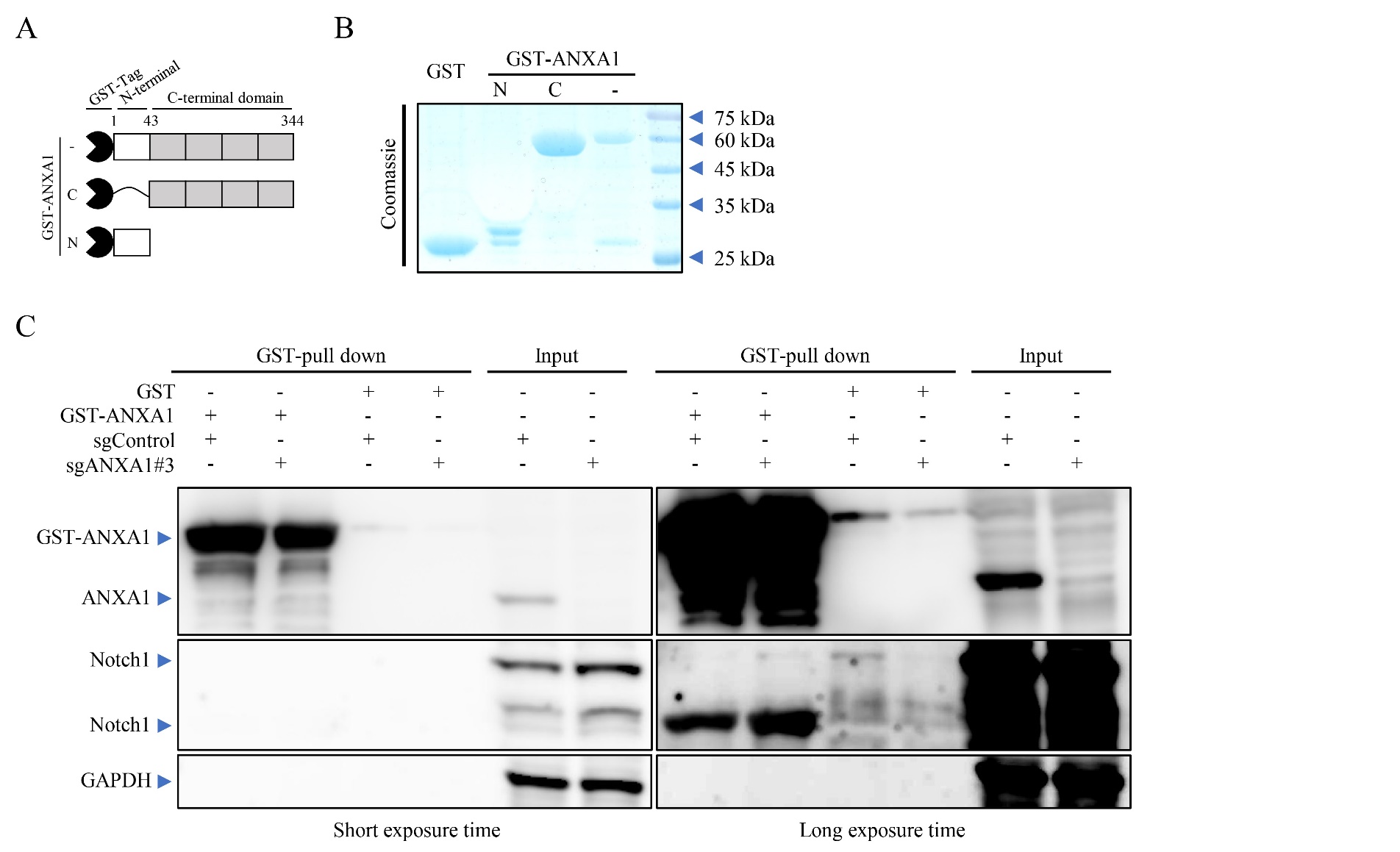
**

**Figure S4.** GST pull down analysis of the interaction between ANXA1 and Notch1, related to Figure 2. (A) Schematic diagram of GST-tagged fusion protein structure of ANXA1 full-length, C-terminal, and N-terminal domain. (B) Coomassie blue-stained SDS gel image of GST-tagged fusion protein of full-length ANXA1, and N-terminal, and C-terminal domains of ANXA1. (C) Western blotting of Notch1 and ANXA1 expression in complexes pulled-down by GST-ANXA1 in sgANXA1#3 and sgControl cells of KG1a.


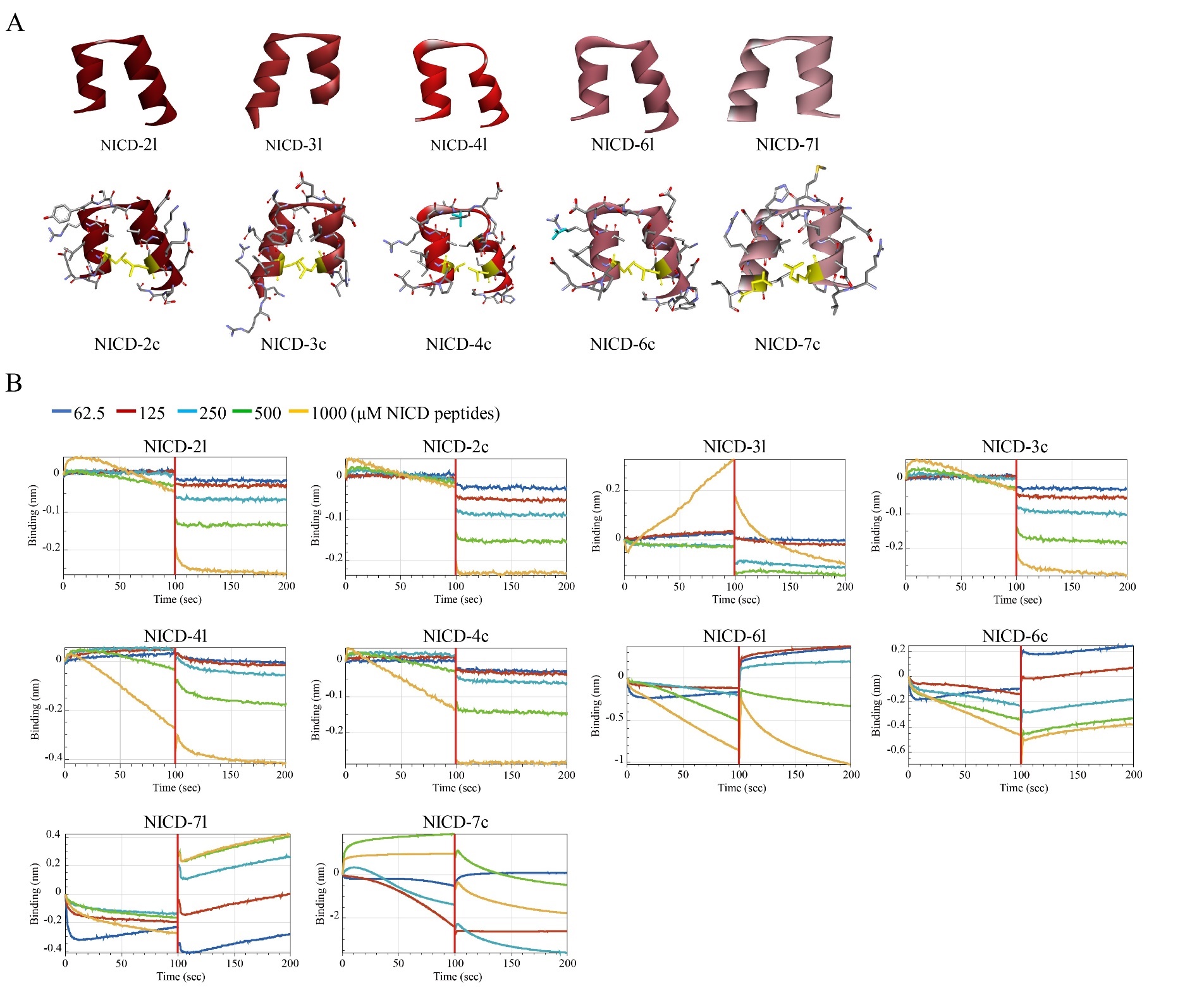


**Figure S5.** 3D structural models of the designed peptides mimicking the ankyrin domain of NICD and BLI analyses between ANXA1 and indicated peptides, related to Figure 2. “l” for linear peptides and “c” for cyclic peptides.

**
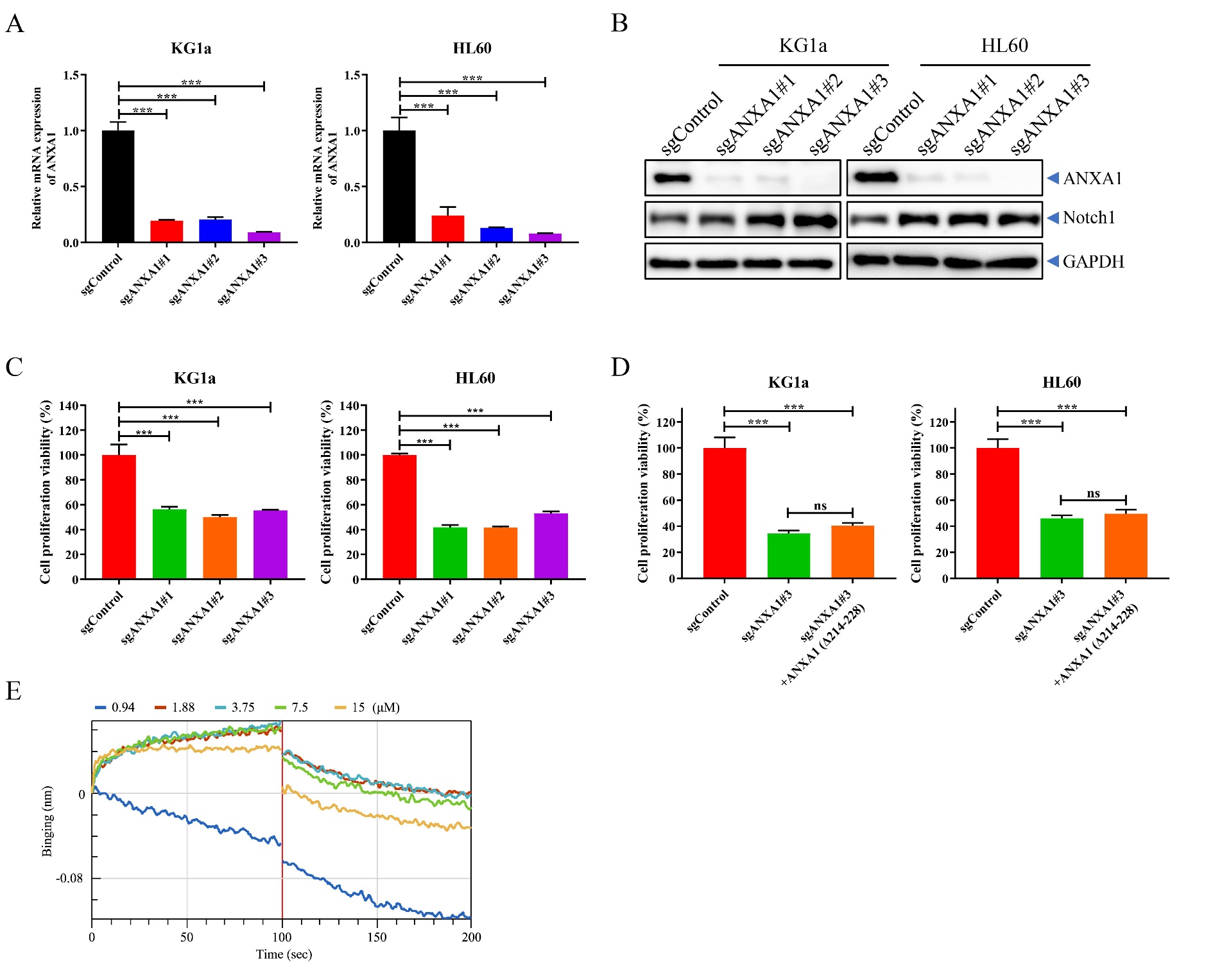
**

**Figure S6.** ANXA1 (214−228) and NICD (2,050−2,060) mediate the protein-protein interaction, related to Figure 2J. (A) mRNA expression of ANXA1 detected by qPCR in KG1a-sgANXA1 and HL60-sgANXA1 cells. (B) Western blotting of ANXA1 and NICD expression in KG1a-sgANXA1 and HL60-sgANXA1 cells. (C) The cell proliferation ability detected by CCK8 assay after 96 hours of culture of KG1a or HL60 cells stably transfected with sgANXA1 or sgControl plasmids. (D) The cell proliferation ability detected by CCK8 assay after 96 hours of culture of KG1a-sgANXA1 and HL60-sgANXA1 cells transiently transfected with ANXA1 (Δ214−228) plasmid. (E) BLI analyses of ANXA1 binding NICD (Δ2,050−2,060). Data in (A, C and D) are means ± s.e.m. of three independent experiments, one-way ANOVA with Dunnett’s test, compared with indicated group, ****P* < 0.001.

**
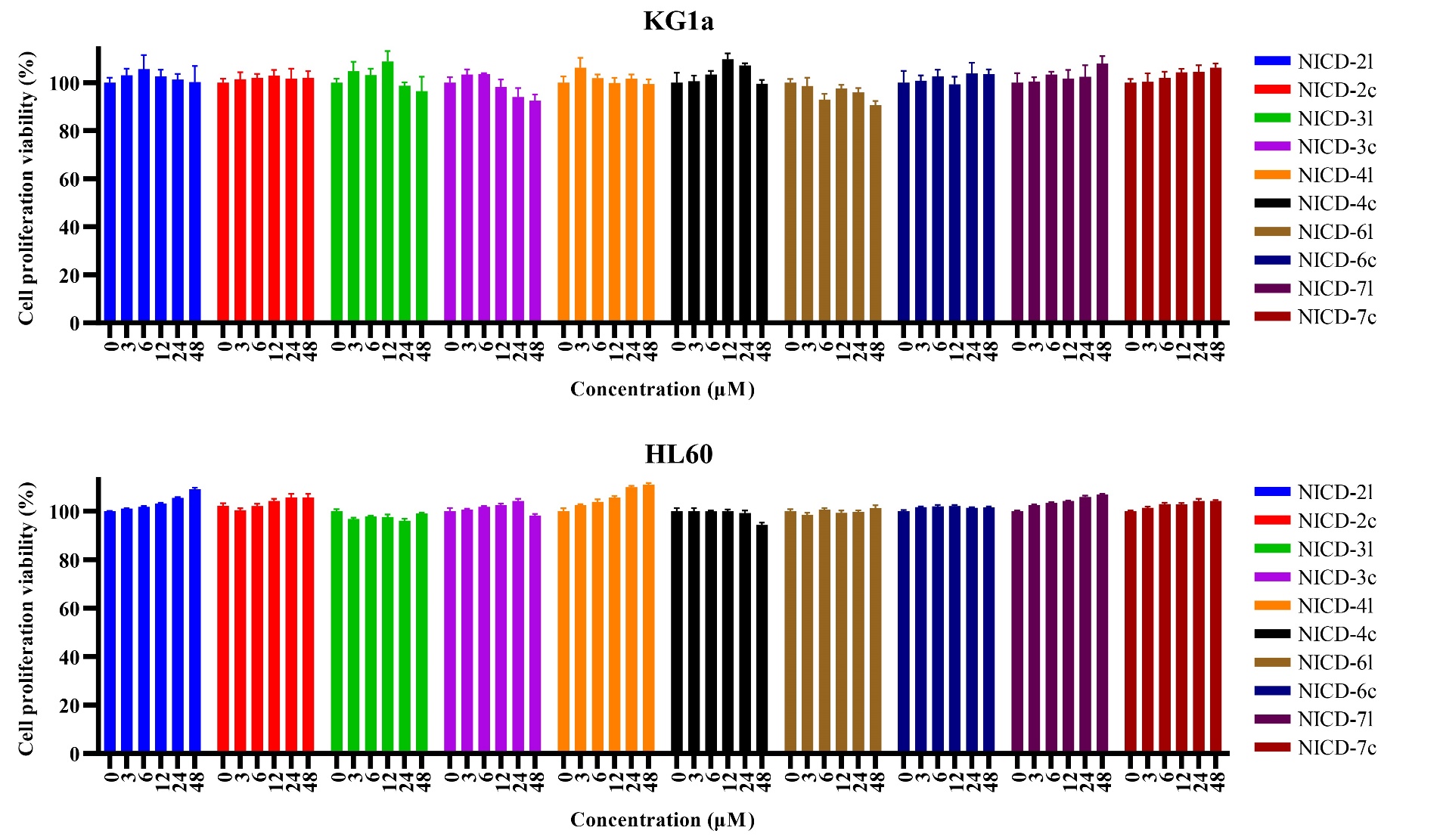
**

**Figure S7.** Effect of NICD peptides on AML cell proliferation, related to Figure 2K. The cell proliferation viability was detected by CCK8 assay after treatment with an indicated concentration of peptides of NICD for 48 hours. Data are means ± s.e.m. of three independent experiments.


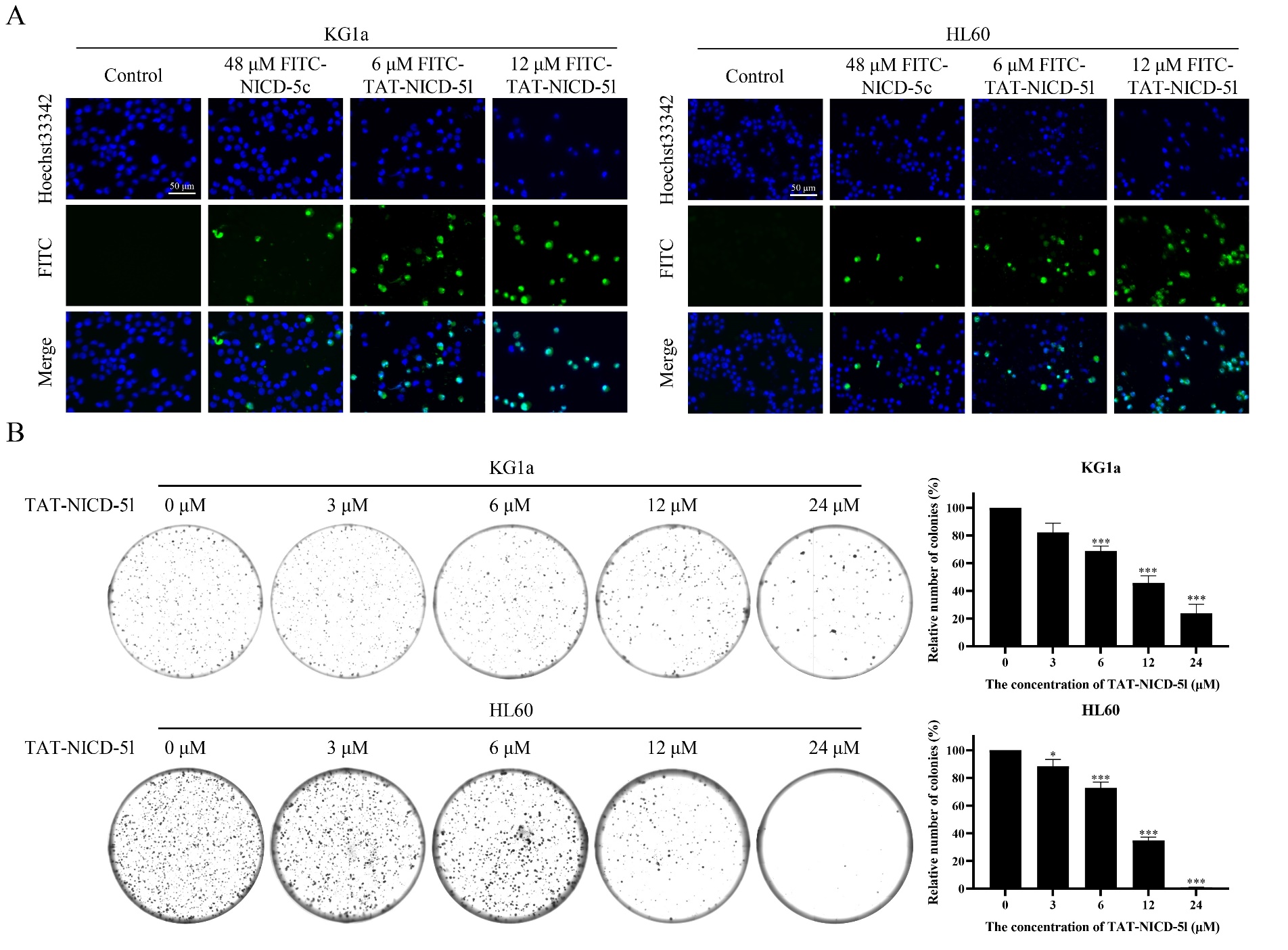


**Figure S8.** Effects of TAT-NICD-5l peptide on cell penetration and colony forming ability, related to Figure 2. (A) Representative fluorescence images of FITC-NICD-5c peptide and FITC-TAT-NICD-5l peptide permeated into KG1a and HL60 cells. Cells were continuously treated with peptides for 24 hours in the incubator. The scale bar is 50 μm. (B) Colony forming ability of KG1a and HL60 cells treated with TAT-NICD-5l peptide. Data are means ± s.e.m. of six independent experiments, one-way ANOVA with Dunnett’s test, compared with 0 μM group, **P* < 0.05, ****P* < 0.001.

**
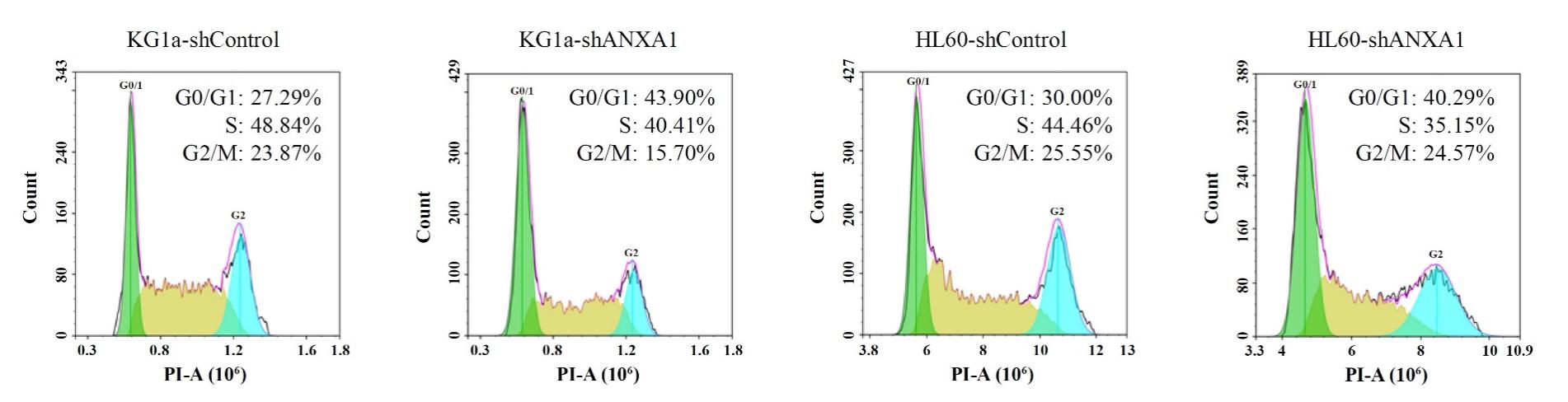
**

**Figure S9**. Representative cycle distribution images of KG1a and HL60 cells stably transfected with shANXA1 or shControl plasmids, related to Figure 3F.

**
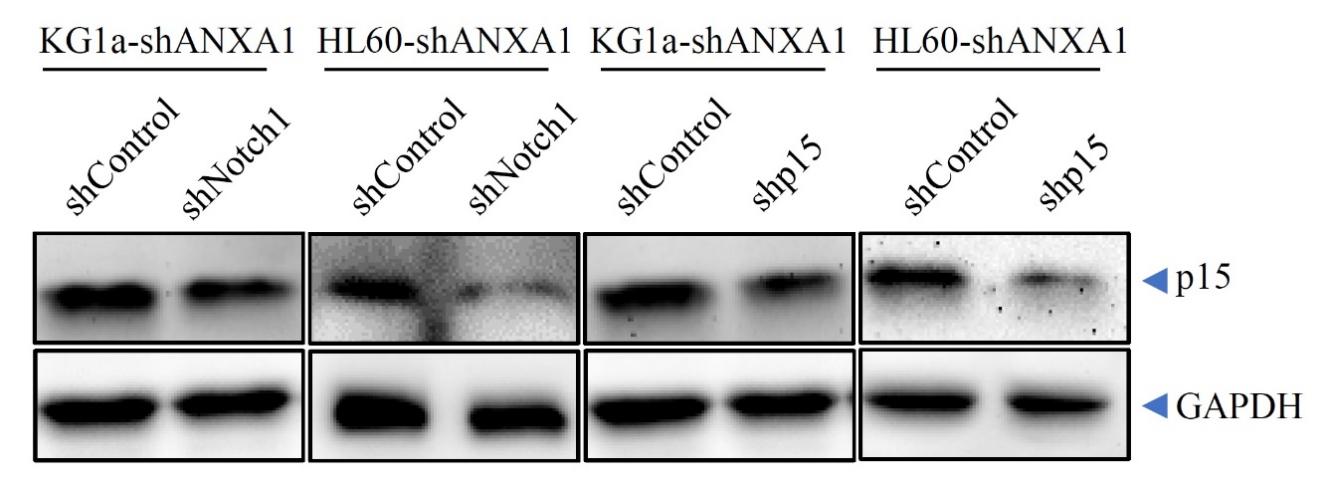
**

**Figure S10**. Verification of the effect of Notch1 and p15 knockdown on the expression of p15 in KG1a-shANXA1 and HL60-shANXA1 cells, related to Figure 4C. Western blotting of p15 expression in KG1a-shANXA1 and HL60-shANXA1 cells transfected with shNotch1, shp15 or control shRNA.

**
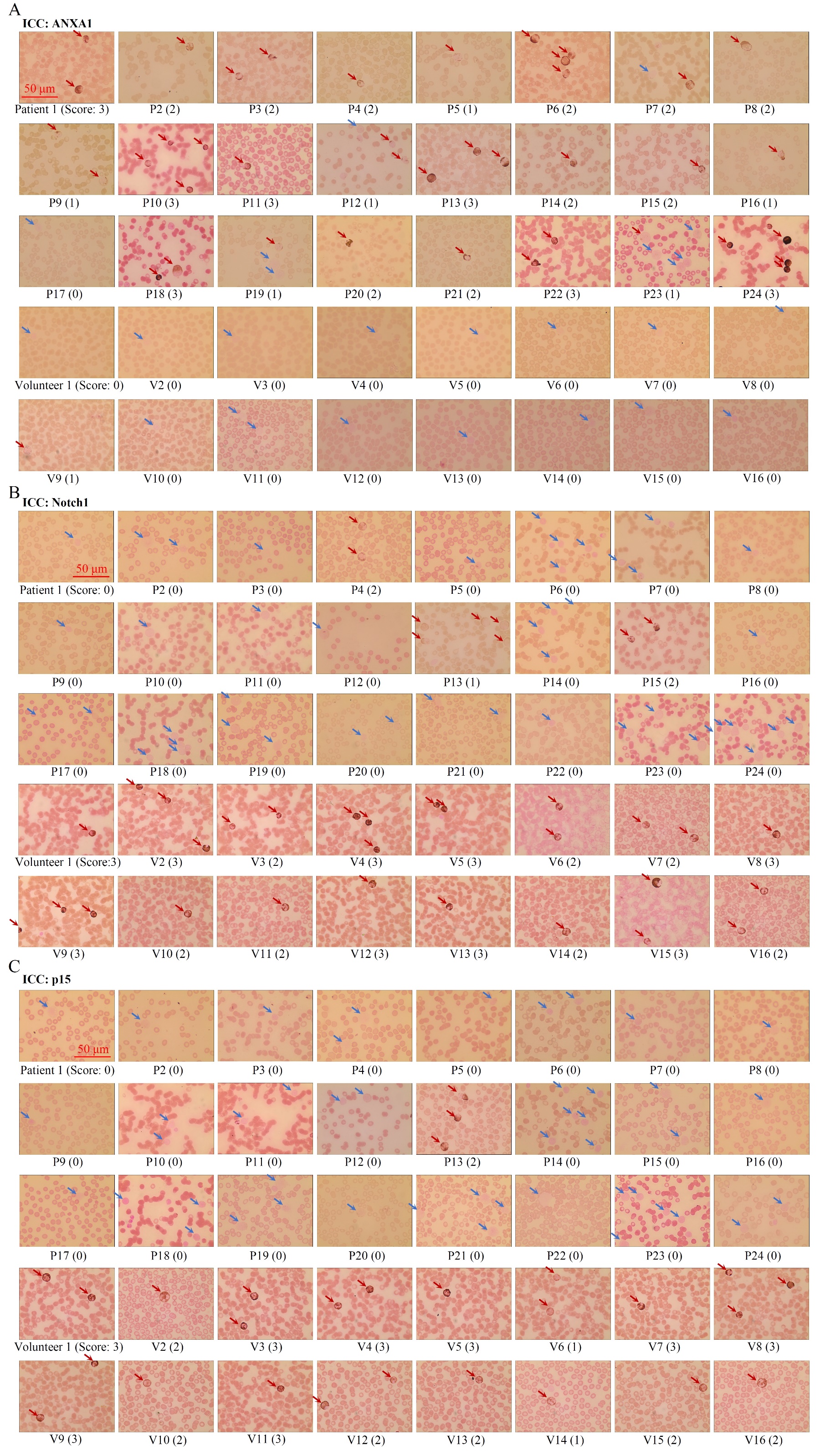
**

**Figure S11.** The expression level of ANXA1, Notch1 and p15 in peripheral blood leukocytes of AML patients, related to Figure 5. (A) Immunocytochemical images of ANXA1-stained peripheral blood from AML patients (P) and volunteers (V). (B) Immunocytochemical images of Notch1-stained peripheral blood from AML patients (P) and volunteers (V). (C) Immunocytochemical images of p15-stained peripheral blood from AML patients (P) and volunteers (V). The red and blue arrows indicate positive and negative stained white blood cells, respectively. The scale bar is 50 μm.


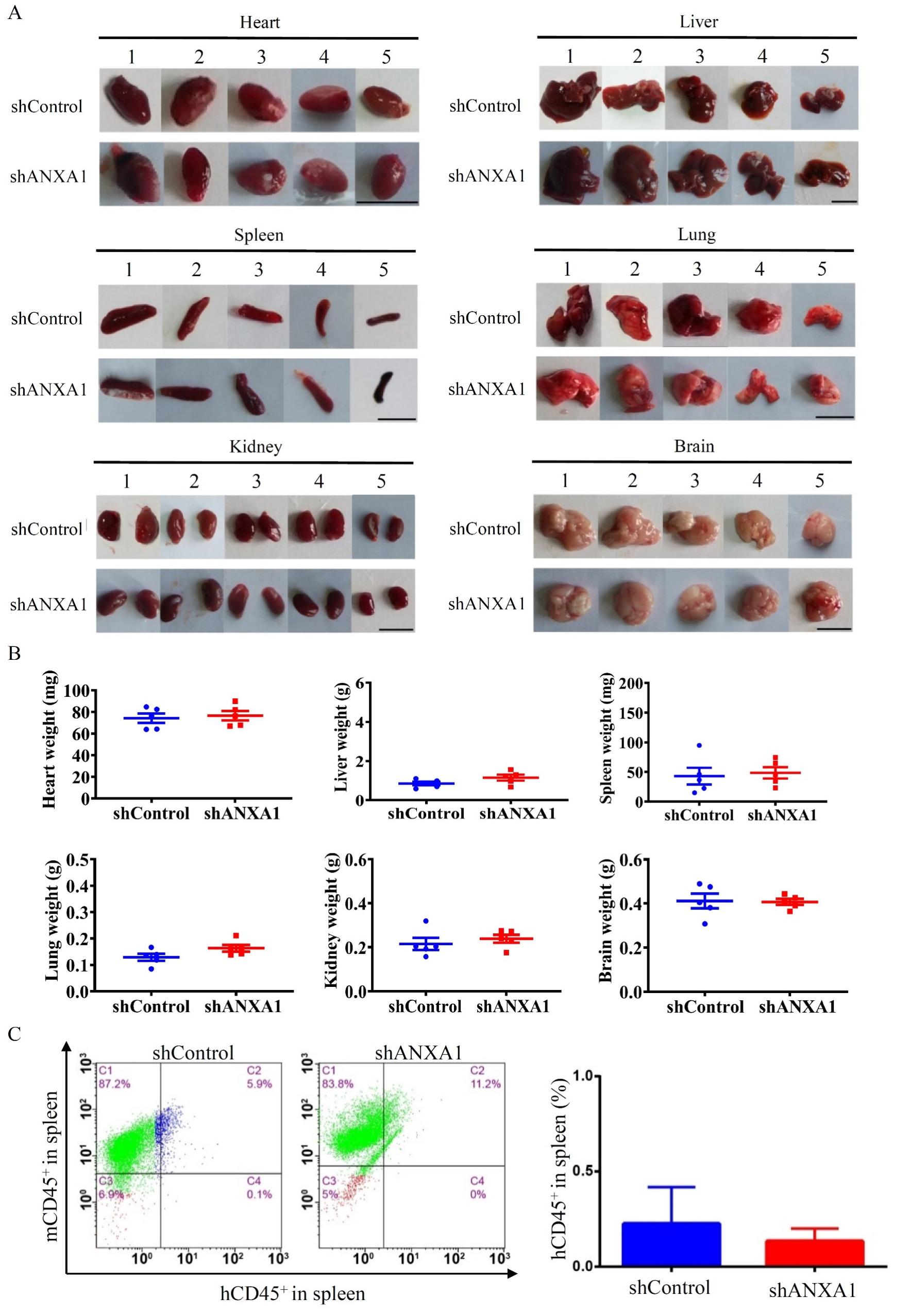


**Figure S12.** Effects of AML mouse models constructed by KG1a-shControl and KG1a-shANXA1 cells on viscera and brain, related to Figure 6. (A) The organs excised from the mice in KG1a-shControl and KG1a-shANXA1 groups. (B) The quantification analysis of weights of each organ in KG1a-shControl and KG1a-shANXA1 groups. Data are means ± s.e.m. (C) The hCD45^+^ cells in spleen were analyzed by flow cytometry in KG1a-shControl and KG1a-shANXA1 groups. Data are means ± s.e.m..

**
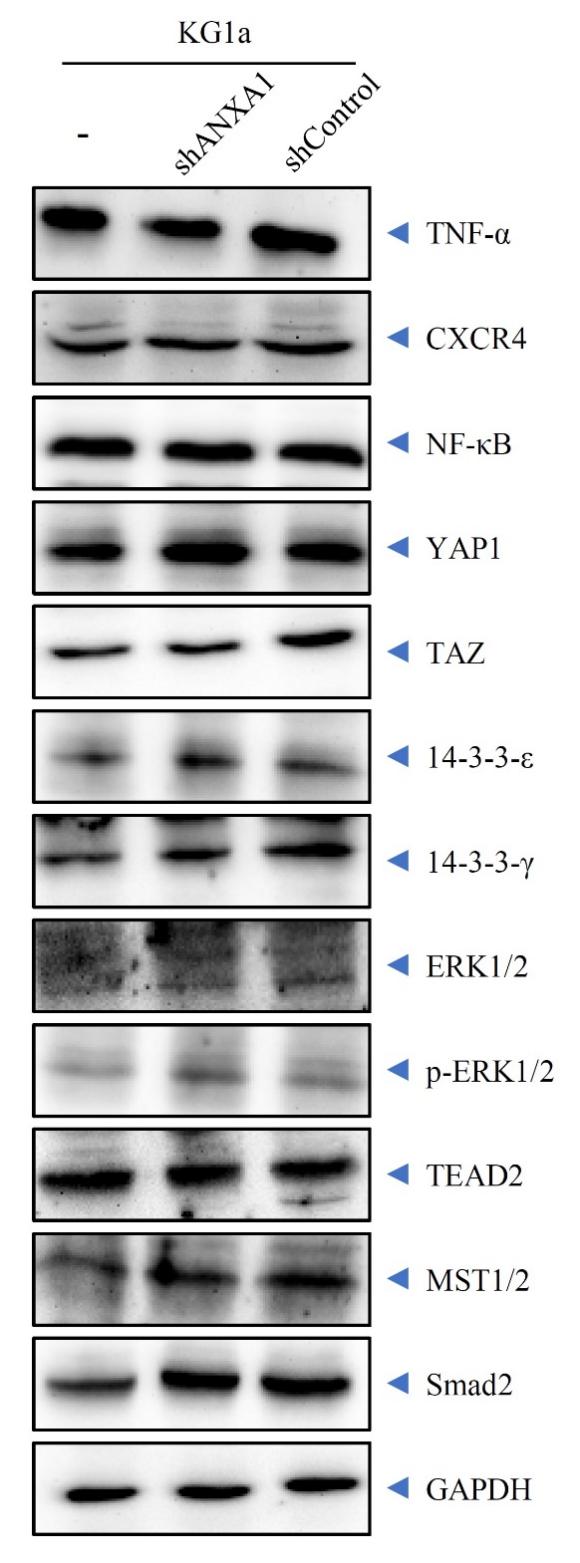
**

**Figure S13.** Protein expression levels in several classical signal pathways after knockdown of ANXA1 in KG1a cells, related to Figure 1.

**
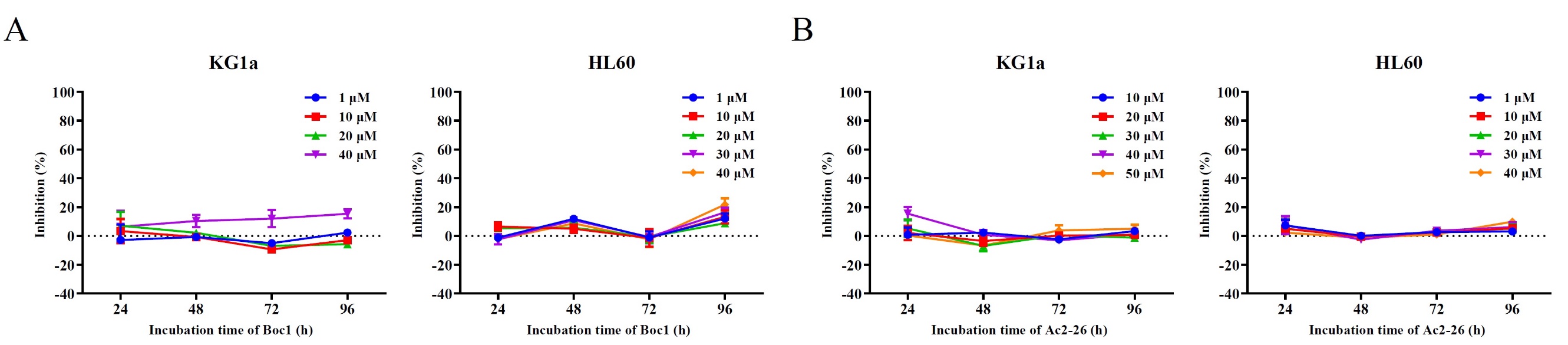
**

**
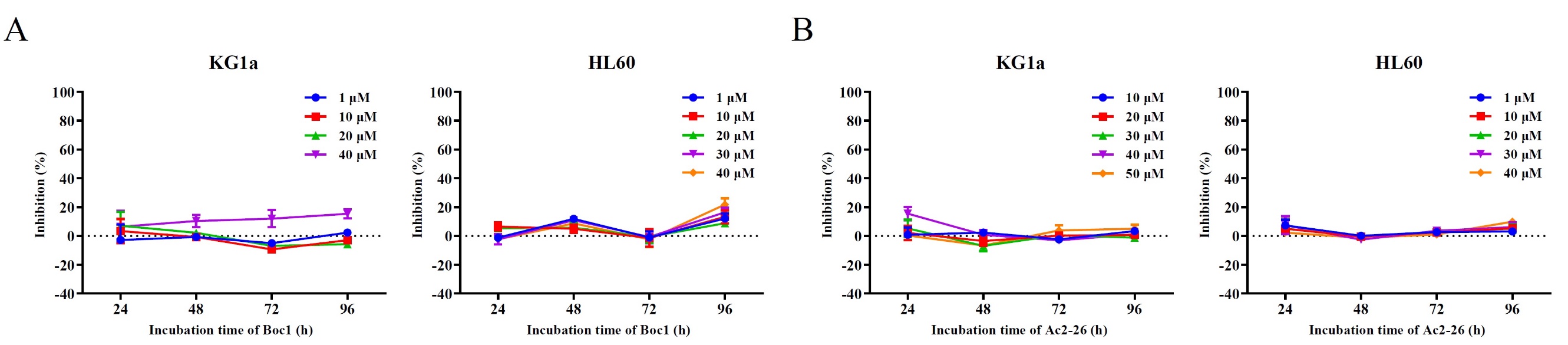
**

**Figure S14.** Effects of blocking or activation of formyl peptide receptor (FPR) on the proliferation of AML cells, related to Figure 1. (A and B) The inhibitory effect of FPR antagonist Boc-1 (A) or FPR agonist Ac2-26 (B) on the proliferation of KG1a and HL60 cells was detected by MTT assay at the indicated concentration and treatment time. Data are means ± s.e.m. of three independent experiments.


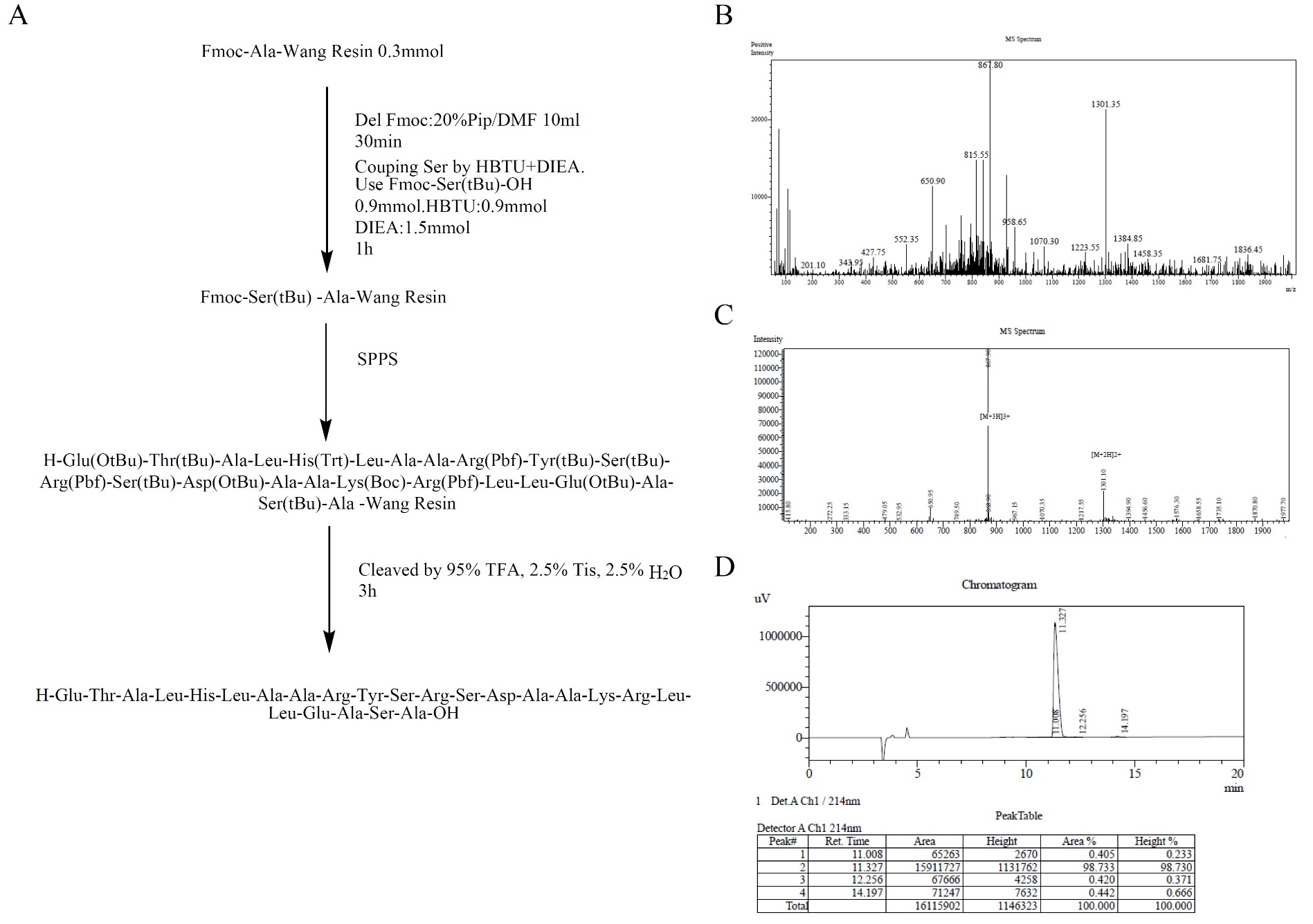


**Figure S15**. Synthesis process of NICD-2l peptide, related to Figure 2. (A) Flow chart of peptide synthesis. (B) Mass spectrometry results of crude peptide. (C) Mass spectrometry results of purified peptide. (D) The purity of the peptide was detected by high-performance liquid chromatography.


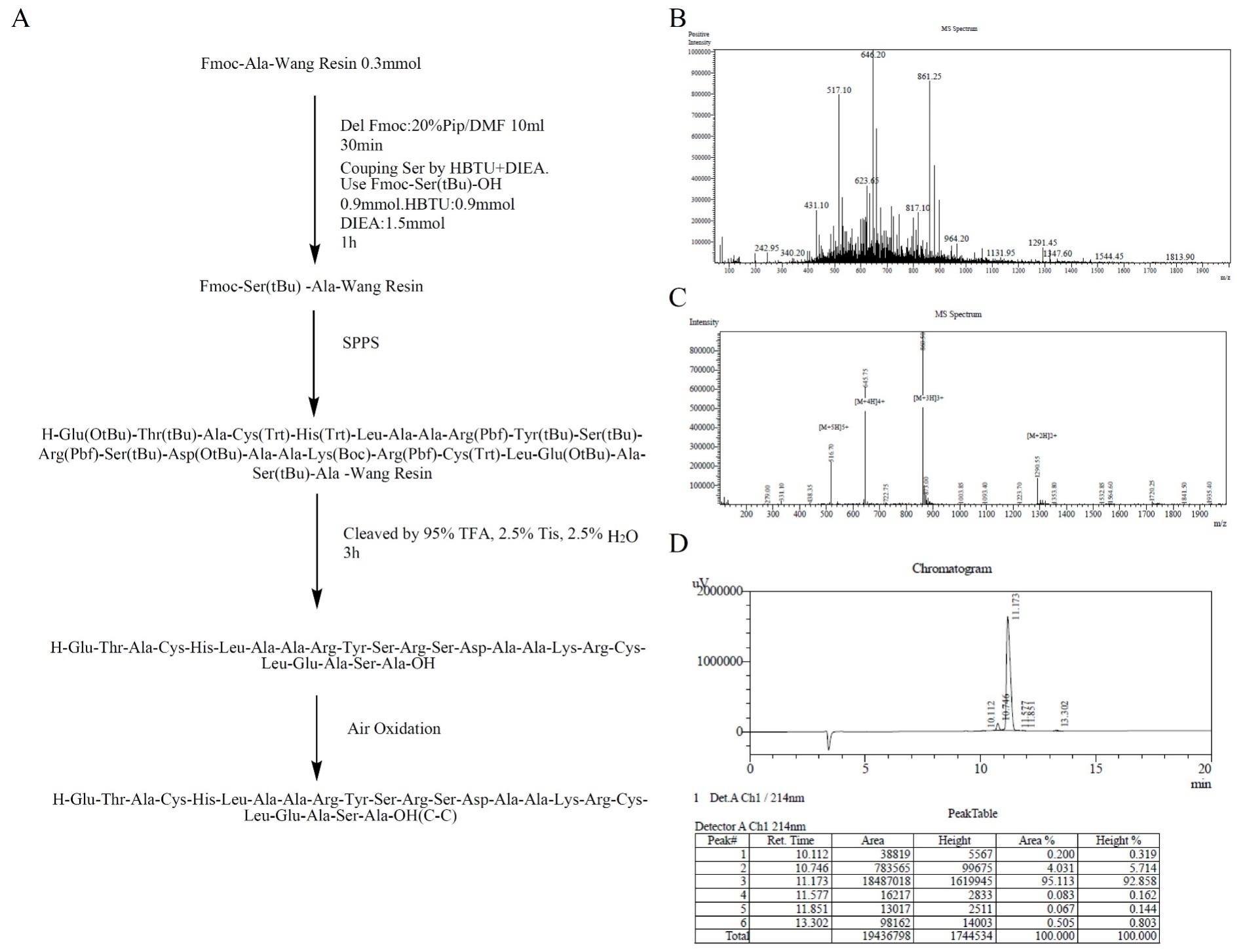


**Figure S16.** Synthesis process of NICD-2c peptide, related to Figure 2. (A) Flow chart of peptide synthesis. (B) Mass spectrometry results of crude peptide. (C) Mass spectrometry results of purified peptide. (D) The purity of the peptide was detected by high-performance liquid chromatography.


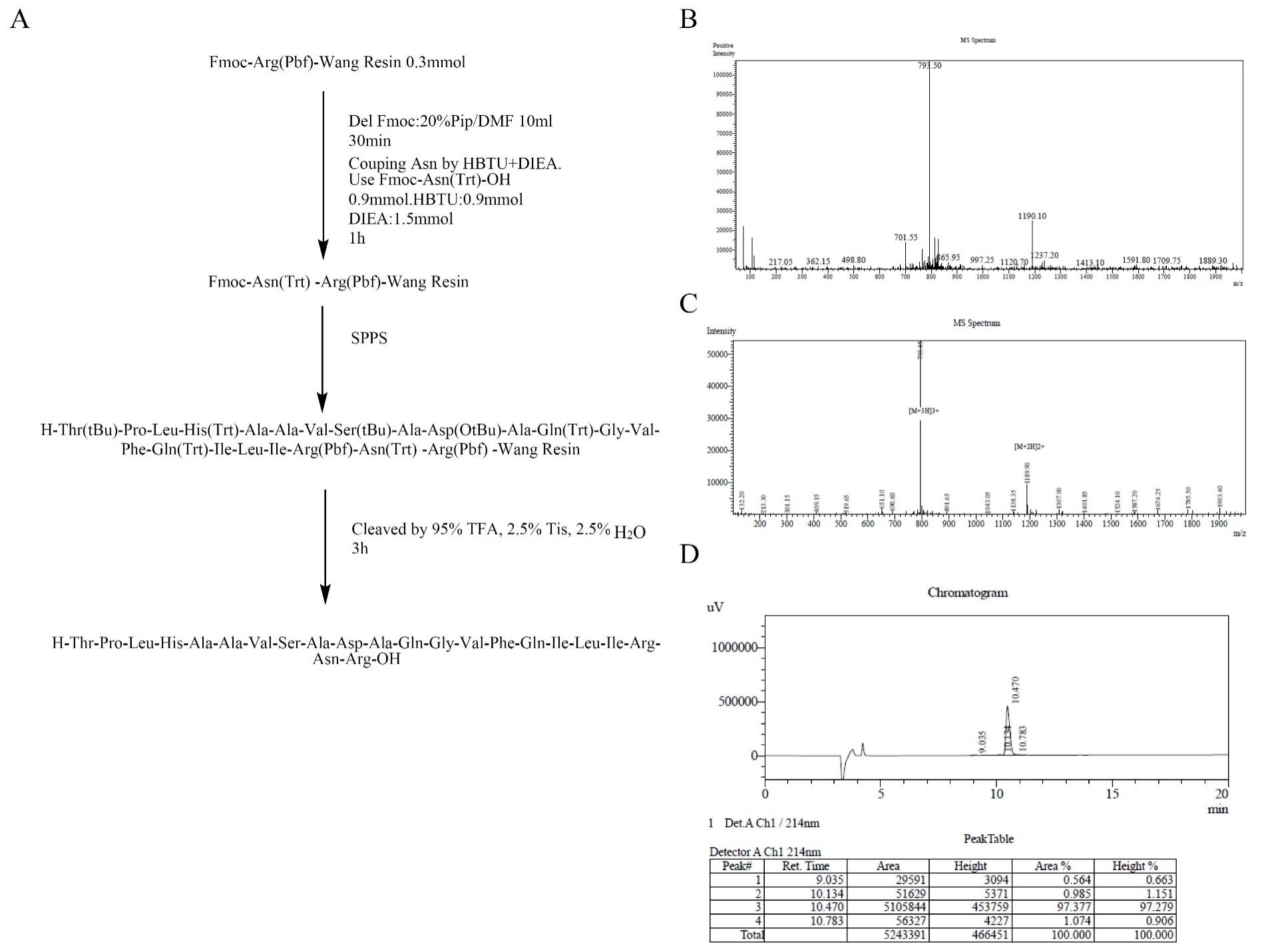


**Figure S17.** Synthesis process of NICD-3l peptide, related to Figure 2. (A) Flow chart of peptide synthesis. (B) Mass spectrometry results of crude peptide. (C) Mass spectrometry results of purified peptide. (D) The purity of the peptide was detected by high-performance liquid chromatography.


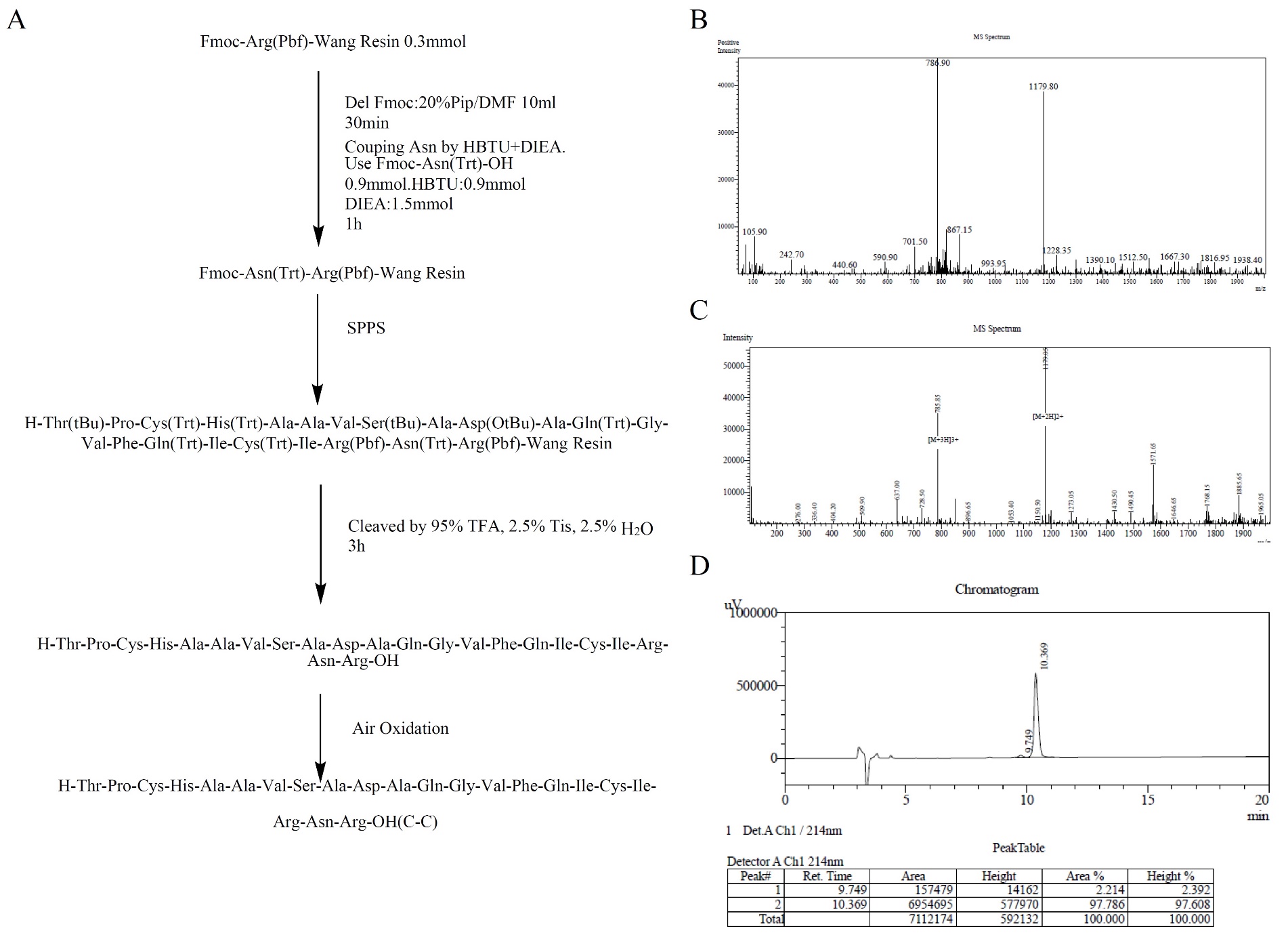


**Figure S18.** Synthesis process of NICD-3c peptide, related to Figure 2. (A) Flow chart of peptide synthesis. (B) Mass spectrometry results of crude peptide. (C) Mass spectrometry results of purified peptide. (D) The purity of the peptide was detected by high-performance liquid chromatography.


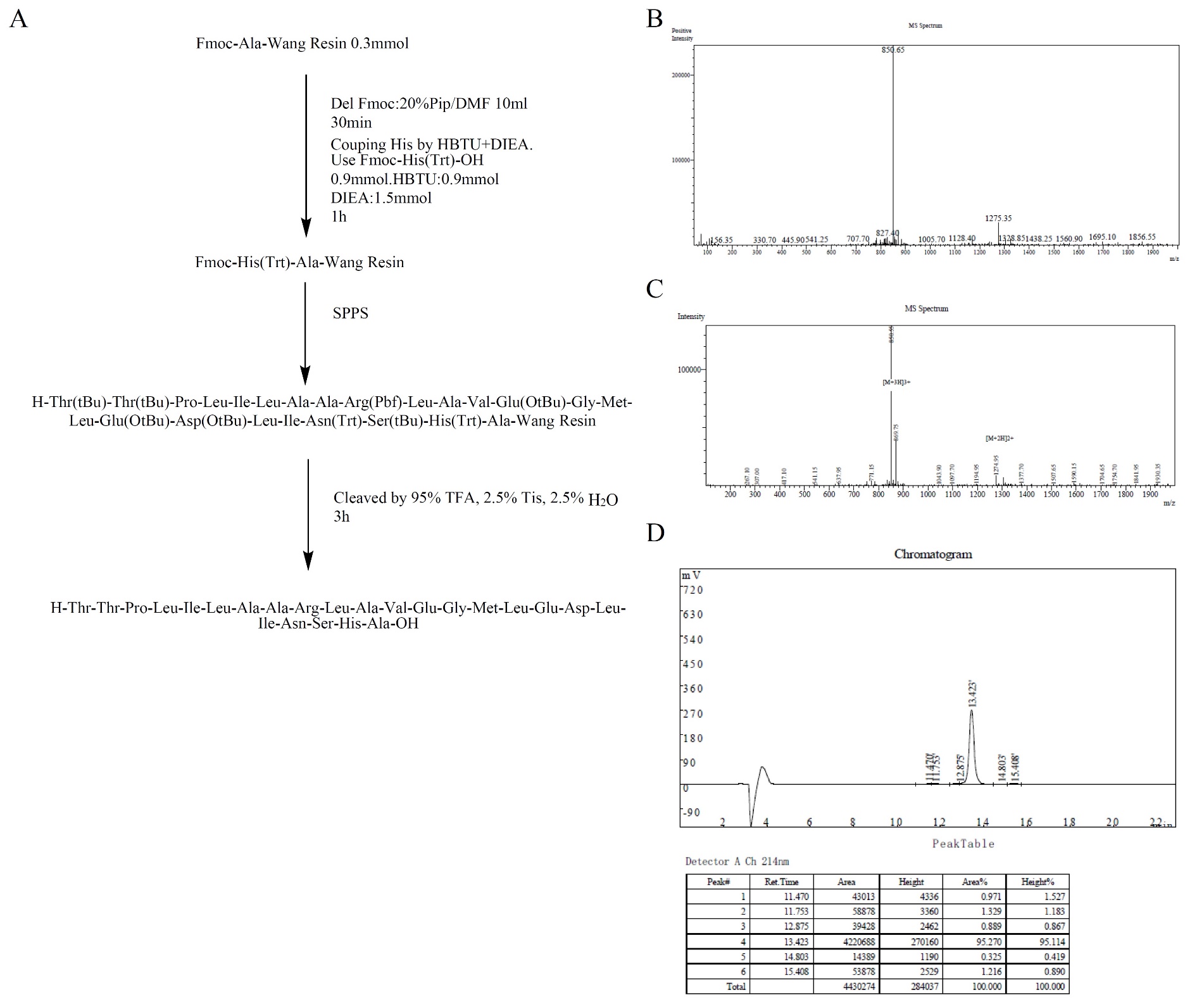


**Figure S19.** Synthesis process of NICD-4l peptide, related to Figure 2. (A) Flow chart of peptide synthesis. (B) Mass spectrometry results of crude peptide. (C) Mass spectrometry results of purified peptide. (D) The purity of the peptide was detected by high-performance liquid chromatography.


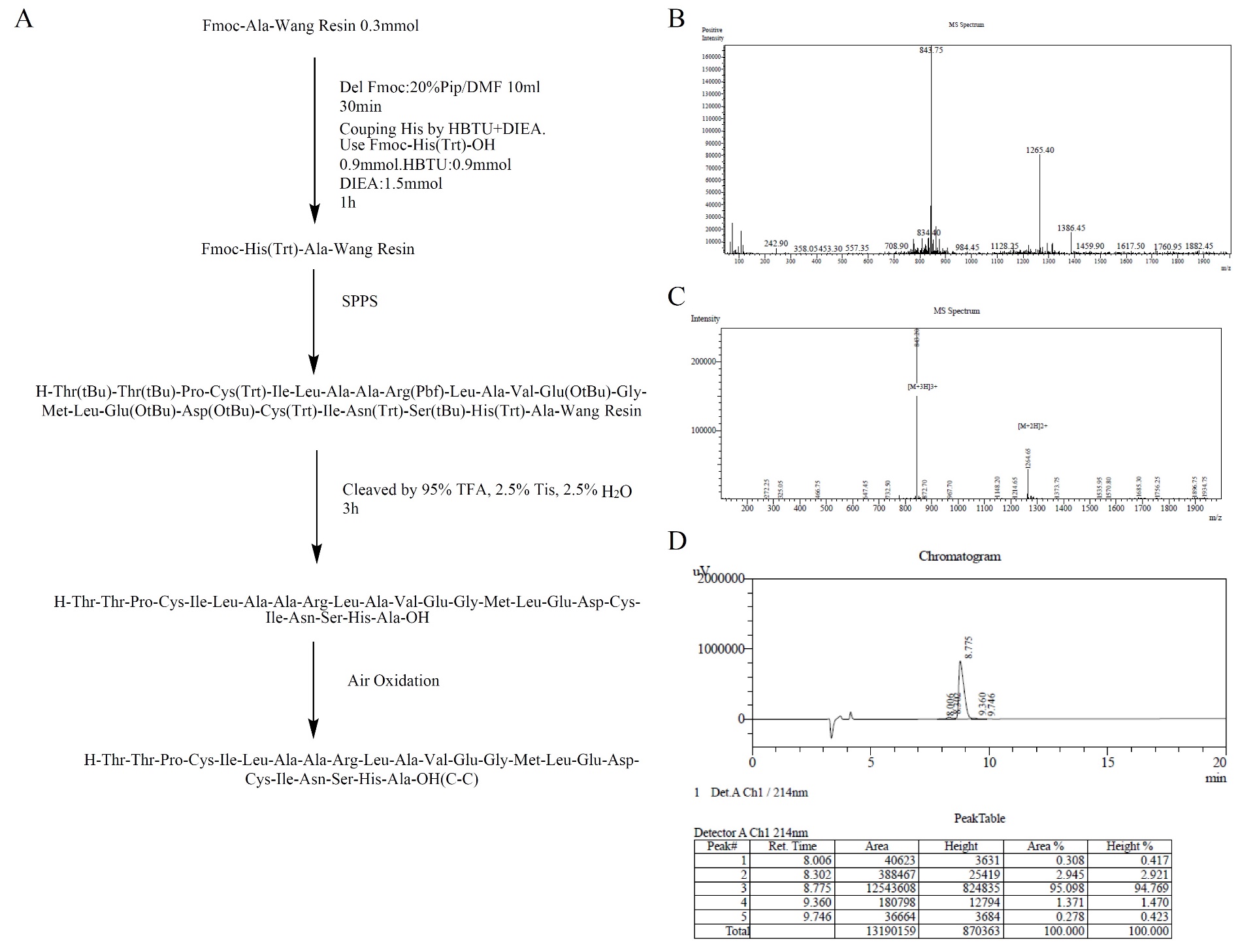


**Figure S20.** Synthesis process of NICD-4c peptide, related to Figure 2. (A) Flow chart of peptide synthesis. (B) Mass spectrometry results of crude peptide. (C) Mass spectrometry results of purified peptide. (D) The purity of the peptide was detected by high-performance liquid chromatography.


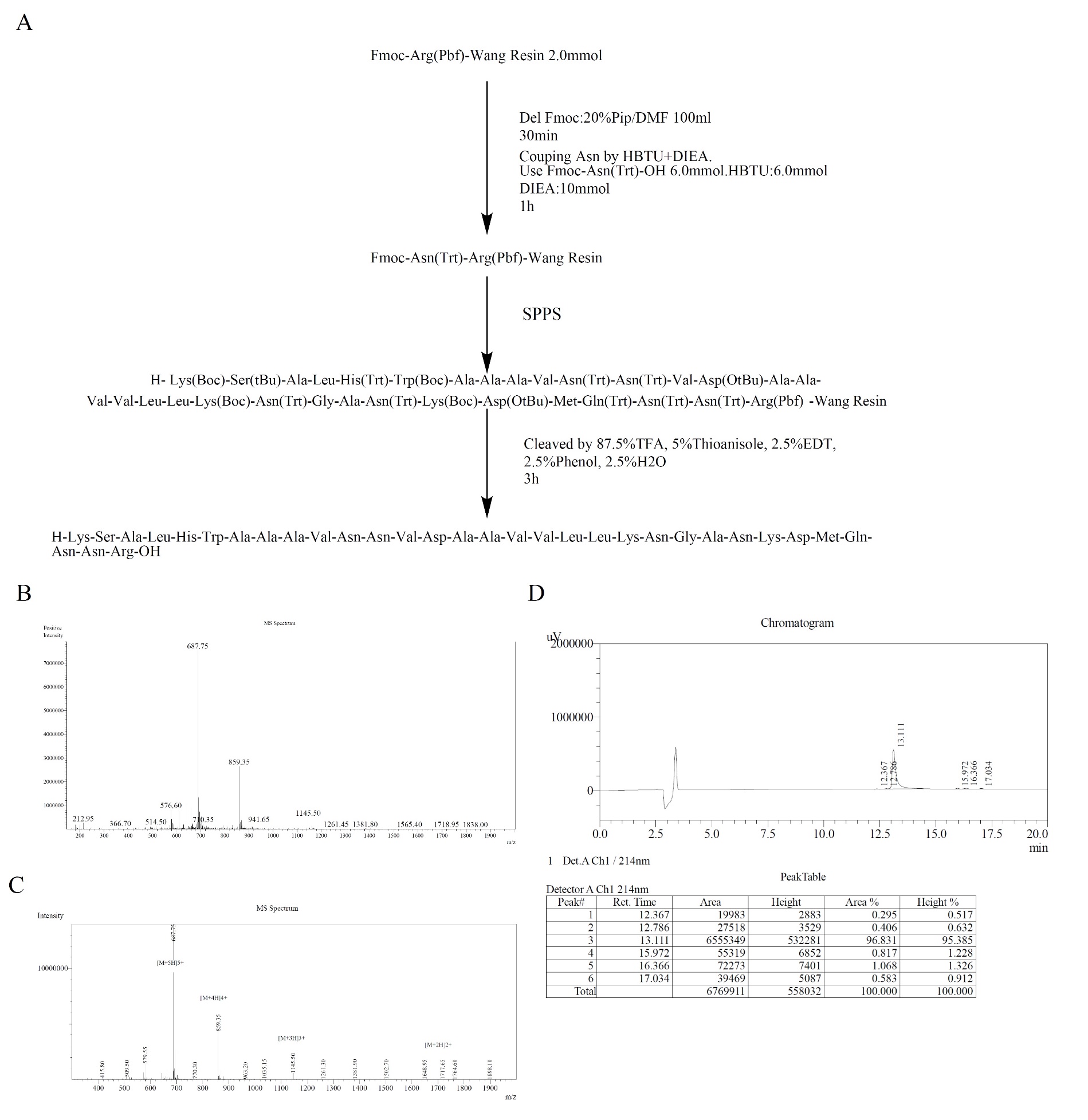


**Figure S21.** Synthesis process of NICD-5l peptide, related to Figure 2. (A) Flow chart of peptide synthesis. (B) Mass spectrometry results of crude peptide. (C) Mass spectrometry results of purified peptide. (D) The purity of the peptide was detected by high-performance liquid chromatography.


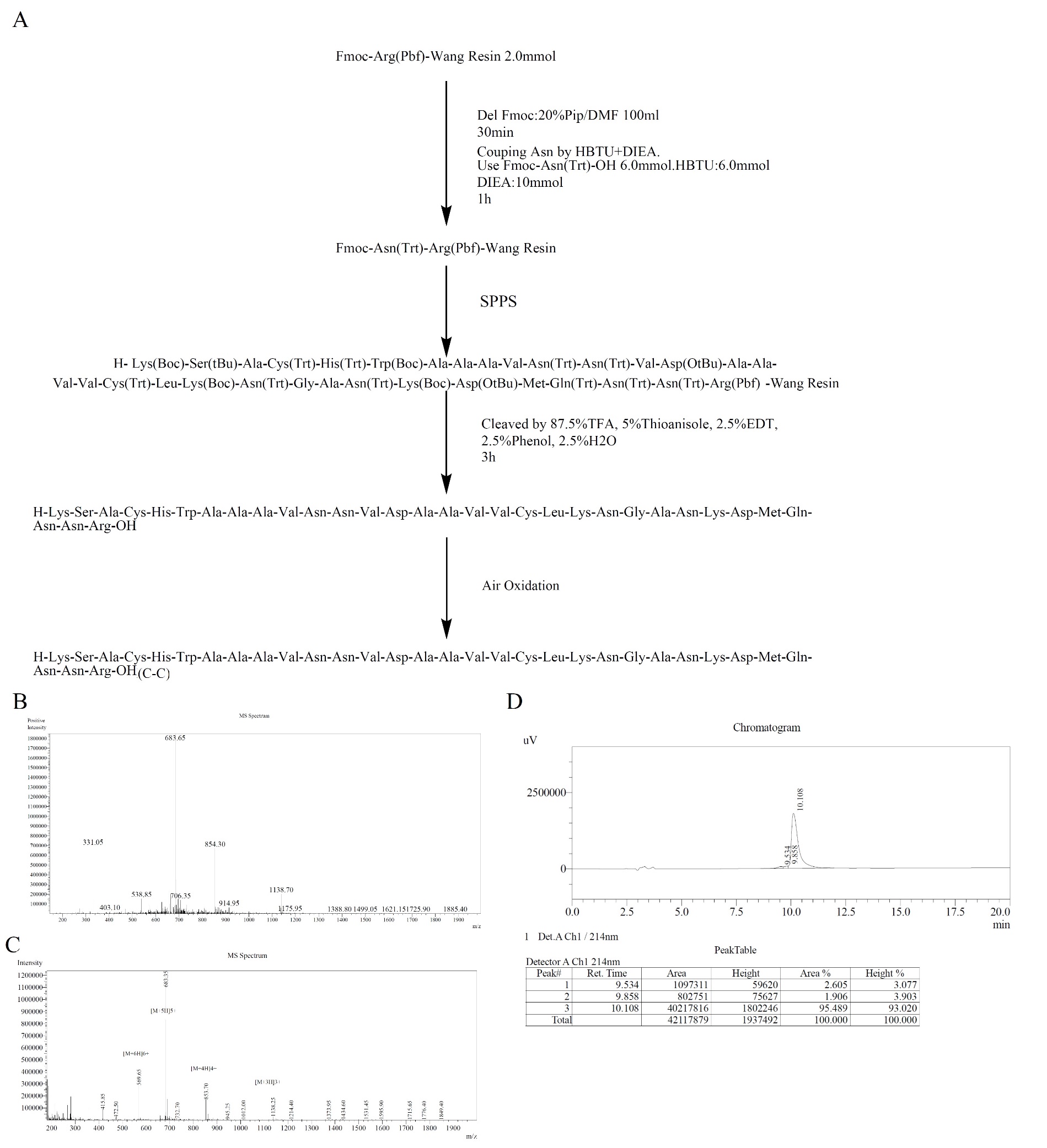


**Figure S22.** Synthesis process of NICD-5c peptide, related to Figure 2. (A) Flow chart of peptide synthesis. (B) Mass spectrometry results of crude peptide. (C) Mass spectrometry results of purified peptide. (D) The purity of the peptide was detected by high-performance liquid chromatography.


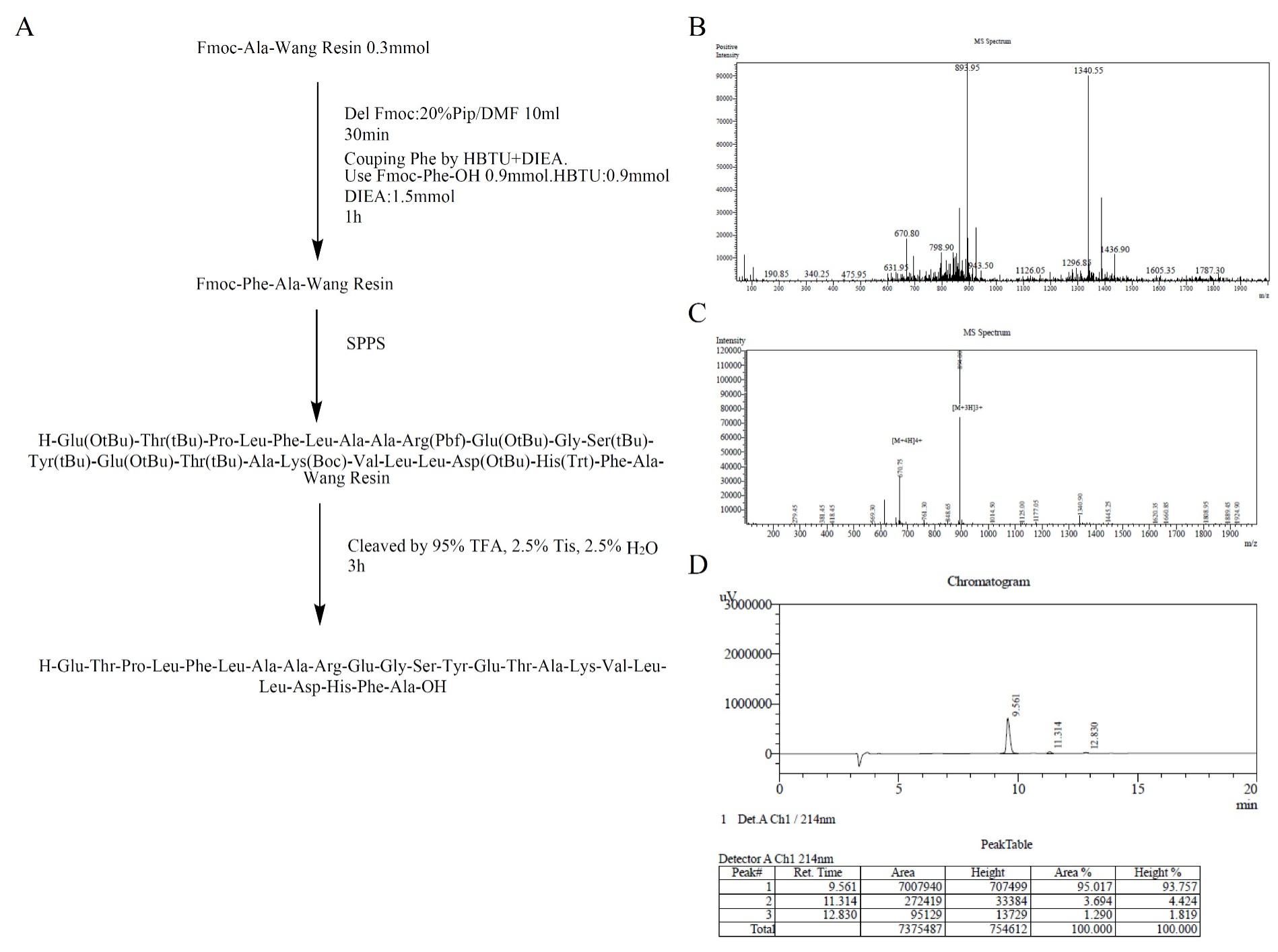


**Figure S23.** Synthesis process of NICD-6l peptide, related to Figure 2. (A) Flow chart of peptide synthesis. (B) Mass spectrometry results of crude peptide. (C) Mass spectrometry results of purified peptide. (D) The purity of the peptide was detected by high-performance liquid chromatography.


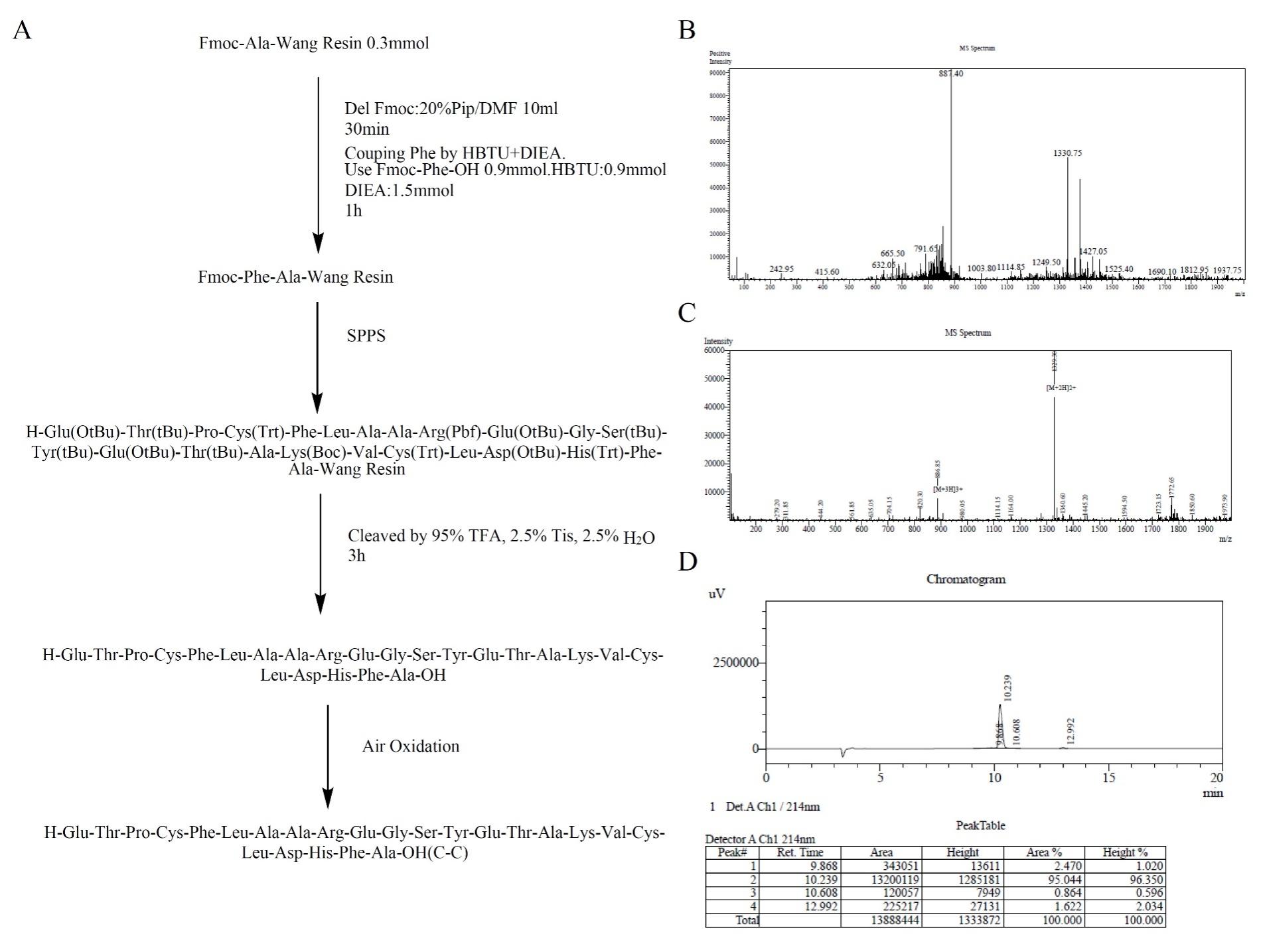


**Figure S24.** Synthesis process of NICD-6c peptide, related to Figure 2. (A) Flow chart of peptide synthesis. (B) Mass spectrometry results of crude peptide. (C) Mass spectrometry results of purified peptide. (D) The purity of the peptide was detected by high-performance liquid chromatography.


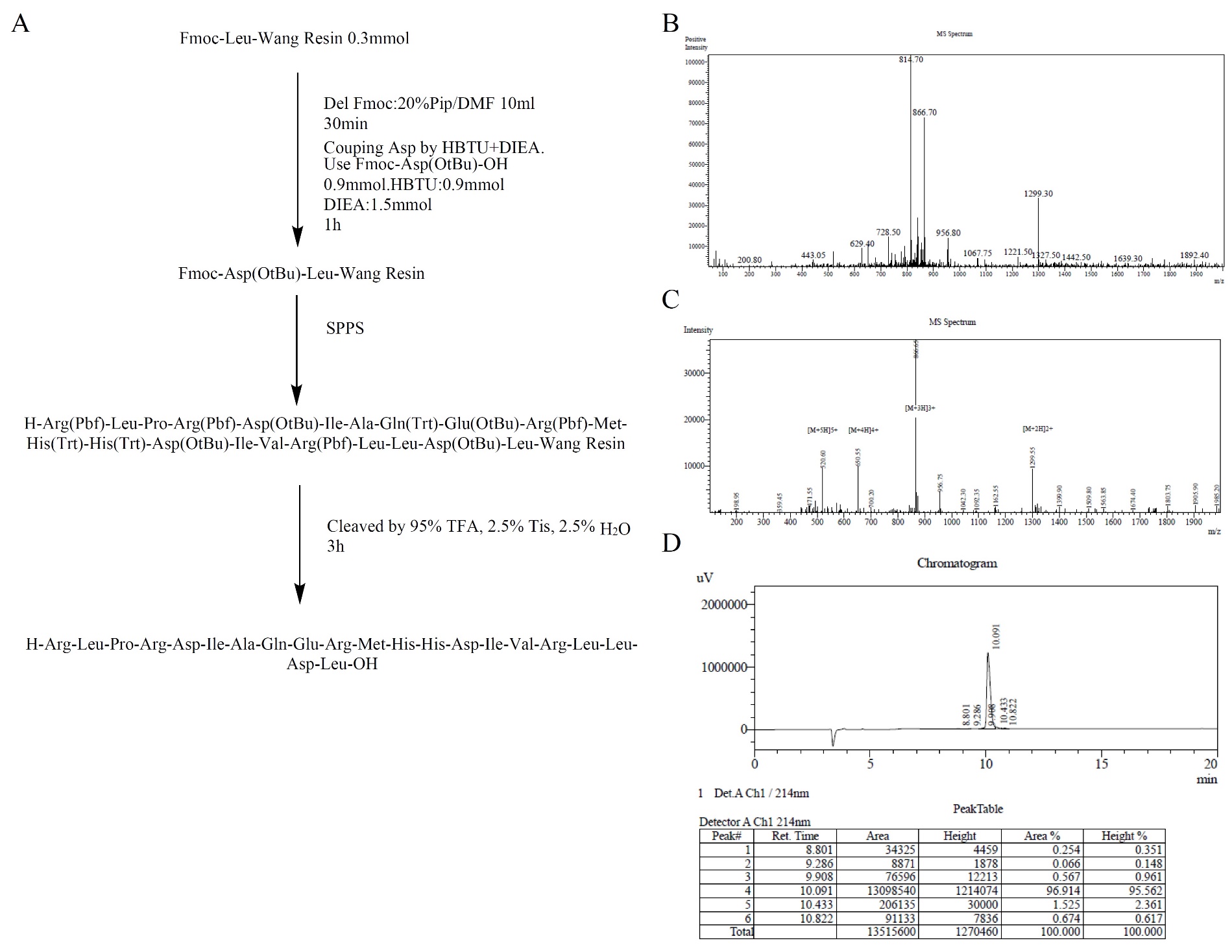


**Figure S25.** Synthesis process of NICD-7l peptide, related to Figure 2. (A) Flow chart of peptide synthesis. (B) Mass spectrometry results of crude peptide. (C) Mass spectrometry results of purified peptide. (D) The purity of the peptide was detected by high-performance liquid chromatography.


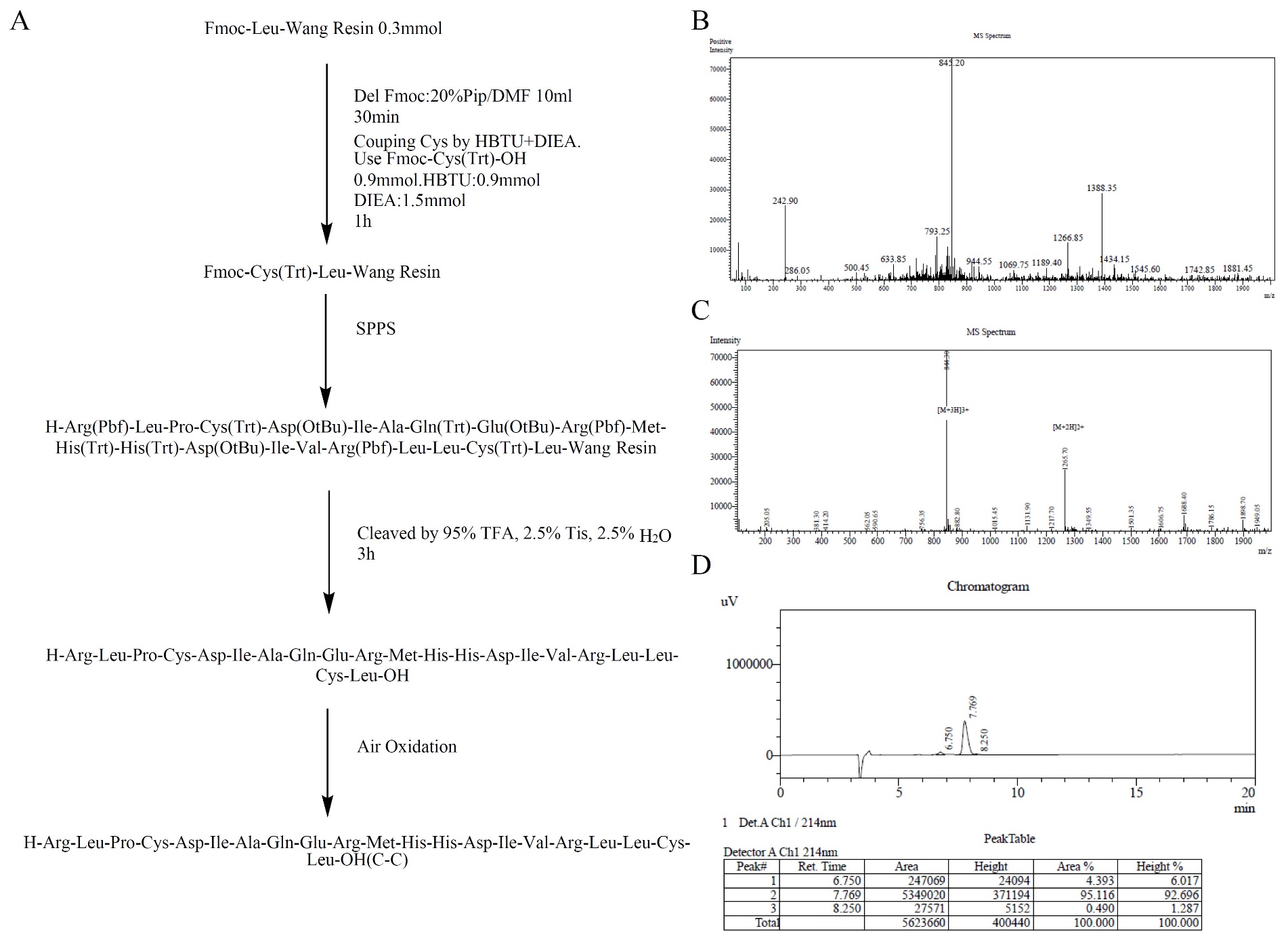


**Figure S26.** Synthesis process of NICD-7c peptide, related to Figure 2. (A) Flow chart of peptide synthesis. (B) Mass spectrometry results of crude peptide. (C) Mass spectrometry results of purified peptide. (D) The purity of the peptide was detected by high-performance liquid chromatography.

**
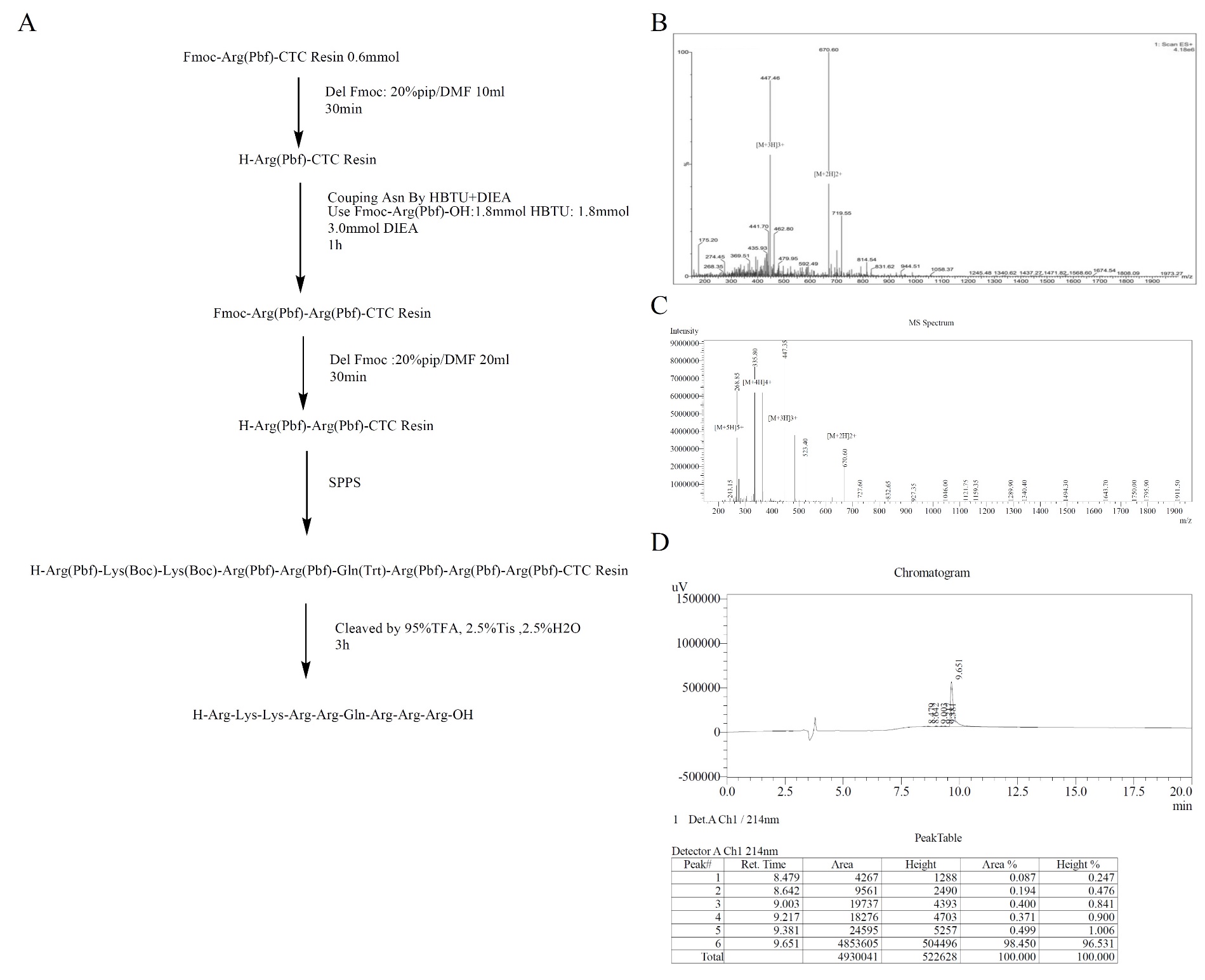
**

**Figure S27.** Synthesis process of TAT peptide, related to Figure 2. (A) Flow chart of peptide synthesis. (B) Mass spectrometry results of crude peptide. (C) Mass spectrometry results of purified peptide. (D) The purity of the peptide was detected by high-performance liquid chromatography.

**
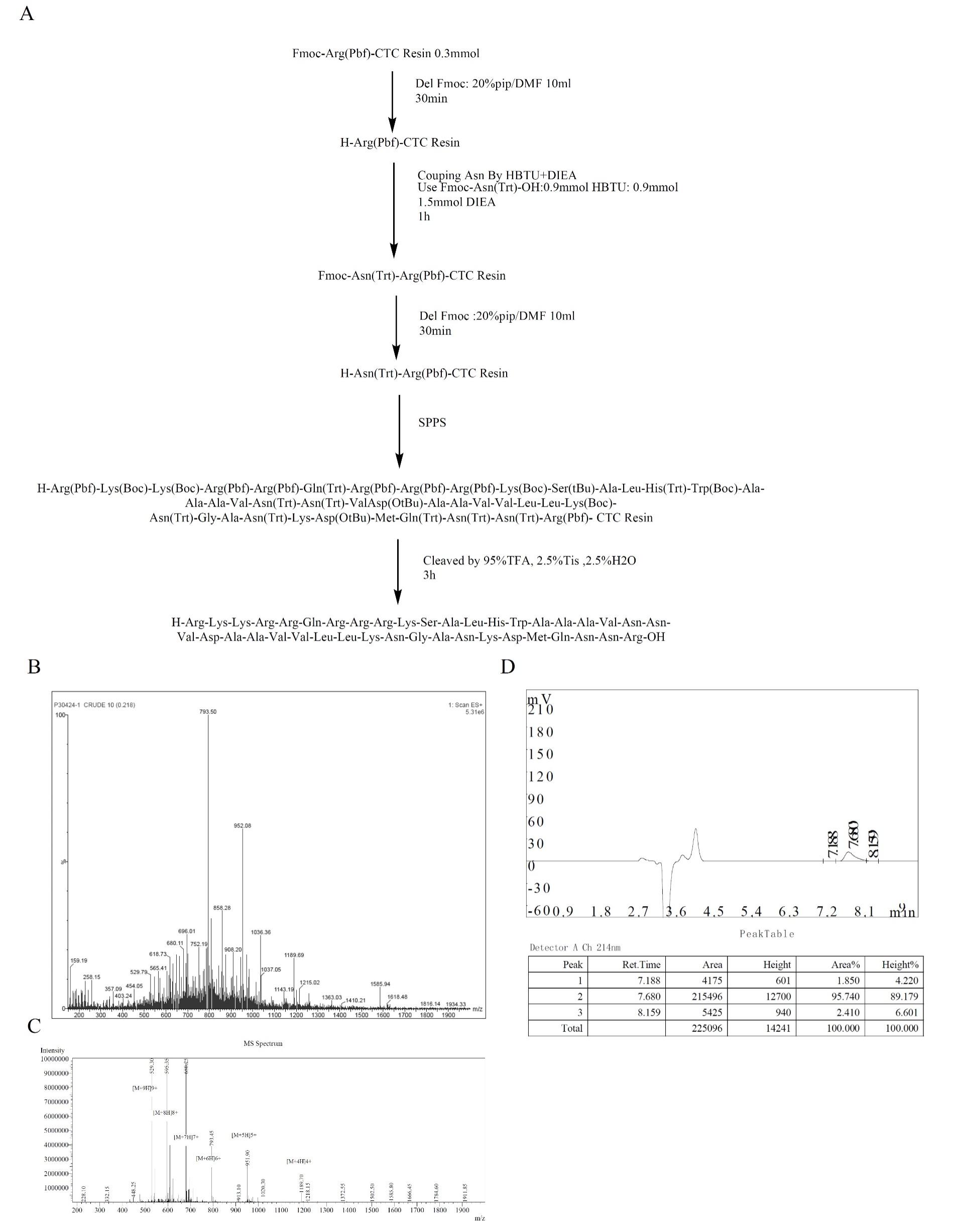
**

**Figure S28.** Synthesis process of TAT-NICD-5l peptide, related to Figure 2. (A) Flow chart of peptide synthesis. (B) Mass spectrometry results of crude peptide. (C) Mass spectrometry results of purified peptide. (D) The purity of the peptide was detected by high-performance liquid chromatography.


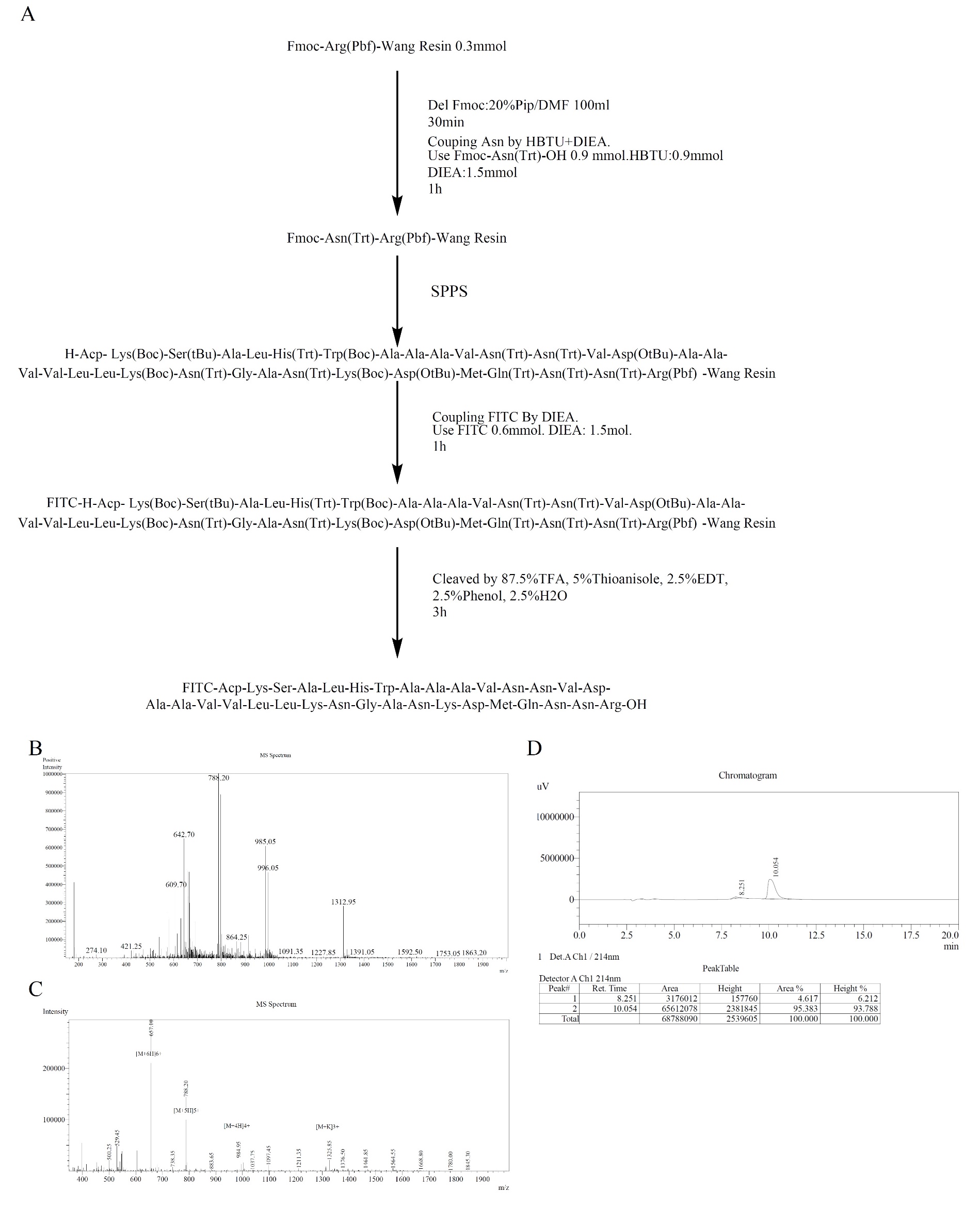


**Figure S29.** Synthesis process of FITC-NICD-5l peptide, related to Figure 2. (A) Flow chart of peptide synthesis. (B) Mass spectrometry results of crude peptide. (C) Mass spectrometry results of purified peptide. (D) The purity of the peptide was detected by high-performance liquid chromatography.


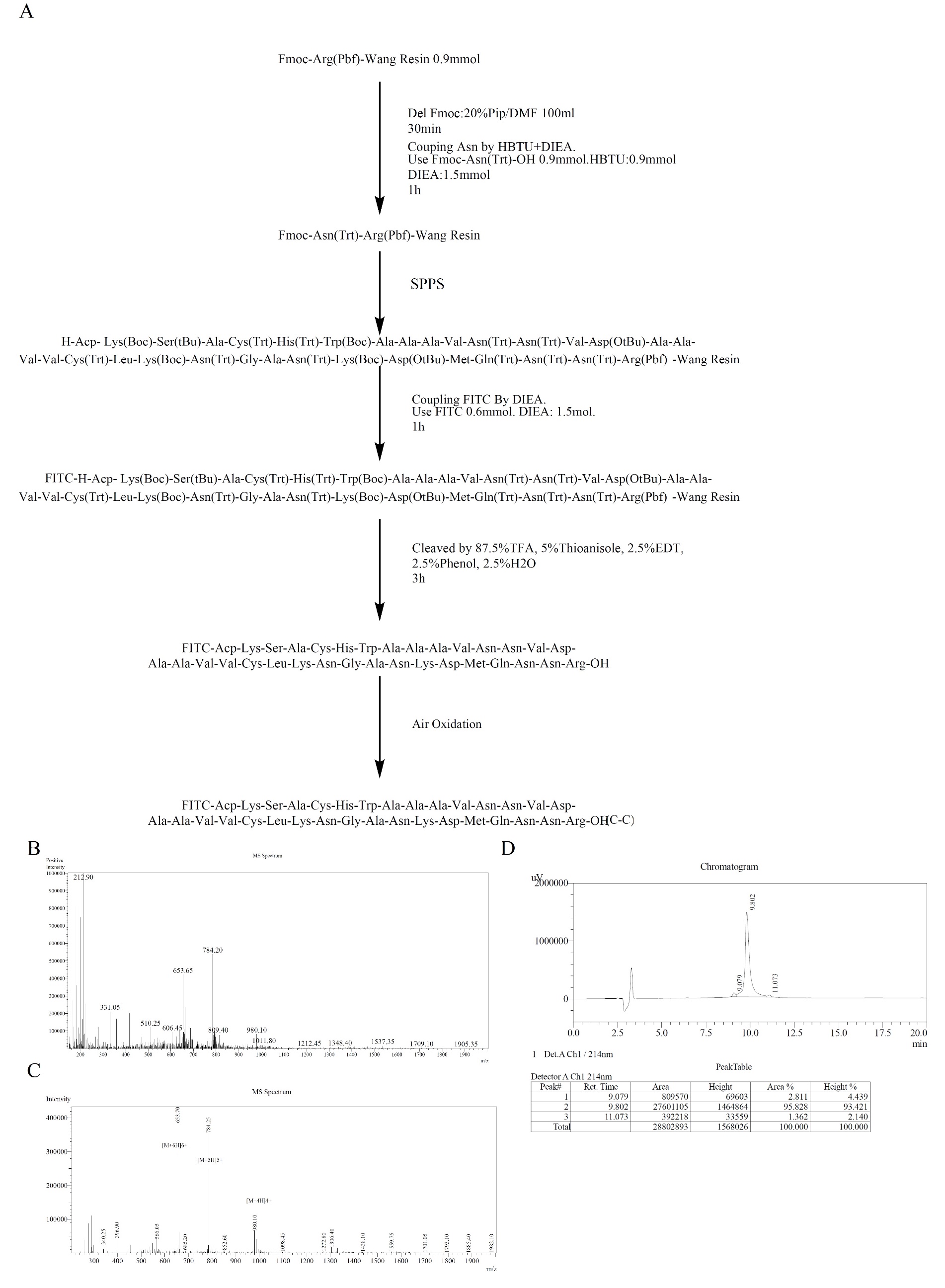


**Figure S30.** Synthesis process of FITC-NICD-5c peptide, related to Figure 2. (A) Flow chart of peptide synthesis. (B) Mass spectrometry results of crude peptide. (C) Mass spectrometry results of purified peptide. (D) The purity of the peptide was detected by high-performance liquid chromatography.

**
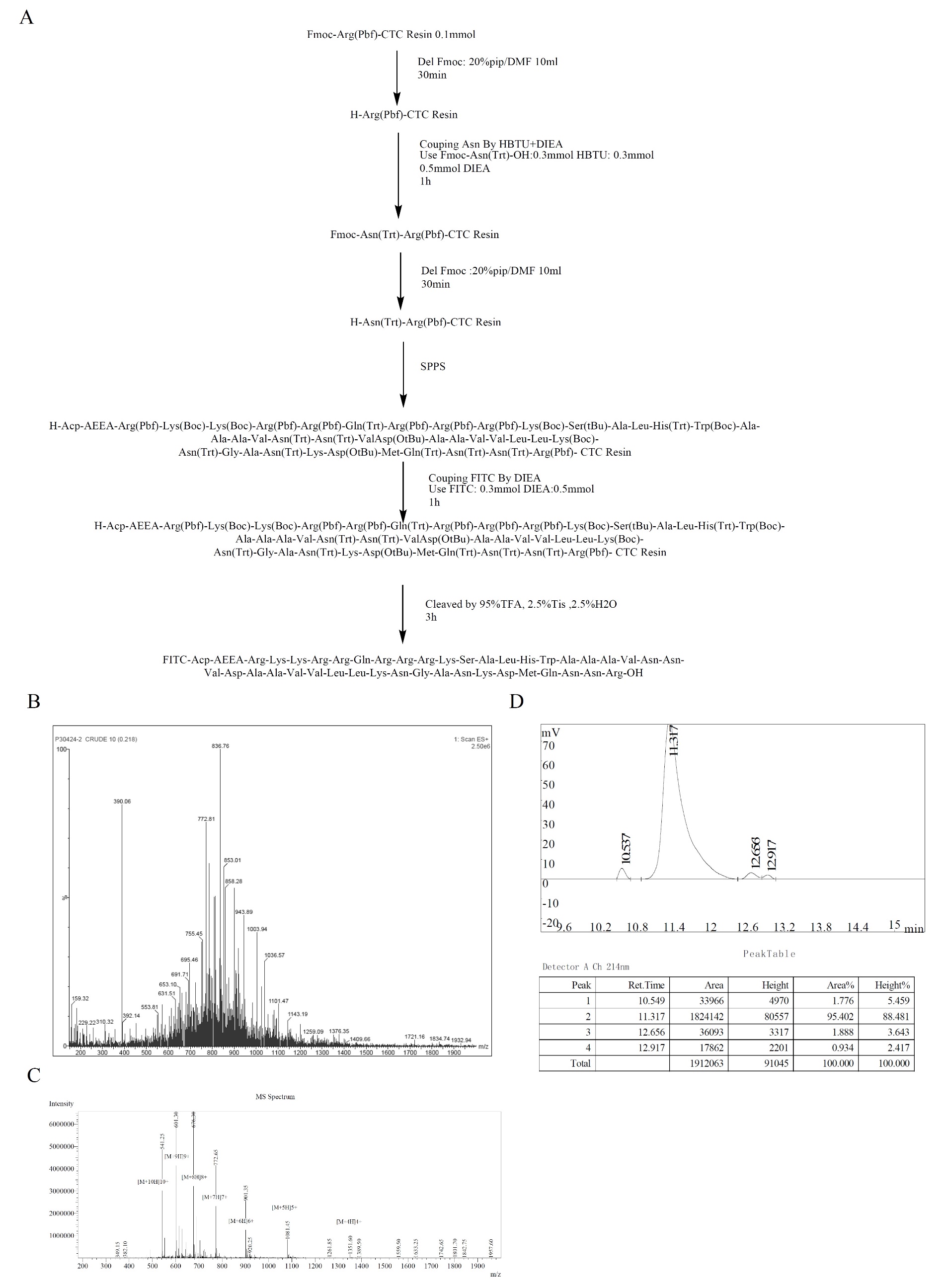
**

**Figure S31.** Synthesis process of FITC-TAT-NICD-5l peptide, related to Figure 2. (A) Flow chart of peptide synthesis. (B) Mass spectrometry results of crude peptide. (C) Mass spectrometry results of purified peptide. (D) The purity of the peptide was detected by high-performance liquid chromatography.

**3. Supplementary Tables**

**Table S1.** Manders overlap coefficient of the regions of interest in colocalization images, related to Figure 2C.

| **Manders overlap coefficient** | **A cell of regions of interest** | | | |
| --- | --- | --- | --- | --- |
|  | **KG1a** | **HL60** | **KG1a-shANXA1** | **HL60-shANXA1** |
| R/G^a)^ | 0.634 ± 0.038 | 0.815 ± 0.089 | 0.137 ± 0.039 | 0.226 ± 0.087 |
| G/R^b)^ | 0.700 ± 0.020 | 0.868 ± 0.013 | 0.887 ± 0.026 | 0.985 ± 0.007 |
| R/B^c)^ | 0.485 ± 0.026 | 0.436 ± 0.093 | 0.742 ± 0.021 | 0.559 ± 0.028 |
| B/R^d)^ | 0.606 ± 0.065 | 0.644 ± 0.058 | 0.810 ± 0.037 | 0.937 ± 0.015 |
| G/B^e)^ | 0.298 ± 0.049 | 0.159 ± 0.065 | 0.918 ± 0.017 | 0.454 ± 0.053 |
| B/G^f)^ | 0.399 ± 0.049 | 0.220 ± 0.082 | 0.154 ± 0.043 | 0.138 ± 0.051 |

^a)^R/G: Fraction of red overlapping green; ^b)^G/R: Fraction of green overlapping red; ^c)^R/B: Fraction of red overlapping blue; ^d)^B/R: Fraction of blue overlapping red; ^e)^G/B: Fraction of green overlapping blue; ^f)^B/G: Fraction of blue overlapping green.

**Table S2.** Kinetic parameters for BLI analyses, related to Figure 2E,I.

| **Sample** | **K_D_ (M)** | **K_on_ (1 Ms^-1^)** | **K_off_ (1 s^-1^)** |
| --- | --- | --- | --- |
| Full-length ANXA1 | 1.84×10^-5^ | 1.57×10^3^ | 2.88×10^-2^ |
| C-terminal domain of ANXA1 | 5.32×10^-6^ | 3.15×10^3^ | 1.68×10^-2^ |
| NICD-5l peptide | 1.54×10^-4^ | 8.77×10 | 1.27×10^-2^ |
| NICD-5c peptide | 2.23×10^-4^ | 4.83×10 | 1.08×10^-2^ |

**Table S3.** The information on the location and sequences of designed NICD peptides, related to Figure 2G; Figure S5A (Supporting Information).

| **Simulation Fragment** | **Location** |  | **Peptide sequences** | **Molecular Weight** |
| --- | --- | --- | --- | --- |
| NICD-2 | 1929~1952 | 2l^a)^ | ETALHLAARYSRSDAAKRLLEASA | 2600.90 |
|  |  | 2c^b)^ | ETAc[CHLAARYSRSDAAKRC]LEASA | 2578.87 |
| NICD-3 | 1963~1984 | 3l | TPLHAAVSADAQGVFQILIRNR | 2377.72 |
|  |  | 3c | TPc[CHAAVSADAQGVFQIC]IRNR | 2355.69 |
| NICD-4 | 1996~2019 | 4l | TTPLILAARLAVEGMLEDLINSHA | 2548.98 |
|  |  | 4c | TTPc[CILAARLAVEGMLEDC]INSHA | 2526.95 |
| NICD-5 | 2029~2060 | 5l | KSALHWAAAVNNVDAAVVLLKNGANKDMQNNR | 3433.88 |
|  |  | 5c | KSAc[CHWAAAVNNVDAAVVC]LKNGANKDMQNNR | 3411.85 |
| NICD-6 | 2062~2085 | 6l | ETPLFLAAREGSYETAKVLLDHFA | 2679.01 |
|  |  | 6c | ETPc[CFLAAREGSYETAKVC]LDHFA | 2656.98 |
| NICD-7 | 2095~2115 | 7l | RLPRDIAQERMHHDIVRLLDL | 2597.04 |
|  |  | 7c | RLPc[CDIAQERMHHDIVRLLC]L | 2530.05 |

^a)^l: Linear peptides; ^b)^c: Cyclic peptides.

**Table S4.** The information of acute myeloid leukemia patients, related to Figure 5 and 6Q.

| **Patient** | **Sex** | **Age** | **WBC**^a)^  **(×10^9^/L)** | **Stage** |
| --- | --- | --- | --- | --- |
| 1 | female | 43 | 2.4 | M5 |
| 2 | male | 69 | 5.4 | M5 |
| 3 | male | 42 | 1.8 | M2 |
| 4 | male | 27 | 2.57 | M4E0 |
| 5 | male | 79 | 17.02 | M2 |
| 6 | male | 54 | 0.6 | M0 |
| 7 | male | 57 | 27.5 | M4E0 |
| 8 | male | 27 | 10 | M3 |
| 9 | female | 29 | 15.65 | M2a |
| 10 | male | 27 | 33.8 | M5b |
| 11 | male | 45 | 2.1 | M5 |
| 12 | male | 61 | 11.8 | AML |
| 13 | male | 27 | 39.8 | M3 |
| 14 | female | 75 | 4.6 | AML |
| 15 | female | 64 | 6.8 | M3a |
| 16 | female | 48 | 17.19 | M2a |
| 17 | female | 69 | 4.1 | M2a |
| 18 | male | 73 | 0.4 | M5 |
| 19 | male | 70 | 225 | M5 |
| 20 | female | 75 | 7.4 | M5 |
| 21 | male | 70 | 12.7 | M2a |
| 22 | male | 75 | 1.3 | M2 |
| 23 | male | 67 | 70.97 | M5 |
| 24 | female | 60 | 50 | M2 |
| 25 | female | 76 | 46.26 | M5 |
| 26 | female | 40 | 7.25 | M2a |
| 27 | male | 67 | 1.62 | M5 |
| 28 | male | 21 | 8.28 | M4 |
| 29 | female | 32 | 124.41 | M2a |

^a)^WBC: White blood cells.

**Table S5.** Contingency table and Fisher's exact test of protein expression and sample source, related to Figure 5.

| **Protein expression** | **Patients** | **Volunteers** | **Fisher's exact test** |
| --- | --- | --- | --- |
| ANXA1 positive^a)^ | 23 | 1 | *p* < 0.0001 |
| ANXA1 negative^b)^ | 1 | 15 |  |
| Notch1 positive | 3 | 16 | *p* < 0.0001 |
| Notch1 negative | 21 | 0 |  |
| p15 positive | 1 | 16 | *p* < 0.0001 |
| p15 negative | 23 | 0 |  |

^a)^Positive: 1, 2 and 3 scores; ^b)^Negative: 0 score.

**Table S6.** The information of antibodies.

| **Antibodies** | **Host** | **Brand** | **Catalog No.** | **Application** | **Notes** |
| --- | --- | --- | --- | --- | --- |
| Normal rabbit IgG | Rabbit | CST^a)^ | 2729 | IP^b)^ | - |
| Anti-human ANXA1 | Rabbit | Bioworld | BS3438 | IF^c)^, ICC^d)^, WB^e)^ | - |
| Anti-human Notch1 | Mouse | Abcam | AB44986 | IF, ICC | - |
| Anti-human Notch1 | Rabbit | Huabio | ET1606-55 | IP, WB | - |
| Anti-human p15 | Rabbit | Sigma | SAB4500078 | ICC, WB | - |
| Anti-human p16 | Rabbit | Bioworld | BS1265 | WB | - |
| Anti-human p21 | Rabbit | Sigma | SAB4500065 | WB | - |
| Anti-human p27 | Rabbit | Merck | 06-445 | WB | - |
| Anti-human Ubiquitin | Rabbit | Proteintech | 10201-2-AP | WB | - |
| Anti-human WWP2 | Rabbit | Abclonal | A2425 | WB | - |
| Anti-human TNF-α | Mouse | Arigo | ARG10158 | WB | - |
| Anti-human CXCR4 | Rabbit | Bioworld | BS90350 | WB | - |
| Anti-human NF-κB | Rabbit | Bioworld | BS1252 | WB | - |
| Anti-human YAP1 | Rabbit | Huabio | ET1608-30 | WB | - |
| Anti-human TAZ | Rabbit | Bioworld | BS5954 | WB | - |
| Anti-human 14-3-3-ε | Rabbit | Bioworld | BS2177 | WB | - |
| Anti-human 14-3-3-γ | Rabbit | Bioworld | BS2512 | WB | - |
| Anti-human ERK1/2 | Rabbit | Bioworld | BS1112 | WB | - |
| Anti-human p-ERK1/2 | Rabbit | CST | 4370 | WB | - |
| Anti-human TEAD2 | Rabbit | Bioworld | BS61393 | WB | - |
| Anti-human MST1/2 | Rabbit | Bioworld | BS1554 | WB | - |
| Anti-human Smad2 | Rabbit | Bioworld | AP0444 | WB | - |
| Anti-human GAPDH | Rabbit | Bioworld | AP0063 | WB | - |
| Anti-human Tubulin α | Rabbit | Huabio | ER130905 | WB | - |
| Anti-rabbit IgG | Goat | Bioworld | BS13278 | ICC, WB | HRP-conjugated |
| Anti-mouse IgG | Goat | Bioworld | BS12478 | ICC, WB | HRP-conjugated |
| Anti-rabbit IgG | Goat | Huabio | HA1004 | IF | FITC-conjugated |
| Anti-mouse IgG | Goat | Huabio | HA1017 | IF | Rhodmine-conjugated |
| Anti-rabbit IgG | Alpacos | NB biolab | NBI01H | WB | HRP-conjugated  IP Nano-secondary antibody |
| Anti-human CD45 | Mouse | Ebioscience | 11-0459-42 | FCM^f)^ | FITC-conjugated |
| Anti-mouse CD45 | Rat | Ebioscience | 12-0451-82 | FCM | PE-conjugated |

^a)^CST: Cell Signaling Technology; ^b)^IP: immunoprecipitation; ^c)^IF: immunofluorescence; ^d)^ICC: immunocytochemistry; ^e)^WB: western blotting; ^f)^FCM: flow cytometry.

**Supporting references**

[1] a) D. Kozakov, D. Beglov, T. Bohnuud, S. E. Mottarella, B. Xia, D. R. Hall, S. Vajda, *Proteins* **2013**, *81* (12), 2159; b) D. Kozakov, D. R. Hall, B. Xia, K. A. Porter, D. Padhorny, C. Yueh, D. Beglov, S. Vajda, *Nat Protoc* **2017**, *12* (2), 255; c) S. Vajda, C. Yueh, D. Beglov, T. Bohnuud, S. E. Mottarella, B. Xia, D. R. Hall, D. Kozakov, *Proteins* **2017**, *85* (3), 435; d) I. T. Desta, K. A. Porter, B. Xia, D. Kozakov, S. Vajda, *Structure* **2020**, *28* (9), 1071.

[2] M. Mirdita, K. Schutze, Y. Moriwaki, L. Heo, S. Ovchinnikov, M. Steinegger, *Nat Methods* **2022**, *19* (6), 679.

[3] J. Rappsilber, M. Mann, Y. Ishihama, *Nat protoc* **2007**, *2* (8), 1896.

[4] Z. L. Chen, J. M. Meng, Y. Cao, J. L. Yin, R. Q. Fang, S. B. Fan, C. Liu, W. F. Zeng, Y. H. Ding, D. Tan, L. Wu, W. J. Zhou, H. Chi, R. X. Sun, M. Q. Dong, S. M. He, *Nat Commun* **2019**, *10*, 3404.
